# The TPR domain of PgaA is a multifunctional scaffold that binds PNAG and modulates PgaB-dependent polymer processing

**DOI:** 10.1371/journal.ppat.1010750

**Published:** 2022-08-05

**Authors:** Roland Pfoh, Adithya S. Subramanian, Jingjing Huang, Dustin J. Little, Adam Forman, Benjamin R. DiFrancesco, Negar Balouchestani-Asli, Elena N. Kitova, John S. Klassen, Régis Pomès, Mark Nitz, P. Lynne Howell

**Affiliations:** 1 Program in Molecular Medicine, The Hospital for Sick Children, Toronto, Ontario, Canada; 2 Department of Biochemistry, University of Toronto, Toronto, Ontario, Canada; 3 Department of Chemistry, University of Toronto, Toronto, Ontario, Canada; 4 Department of Chemistry, University of Alberta, Edmonton, Alberta, Canada; Children’s Hospital Boston, UNITED STATES

## Abstract

The synthesis of exopolysaccharides as biofilm matrix components by pathogens is a crucial factor for chronic infections and antibiotic resistance. Many periplasmic proteins involved in polymer processing and secretion in Gram-negative synthase dependent exopolysaccharide biosynthetic systems have been individually characterized. The operons responsible for the production of PNAG, alginate, cellulose and the Pel polysaccharide each contain a gene that encodes an outer membrane associated tetratricopeptide repeat (TPR) domain containing protein. While the TPR domain has been shown to bind other periplasmic proteins, the functional consequences of these interactions for the polymer remain poorly understood. Herein, we show that the C-terminal TPR region of PgaA interacts with the de-*N-*acetylase domain of PgaB, and increases its deacetylase activity. Additionally, we found that when the two proteins form a complex, the glycoside hydrolase activity of PgaB is also increased. To better understand structure-function relationships we determined the crystal structure of a stable TPR module, which has a conserved groove formed by three repeat motifs. Tryptophan quenching, mass spectrometry analysis and molecular dynamics simulation studies suggest that the crystallized TPR module can bind PNAG/dPNAG via its electronegative groove on the concave surface, and potentially guide the polymer through the periplasm towards the porin for export. Our results suggest a scaffolding role for the TPR domain that combines PNAG/dPNAG translocation with the modulation of its chemical structure by PgaB.

## Author summary

Exopolysaccharides are an important component of the extracellular matrix of bacterial and fungal biofilms and provide protection against the host immune response and antibiotics. In Gram-negative bacteria, these polymers are synthesized in the inner membrane and translocated across the periplasm before being secreted across the outer membrane. The periplasm presents both a challenge as an additional environment to cross and an opportunity to chemically alter the polymer prior to secretion to render it more effective. This study focuses on a periplasmic alpha-helical repeat domain whose wide-spread homologues are involved in the export of many chemically distinct exopolysaccharides. We found that in *E*. *coli* this superhelical TPR domain acts as a scaffold that can bind the polymer PNAG and alter the enzymatic activity of PgaB, thus providing a means to affect the deacetylation level and chain length of the secreted polymer. Scaffold proteins are known as binding hubs within cellular pathways that often have a central regulatory function and facilitate evolution due to their repetitive modular building blocks. Our study sheds light on the principles of polysaccharide modification and export, which we hope will promote the development of applications against bacterial infections.

## Introduction

**Fig 1:**
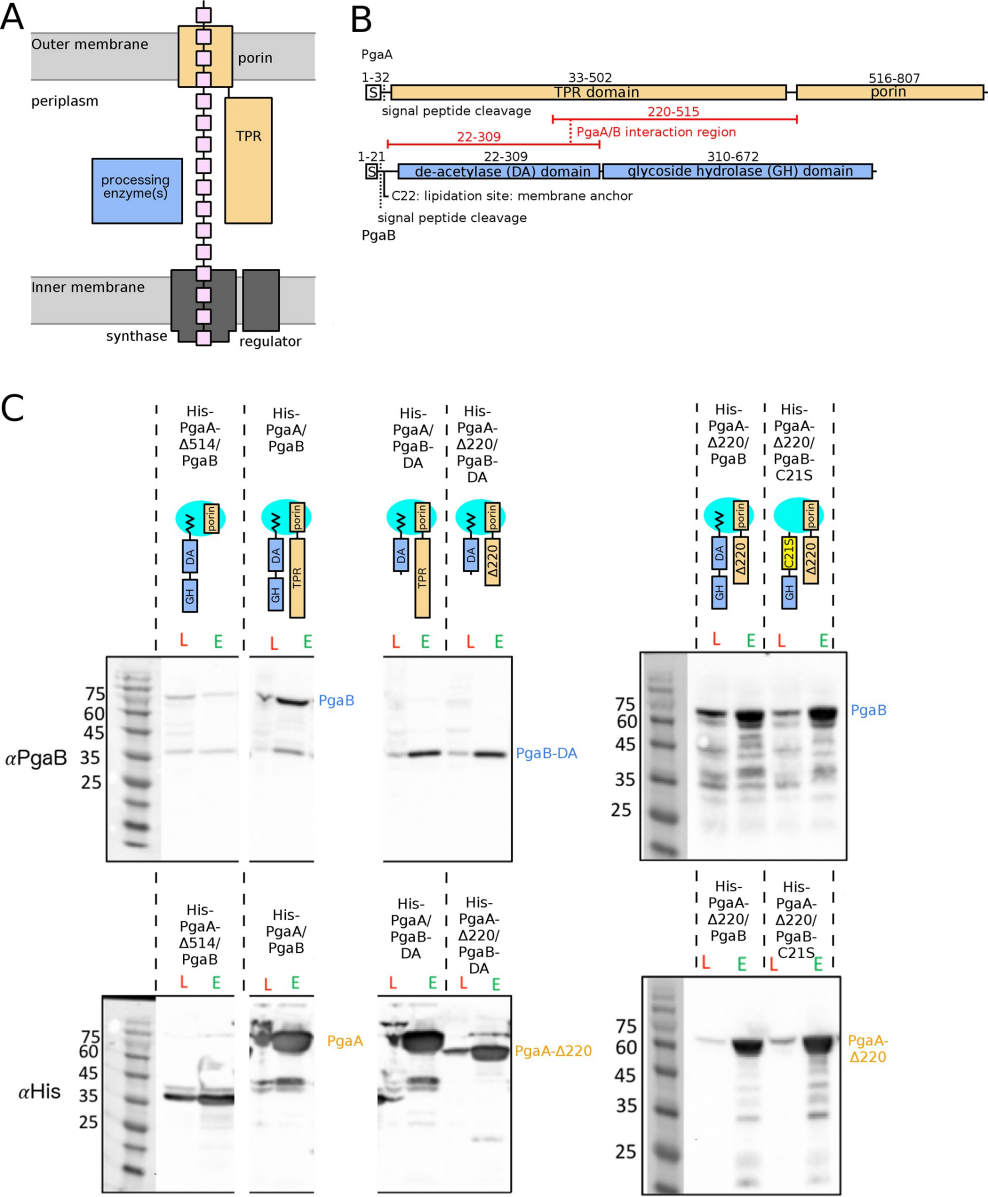
Residues 32–220 of PgaA and the glycoside hydrolase domain of PgaB are dispensable for PgaA/B interaction. A) Generalized synthase-dependent schematic for the production and secretion of exopolysaccharides in Gram-negative bacteria. B) PgaA and PgaB domain topology. The regions determined to interact are highlighted in red. C) Western blot analysis of Ni-pulldown assays with various N-terminally His-tagged PgaA constructs. PgaA and PgaB were co-expressed with each protein construct containing a signal sequence for periplasmic translocation. PgaA and PgaB constructs are depicted in the anticipated orientation with the membrane anchor and the porin positioned in the outer membrane, indicated in cyan. Lysate fractions (red L) and elution fractions (green E) are indicated for each lane. Western blots from the pulldown experiments were probed with a monoclonal primary mouse anti-his (Abgent) or a rabbit polyclonal primary antibody raised against PgaB. The molecular weight markers (kDa) are indicated on the left of each panel.

Exopolysaccharides in bacterial and fungal biofilms are implicated in chronic infections and antibiotic resistance; and thus pose a threat to human health. In Gram-negative bacteria three pathways have been identified for the production and secretion [1] of exopolysaccharides: the ABC transporter-, Wzx/Wzy-, and synthase-dependent pathways. Cellulose, alginate, the Pel polysaccharide, and poly-β(1,6)-*N*-acetylglucosamine (PNAG) are each produced by the synthase-dependent pathway. Operons of synthase-dependent systems in Gram-negative bacteria contain a core set of proteins in the inner membrane for polymerization and transport into the periplasm, one or more periplasmic exopolysaccharide-modifying enzymes, and an outer membrane β-barrel porin for export (**Fig 1A**). Common to each system is also the presence of an outer membrane associated α/α-repeat domain. In the cellulose, Pel, and PNAG biosynthetic systems this α/α-repeat domain is part of the porin, BcsC, PelB, PgaA, respectively, while in the alginate system it is a separate outer-membrane lipid anchored protein, AlgK [1].

The α/α-repeats found in these proteins can be divided into two classes: tetratricopeptide repeats (TPRs), and Sel1-like repeats (SLRs). SLRs are a closely related subgroup of TPRs and both α/α-repeats form superhelical structures. The TPR superhelix is usually narrower with a pitch of seven repeats whereas a SLR superhelix is wider with a pitch of eight to nine repeats [2]. In the alginate system, AlgK was found to be a 70 Å long superhelix formed by 9.5 SLRs [3]. In contrast, while BcsC and PelB are each predicted to contain 19 TPR motifs, structural data is only available for short fragments of both proteins, 6 [4] and 3.5 [5] TPR motifs, respectively. In the case of BcsC, a single alpha helix was found inserted between two continuous TPR modules, potentially providing a flexible hinge. Small angle X-ray scattering studies of BcsC also suggest that this protein may adopt an extended conformation with at least one hinge point [4]. To date, no structural data is available for the TPR domain of PgaA, which is predicted to contain 8 TPR motifs [6].

AlgK, PelB, BcsC and PgaA have been proposed to be hub proteins that interact with other components of the synthase system and in some cases may aid in the formation of a transmembrane complex for polysaccharide transport across the periplasm. Pull-down and cellular fractionation data suggests AlgK interacts with the acetyltransferase AlgX, the outer membrane porin AlgE, as well as the inner membrane cyclic-di-GMP receptor Alg44 [7]. In the PNAG system, *in vivo* studies in *Yersinia pestis* have demonstrated that PgaA interacts with PgaB (formerly annotated as HmsH and HmsF, respectively) [8]. Subsequent studies in *Escherichia coli* localized this interaction to the periplasmic TPR domain of PgaA [6]. In both *Y*. *pestis* and *E*. *coli*, PgaB contains an N-terminal signal sequence and outer-membrane lipid anchor, an N-terminal de-*N-*acetylase domain [9,10] and a C-terminal glycoside hydrolase domain [11]. We have also recently demonstrated that the TPR domain of *Pseudomonas aeruginosa* PelB interacts with the dual functional enzyme PelA [5], which like PgaB exhibits both de-*N-*acetylase and glycoside hydrolase activity. At present the molecular level details of how these α/α-repeat domain proteins in the alginate, Pel, cellulose and PNAG systems interact with their respective protein partner(s) and/or exopolysaccharides has not been characterized.

During our investigation of PelB-PelA, we demonstrated that this interaction altered the de-*N-*acetylase and glycoside hydrolase activities of PelA. This modulation of enzymatic activity suggests that this interaction is not passive, but has consequences for the chemical composition and length of the polymer. These data suggest that PelB’s TPR domain is not just a hub protein or structural platform, but functions more like the scaffold proteins found in intracellular signaling cascades [12]. Although functionally and structurally diverse, scaffold proteins share several hallmarks that make them conceptually similar: they are usually formed by repeating structural motifs or modules, can bind two or more members of a pathway, localize at a specific region, and can alter the enzymatic activities of their binding partners. The modular nature of scaffold proteins has been suggested to provide advantages during evolution by recruiting existing components (e.g. processing enzymes) for novel purposes [13], which might explain their presence in synthase-dependent pathways producing chemically different exopolysaccharides.

Despite the wealth of data that exists for the alginate, Pel, cellulose and PNAG biosynthetic systems, the overall mechanism of how the polymer is processed in the periplasm remains poorly understood. With the hypothesis that the TPR domain is the central interaction scaffold we set out to characterize the functionalities of this domain in the *E*. *coli* PNAG biosynthetic system. This system provides a good model for understanding periplasmic processing in synthase-dependent pathways as there are only four proteins involved. Our ability to synthesize defined length partially deacetylated PNAG (dPNAG) oligomers also enables us to probe not only protein-protein interactions, but also protein-polysaccharide interactions [14]. Herein, we show that the TPR region proximal to the outer membrane porin interacts with the N-terminal de-*N-*acetylation domain of PgaB, and that this interaction increases the deacetylation activity of PgaB similar to the PelA/PelB complex in the Pel system. The glycoside hydrolase activity of PgaB is also increased in the PgaA/PgaB complex, but only when the N-terminal TPR region (PgaA-1-220) is co-expressed. The crystal structure of PgaA-220-367 revealed a curved TPR module with grooves involving conserved residues. Subsequent analysis of the crystallized module by tryptophan quenching, mass spectrometry, and molecular dynamics (MD) simulations suggests that the polymer binds to the convex surface of the module. Furthermore, our modeling of full-length PgaA suggests that PgaA contains at least 13 α/α-repeats that connect to the outer-membrane porin. Collectively, the TPR domain of PgaA has characteristics of an assembly-line scaffold [12], which enables the passage of a substrate (PNAG) between different domains (PgaB de-acetylase domain and/or PgaB glycoside hydrolase domain, and PgaA porin domain) and in addition modulates enzymatic activities (de-acetylase and glycoside hydrolase activity).

## Results

### The outer membrane-proximal TPR region of PgaA interacts with the deacetylase domain of PgaB

Previous studies have established that PgaA and PgaB form a complex at the outer membrane and that the TPR domain of PgaA is critical for this interaction [6,8]. To further probe the role of PgaA as a scaffold protein and to understand the consequences of this interaction, we first sought to delineate which region(s) of each protein are required for the interaction. Both PgaA and PgaB are multi-domain proteins. PgaA contains an N-terminal domain with 8 predicted TPR motifs and a C-terminal porin domain, while PgaB is an outer-membrane lipoprotein with an N-terminal de-acetylase (DA) domain and C-terminal glycoside hydrolase (GH) domain (**Fig 1B**). We co-expressed the full-length His-tagged PgaA (His-PgaA) and untagged PgaB and various truncations of each protein in *E*. *coli* and used nickel affinity purification and western blot analysis to analyze interacting partners (**Fig 1C**). As anticipated, we found that when we co-expressed full-length His-PgaA and PgaB, we observed a strong signal for PgaB using a PgaB-specific antibody, indicating that the His-tagged PgaA had successfully pulled-down PgaB. As PgaB has the tendency to degrade over time, we also observed lower molecular weight fragments in the Western blot. As a negative control, we used a construct of the porin domain of PgaA (His-PgaA-Δ514, residues 515–807, **Fig 1B**) and found a minimal signal for PgaB.

In an attempt to simplify the analysis, we next tried to use the TPR domain of PgaA alone (residues 32–515, **Fig 1B**) to pull down PgaB. This was unsuccessful as we could not detect His-PgaA-32-515 in the eluted fraction (**S1A Fig**). We assume this is due to instability of the isolated TPR domain PgaA-32-515, which we found was prone to proteolytic degradation (**S1B, S1C and S1D Fig**). Mass spectrometry analysis of the purified fragments suggests that each encompasses portions of the N-terminal domain and that the C-terminal region of PgaA-32-515 was degraded during the course of the experiment. As we could not directly examine interactions between the isolated TPR domain of PgaA and PgaB, all subsequent constructs included PgaA’s porin domain.

We next sought to determine which regions of each protein were critical for the interaction. We found that removal of the N-terminal TPR region of PgaA (residues 32–220, His-PgaA-Δ220, **Fig 1C**) did not abrogate the ability of PgaA to pull-down PgaB, suggesting that the C-terminal region of the TPR domain (residues 220–515) is sufficient for the interaction with PgaB. When we truncated PgaB and removed its C-terminal glycoside hydrolase domain, we found that when the lipidated PgaB-DA construct was co-expressed with either full-length PgaA (His-PgaA) or the truncated His-PgaA-Δ220 that PgaB-DA was pulled down. These data suggest that the deacetylase domain alone is sufficient for the interaction between PgaA and PgaB.

During our initial protein interaction studies, we had first explored whether we could express and purify PgaA and PgaB separately, and observe an interaction by mixing the two proteins *in vitro*. Analysis of the PgaA-PgaB mixture using size exclusion chromatograph (SEC) suggested that a stable complex was not formed (**S2A Fig**). Given that the same full-length construct for PgaA (His-PgaA-32-807) was used in the SEC and pull-down assays, we hypothesized that perhaps our inability to observe an interaction after mixing was due to the fact that we had expressed a soluble form of PgaB (PgaB-22-672). Residues 1–20 of PgaB contain a signal sequence that is cleaved during periplasmic targeting and C21 is subsequently lipidated and inserted into the outer membrane. PgaB 22–672 lacks the C21 lipidation site that would be present in the co-expression construct PgaB-1-672. The membrane-anchored form of PgaB could not be used for *in vitro* interaction experiments as it aggregates when purified alone [6]. To probe whether lipidation, and therefore membrane anchoring, is required for the PgaA-PgaB interaction we mutated cysteine 21 to serine, and co-expressed PgaB-1-672-C21S with His-PgaA-221-807. We found that mutation of the cysteine did not affect PgaA’s ability to pull down PgaB (**Fig 1C**), suggesting that membrane anchoring was not required for complex formation. We also explored whether the PgaA/B complex can be obtained via co-purification by mixing cell-pellets in a 1:1 ratio prior to cell lysis (**S2B Fig**). We found that PgaA and PgaB–both PgaB-22-672 and the full-length protein–eluted in a single peak at 11 ml, as we had observed when we co-expressed proteins. Taken together, complex formation appears to require co-expression or co-purification, as we cannot detect the complex when mixing the individually purified proteins. Furthermore, the lipid anchor on PgaB does not appear to be required for complex formation suggesting that PgaB can localize to the outer membrane as a consequence of its interaction with PgaA. As the porin domain does not bind PgaB, our data suggests that the interaction between PgaA and the N-terminal domain of PgaB is localized to residues 221–515 and 22–309, respectively **(Fig 1B**).

### PgaA modulates the deacetylase and glycoside hydrolase activities of PgaB

**Fig 2:**
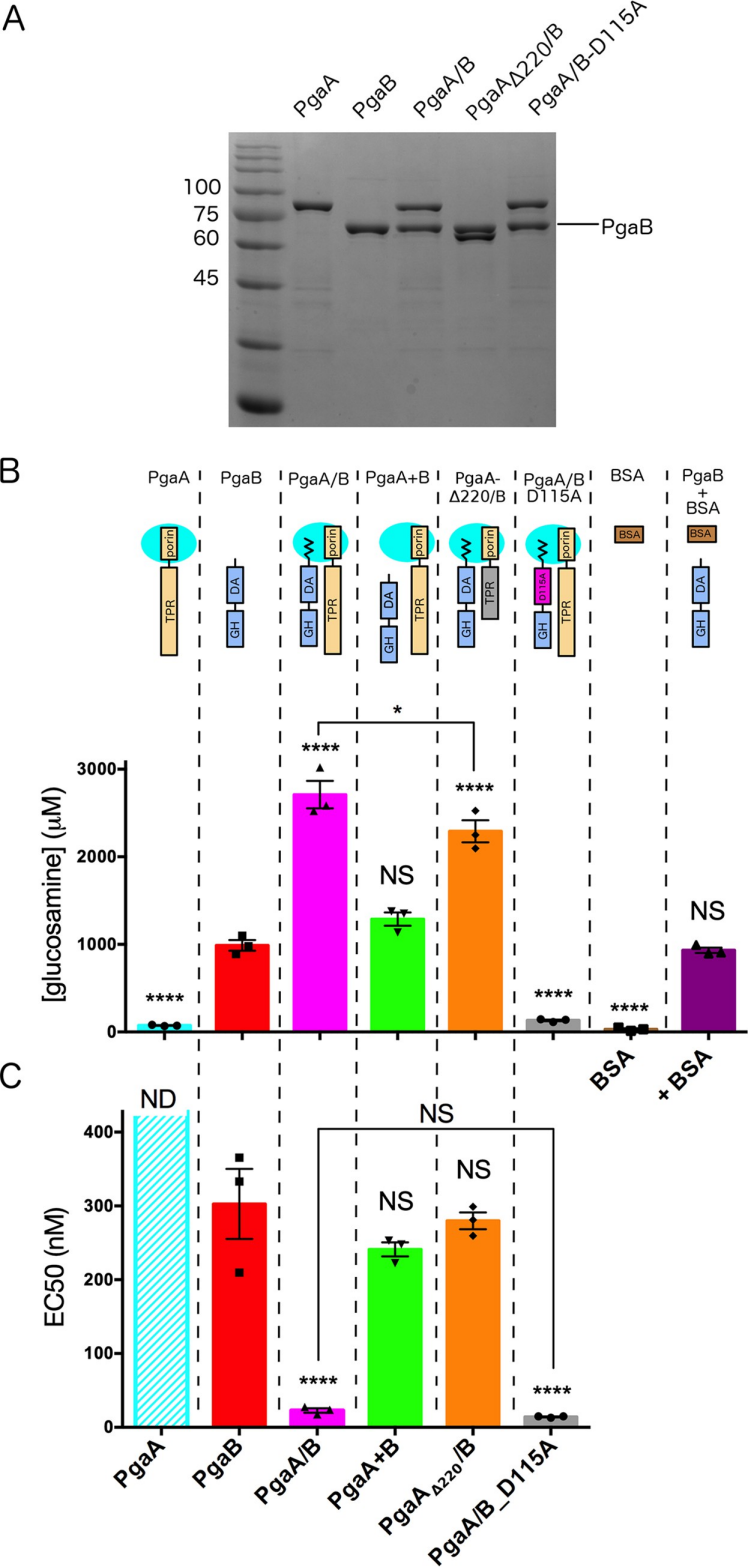
PgaA increases the deacetylase and glycoside hydrolase activities of PgaB. A) Coomassie gels of protein samples used in this experiment, with molecular weight markers (kDa). B) Fluorescamine-base deacetylation assay using 30 μM enzyme and 30 mM PNAG hexamer. The concentration of GlcN produced was as following: PgaA, 73.8 ± 6.3 μM; PgaB, 988.6 ± 104.7 μM; PgaA/B, 2709.0 ± 271.5 μM; PgaA+B, 1288 ± 131.8 μM; PgaA_Δ220_/B), 2291.0 ± 217.3 μM; PgaA/B_D115A, 132.1 ± 16.4 μM; BSA, 31.0 ± 18.1 μM; PgaB+BSA, 932.4 ± 53.4 μM. C) Crystal violet-based biofilm disruption assay using *S*. *epidermidis* SE801. The calculated EC_50_’s for each of the enzymes are as follows: PgaB, 302 ± 82.3 nM; PgaA/B, 22.8 ± 5.1 nM; PgaA+B, 241.1 ± 16.3 nM; PgaA_Δ220_/B, 279.8 ± 19.6 nM; PgaA/B_D115A, 14.2 ± 1.1 nM; ND: not determined. PgaA: PgaA-32-807; PgaB: PgaB-22-672; PgaA/B: complex obtained by co-expression of PgaA-32-807 and PgaB-1-672; PgaA + PgaB: PgaA-32-807 and PgaB-22-672 individually purified and then mixed. Statistical significance is given for comparison against PgaB unless indicated by brackets. ****P ≤ 0.0001, *P ≤ 0.1, NS: no significant difference. Statistical significance was evaluated using two-way analysis of variance and Tukey’s multiple comparison test. Error bars represent the standard error from n = 3 technical replicates.

Recently, we demonstrated in the Pel system that PelB interacts with the periplasmic protein PelA, and that this interaction modulates the *in vitro* deacetylase and hydrolase activities of PelA [5]. To determine whether the modulation of enzyme activity might be a common theme for the TPR domain across synthase-dependent systems, we assayed the deacetylase and glycoside hydrolase activities of PgaB in the presence and absence of PgaA. As we used co-expression to obtain the complexes, a SDS PAGE gel of the purified solutions of PgaA, PgaB and PgaA/B complexes was analyzed to ensure that equal quantities of the protein/protein complex were used (**Fig 2A**). Consistent with previous published studies, PgaA and PgaB appear to form a 1:1 complex [6]. The deacetylase activity was assessed using a fluorescamine-based assay [15] with fully acetylated PNAG hexamers as the substrate. As fluorescamine detects the presence of primary amines, which are generated during the deacetylation of the PNAG oligosaccharide, this assay provides a direct readout of the deacetylation activity of PgaB. To assess the glycoside hydrolase activity, we used a biofilm disruption assay, which has been used previously to characterize PgaB’s glycosidase activity [11]. In this assay, biofilms of *Staphylococcus epidermidis* strain SE801 were grown in 96 well plates for 24 h, at which point PgaB was added. After a further 2 h, the reaction was quenched, plates washes and the amount of adherent biofilm remaining quantified using crystal violet staining. Disruption of the biofilm is due to the cleavage of the PNAG polymer, as mutagenesis of critical PgaB catalytic residues results in not biofilm disruption [11]. It should be noted that in this assay the biofilm matrix will contain partially deacylated PNAG and that a lower EC_50_ value denotes an increase in glycoside hydrolase activity.

When PgaB is in complex with full-length PgaA (PgaA/B in **Fig 2B and 2C**) we found a statistically significant increase in both the deacetylase (2.7-fold) and glycoside hydrolase (13-fold) activities. Consistent with the inability to form a complex upon mixing, no significant changes were observed when the two proteins are purified and then mixed (PgaA+PgaB in **Fig 2B and 2C**). Interestingly, when the N-terminal region of the TPR domain (32–220) is deleted, an increase in de-acetylase activity was still observed but the increase in glycoside hydrolase activity was abolished, suggesting that this region of PgaA influences the C-terminal region of PgaB and assists the hydrolysis reaction. To test whether the increase in glycoside hydrolase activity we observed was due to the higher deacetylation levels of the dPNAG substrate, a PgaB-D115A mutant that abrogates the deacetylase activity was assayed. When PgaB-D115A was co-expressed with PgaA we found no significant difference in glycoside hydrolase activity relative to wild-type PgaA/B (**Fig 2C**), suggesting that the increase in glycoside hydrolase activity is independent of any changes in the deacetylase activity.

In summary, consistent with our complex formation results we found that the deacetylase activity of PgaB is modulated by PgaA-221-807. Our data also reveal that the interaction between the two proteins increases the glycoside hydrolase activity of PgaB and that the increase in EC_50_ observed requires the presence of the N-terminal TPR domain, residues 32–220.

### PgaA-220-342 forms a curved TPR-like module with potential binding grooves

**Fig 3:**
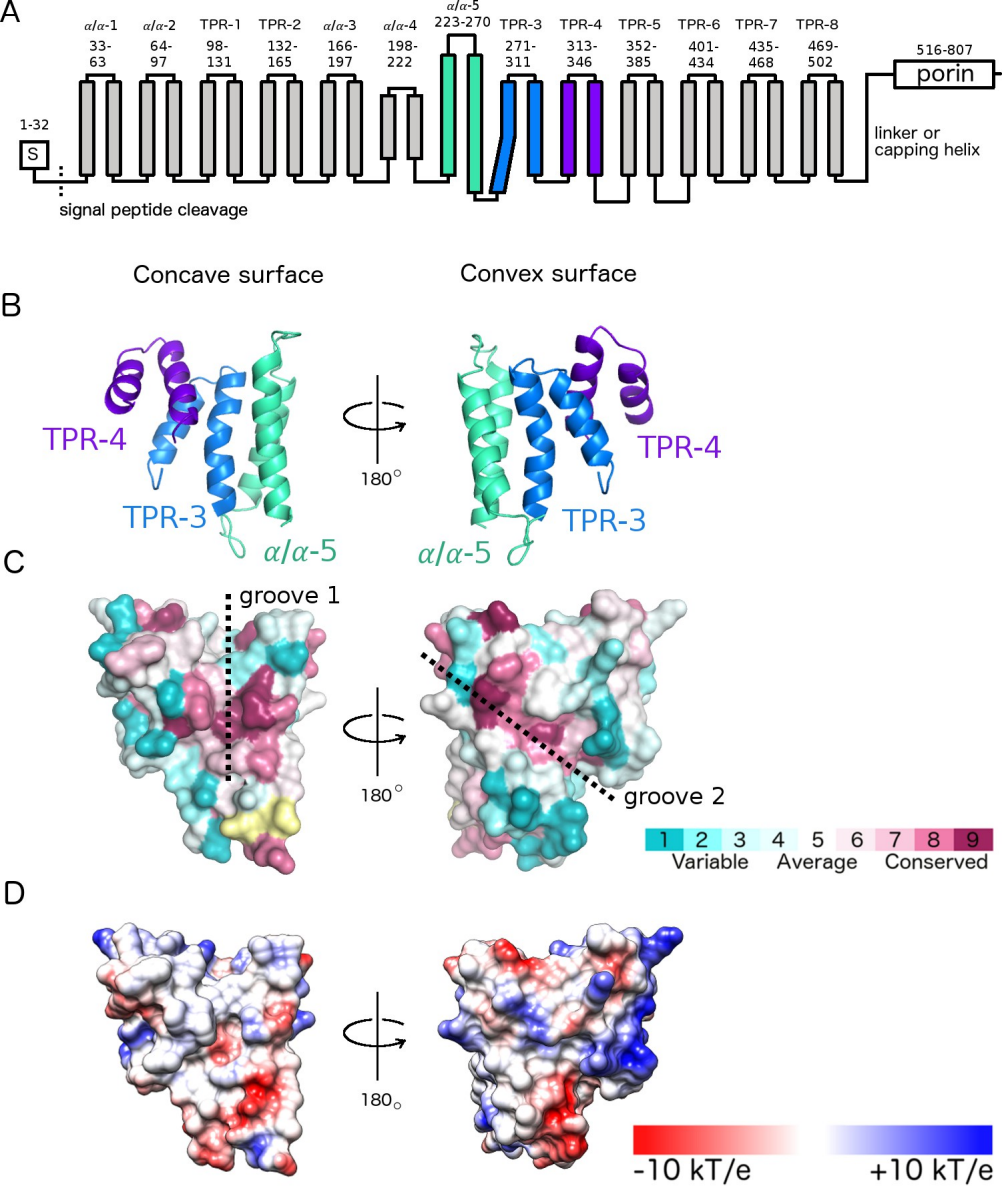
Crystal structure of PgaA 220–367 reveals a curved TPR-like module formed by two TPR motifs and one α/α-repeat. A. Topology model of PgaA including the crystallized module shown in shades of blue. TPR motifs and α/α-repeats were predicted by TPRpred and HHpred [22,23], respectively. B. Crystal structure of PgaA-220-367 shown in cartoon representation and coloured as in panel A. The TPR5 helix is part of a domain swap and has been omitted for clarity. C. Surface representation of the structure mapped with conservation levels as calculated in ConSurf [57] (yellow: insufficient data for conservation analysis of this residue). D. Surface representation with color-coded electrostatics calculated with PyMOL (DeLano Scientific, http://www.pymol.org/).

Previous structural studies of PgaA have been limited to the C-terminal porin domain [6]. To gain insight into its scaffolding role we attempted to crystallize the N-terminal domain of PgaA, which our bioinformatic analyses suggest has five α/α-repeats in addition to the 8 TPR domains predicted previously [6,16] (**Fig 3A**). As described above, our attempts to purify the entire N-terminal domain of PgaA, residues 32–515, resulted in fragmentation of the protein (**S1B, S1C and S1D Fig**). After attempting multiple truncated constructs of the TPR domain (PgaA-32-465, 32–448, 32–431, 32–415, 32–398, 32–382), we found PgaA-32-367 to be the most stable and amenable to crystallization. Diffraction quality crystals were obtained, and the structure was solved using selenomethionine incorporation and the single wavelength anomalous diffraction (SAD) method and refined against a 2.85 Å native dataset (**Table 1**). The asymmetric unit contains two molecules (chains A and B), which were modeled from residues 224–359 and 220–343, respectively. There was no remaining unexplained electron density suggesting that the original construct had further fragmented and that only the C-terminal fragment containing residues 220–359 had crystallized. Chain A contains a long loop region connecting TPR-4 with the N-terminal region of helix 1 of TPR-5. However, helix 1 of TPR-5 interacts with TPR-4 of the symmetry-related molecule (**S3A Fig**). As there is no evidence that PgaA forms a dimer in solution, we presume this domain swap is a crystallization artifact and that only residues 220–342 represent a biologically relevant fold.

**Table 1:** X-ray data collection and refinement statistics.

	SeMet-PgaA-220-367	PgaA-220-367
**Data collection**		
Beamline	NSLS X29A	NSLS X29A
Wavelength (Å)	0.979	0.979
Space group	*P*6_2_	*P*6_2_
Unit-cell parameters (Å)	*a* = *b* = 59.41, *c* = 185.70	*a* = *b* = 60.67, *c* = 185.52
Resolution (Å)	20.0–2.90 (2.90–3.00)*	20.0–2.85 (2.85–2.95)*
Total no. of reflections	166,836	173,938
No. of unique reflections	8212 (773)	8565 (829)
Redundancy	21.4 (18.3)	21.6 (21.8)
Completeness (%)	99.3 (98.5)	99.4 (99.8)
Average *I*/σ(*I*)	28.3 (1.1)	28.8 (1.2)
*R*_merge_^a^ (%)	11.6 (62.3)	12.1 (63.4)
**Anisotropic truncation/scaling**		
Resolution cut-off along 1/*a*, 1/*b*, 1/*c* (Å)		3.00, 3.00, 2.85
Completeness (%) after truncation		92.5 (37.1)
Completeness (%) to 3.9 Å and 4.4 Å		80.2, 99.1
unique reflections after truncation		8362 (247)
Average *I*/σ(*I*) after scaling		28.9 (3.1)
**Refinement**		
*R*_work_^b^ / *R*_free_^c^ (%)		23.5 / 26.4
No. of atoms		
Protein		2146
Water		19
Average B-factor ^d^ (Å^2^)		
Overall		48.6
Protein		48.7
Water		46.6
RMS deviations		
Bond lengths (Å)		0.01
Bond angles (°)		1.43
Ramachandran plot ^d^		
favored/allowed/outlier		234/25/0
Coordinate error ^e^ (Å)		0.34
PDB code		7T8N

^a^ *R*_merge_ = ∑∑ | I (k)—<I>| / ∑ I (k) where I (k) and <I> represent the diffraction intensity values of the individual measurements and the corresponding mean values. The summation is over all unique measurements.

^b^ *R*_work_ = ∑ ||*F*_obs_ |—k|*F*_calc_ || / |*F*_obs_ | where *F*_obs_ and *F*_calc_ are the observed and calculated structure factors, respectively.

^c^ *R*_free_ is the sum extended over a subset of reflections (5%) excluded from all stages of the refinement.

^d^ As calculated using MolProbity.

^e^ Maximum-Likelihood Based Coordinate Error, as determined by REFMAC.

* Values in parentheses correspond to the highest resolution shell.

The structure of PgaA 220–342 is all α-helical and contains TPR-3 and TPR-4, as well as an unusually long α/α-motif containing 46 amino acids that likely represents α/α-5 based on our bioinformatics analysis (**Fig 3A and 3B**). Helix 1 of TPR-3 has 6 additional residues and is therefore slightly longer in length than a typical helix in a 34-residue canonical TPR motif. This helix also contains a kink, which allows it to tightly interact with the α/α-5 motif (**Fig 3B**). Longer TPR-like units containing more than 34 residues, such as the 42-peptide repeat (42PR) [17], have been observed previously. The closest structurally characterized homologue of TPR-3 and TPR-4 is the TPR-superhelix of *Homo sapiens* O-linked N-acetylglucosamine transferase (OGT) [18] with 14% sequence identity (**S4 Fig**). While, TPR-3 and TPR-4 display a very similar superhelical curvature to TPR domains found in OGT, the longer α/α-5 motif is not present in OGT. The α/α-5 motif differs noticeably from the common TPR fold and locally increases the curvature of PgaA (**S4 Fig**), thus forming a groove with conserved residues on the concave surface of the TPR-like module running parallel to the superhelical axis (**Fig 3C**, groove 1). This increased curvature also results in a groove running diagonal to the superhelical axis on the convex surface with conserved residues from the α/α-5 repeat lining the groove (**Fig 3C**, groove 2). Both grooves are predominantly neutral in charge, although the region with highest sequence conservation on the concave surface is slightly negatively charged (**Fig 3D**). We next tried to determine the co-structure of PgaA-220-342 with various substrates. Soaking and co-crystallization trials with PNAG and dPNAG oligomers failed to yield usable crystals. Analysis of the structure revealed that residues lining groove 1 in chains A and chain B are both involved in crystal packing (**S3B Fig**). The occlusion of the groove would prevent the substrates binding during soaking experiments, while the contacts required for crystal formation would unavailable due the presence of the bound oligomers in co-crystallization experiments. Overall, our structural analysis of PgaA-220-342 revealed a TPR-like module with two conserved grooves that could be potential interaction sites.

### PgaA’s TPR domain interacts with PNAG/dPNAG

**Fig 4:**
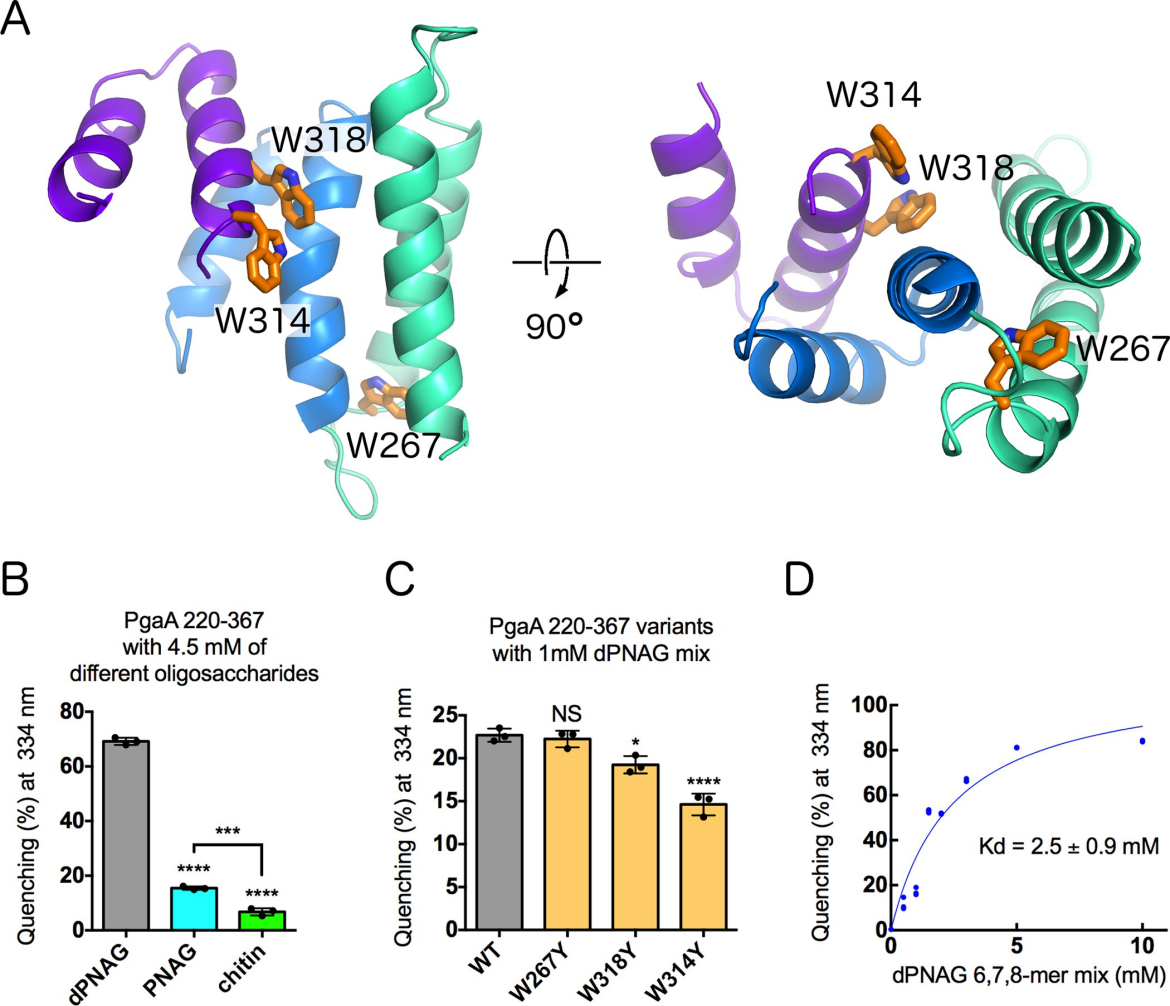
Tryptophan quenching suggests dPNAG interacts with the concave surface of the TPR. A) TPR 220–367 contains three tryptophan residues, shown in orange stick representation. Left is a view at the concave surface and right is a view along the superhelical axis. B) Tryptophan quenching of PgaA-220-367 with dPNAG, PNAG, and chitin oligomers. C) Tryptophan quenching with TPR variants suggests that dPNAG-interacts with residues W314 and W318. ****P ≤ 0.0001, ***P ≤ 0.001, *P ≤ 0.1, NS: no significant difference. Statistical significance was evaluated using two-way analysis of variance and Tukey’s multiple comparison test. Error bars represent the standard error (n = 3). D) Tryptophan quenching PgaA-220-367 wild-type as a function of dPNAG concentration. The dissociation constant and S.E. were obtained by fitting to single-site binding equation with nonlinear regression analysis, with R^2^ = 0.93 and a standard deviation of the residuals Sy.x = 8.3%. The analysis was performed with GraphPad Prism version 6.0 for Mac OS X.

Thus far, our data suggests that residues 220–515 of PgaA are involved in protein-protein interactions with N-terminal domain of PgaB. Examination of our PgaA TPR structure revealed three tryptophan residues, W267, W314 and W318. The latter two are solvent accessible and located near the conserved concave surface groove (**Fig 4A**). As TPR domains are known to coordinate more than one binding partner, and polysaccharide-binding sites frequently contain a mix of aromatic and polar residues, we next investigated whether PgaA-220-367 interacts with dPNAG and the role these tryptophan residues might play in this interaction. Using wild-type PgaA-220-367 we first determined the quenching capacity of a mixture of chemically synthesized, partially (50%) de-acetylated dPNAG oligomers of 6–8 residues (**S5 Fig**) and compared this to the quenching ability of fully acetylated PNAG hexamer and nonamer, as well as a chitin hexamer. At a substrate concentration of 4.5 mM we found that dPNAG, PNAG (9-mers and 6-mers), and chitin quenched the signal at 334nm by 69%, 18% (for both PNAG 9-mers and 6-mers), and 9%, respectively (**Fig 4B**), suggesting that dPNAG interacts with at least one of the three tryptophan residues. Fully acetylated PNAG also seems to interact with the tryptophan residues but appears to bind to a lesser extent or with lower affinity.

To probe how the tryptophan residues contribute to the observed quenching when dPNAG is used as a substrate, we generated three tryptophan mutants, W267Y, W314Y and W318Y. Circular dichroism (CD) experiments verified that the mutants were folded and stable (**S6 Fig**). At a substrate concentration of 1 mM the fluorescence of the wild-type, W267Y, W318Y and W314Y proteins was quenched by 22.7 ± 0.8%, 22.4 ± 1.0%, 19.3 ± 1.0%, and 14.6 ± 1.3%, respectively (**Fig 4C**). To determine the binding affinity of the protein for dPNAG, we next varied the substrate concentration between 0–10 mM and observed a dose dependent change in the quenching (**Fig 4D**). The K_d_ was calculated to be in the low millimolar range (2.5 ± 0.9 mM). Combined, these results suggest that dPNAG binds to the conserved groove on the concave TPR surface of residues 220–367 and that W314 and W318 are involved in this interaction.

Given the observation that dPNAG binds to the TPR module encompassing residues 220–367, we next sought to determine whether other regions of the TPR domain were able to bind PNAG and whether there was a preference for the fully acetylated or partially deacetylated polymer. We recently reported the successful synthesis of defined length mono-deacetylated PNAG oligomers [14] that can be produced in sufficient quantities for protein-ligand interaction studies by mass spectrometry. Therefore, we assessed the ability of various TPR constructs to bind a fully acetylated PNAG pentamer (GlcNAc)_5_ (mimicking PNAG) and a mono-deacetylated pentamer (GlcNAc)_2_-GlcN-(GlcNAc)_2_ (mimicking dPNAG) (**Table 2**). To cover the entire protein and taking into account the proteolytic degradation of PgaA-32-515 (**S1B, S1C and S1D Fig**), we initially generated 4 constructs encompassing residues 32–220, 32–367, 220–367 and 368–515. We found that constructs 32–220, 32–367 and 220–367 each produced soluble protein. Expression of the PgaA 368–515 construct, the region closest to the porin domain, resulted in insoluble protein. Variation of the boundaries of this construct (368–482, 352–464, 352–502, 401–502, 401–515) similarly resulted in either no expression or the protein being expressed in the insoluble fraction (**S7 Fig**). We were therefore unable to probe the binding of the polymer to this region of the protein. As a negative control for these studies, we used the human milk pentasaccharide lacto-N-fucopentaose (LNF1). LNF1 serves as basic control to check whether PgaA has affinity for a nonspecific sugar pentamer. We were unable to detect the binding of either the fully acetylated or mono-deacetylated oligosaccharides to residues 32–220 (**S8 and S9 Figs**), although it should be noted that mass spectrometry detected multiple species for this construct between 17,008 and 18,674 Da (expected molecular weight is 21,230 Da), which suggests that the protein was partially degraded which would complicate the analysis of any interaction that might have occurred. In contrast, residues 220–367 bound the pentasaccharides with Kd’s of 1.7 ± 0.3 mM and 1.3 ± 0.2 mM for (GlcNAc)_5_ and (GlcNAc)_2_-GlcN-(GlcNAc)_2_, respectively (**Table 2 and S10 and S11 Figs**). To further probe the role of residues 32–220, a longer construct encompassing residues 32–367 was generated and tested. For this construct we were able to calculate K_d_’s of 10 ± 5 mM and 2.3 ± 0.6 mM for (GlcNAc)_5_ and (GlcNAc)_2_-GlcN-(GlcNAc)_2_, respectively (**S12 and S13 Figs**). The lower affinities observed for both PNAG and dPNAG for the residues 32–367 relative to the shorter 220–367 construct suggest that either access to residues 220–367 is restricted or that the conformation of residues 220–367 is affected by residues 32–220. It is also worth noting that the affinity of dPNAG is slightly higher than for PNAG. These results are consistent with our tryptophan quenching data and reinforce the hypothesis that residues 220–367 interact with PNAG substrates. Our inability to detect PNAG/dPNAG binding to the N-terminal region, residues 32–220, suggests that the TPR module encompassing residues 220–367 might to the initial point of contact for the polymer.

**Table 2:** Mass Spectrometry analysis of PNAG/dPNAG binding to TPR Constructs.

TPR region	(GlcNAc)_5_ “PNAG”	(GlcNAc)_2_-GlcN-(GlcNAc)_2_ “dPNAG”	Fuc-Gal-GlcNAc-Gal-Glc (LNF1, control)^%^
32–220^#^	No binding	No binding	N.D.
32–367	K_d_* = 10 ± 5 mM	K_d_* = 2.3 ± 0.6 mM	No binding
220–367	K_d_* = 1.7 ± 0.3 mM	K_d_* = 1.3 ± 0.2 mM	No binding
368–515^$^	N.D.	N.D.	N.D.

^#^The sample of PgaA-32-220 consisted of at least 14 proteins with MWs ranging from 17,008 to 18,674 Da, which indicates the possibility of degradation for PgaA-32-220.

*Apparent association constants (K_d_) for PgaA proteins binding to PNAG and dPNAG at 25°C and pH 7 determined by a direct ESI-MS assay.

^$^The 368–515 C-terminal TPR construct could not be expressed in the soluble fraction.

^%^No specific binding was observed for pentasaccharide LNF1, after correction for nonspecific binding the fraction of bound LNF1 was less than 0.01.

N.D. Not determined.

Errors correspond to one standard deviation.

### Molecular dynamics simulations suggest dPNAG and PNAG bind to conserved residues on the concave surface of the TPR module

**Fig 5:**
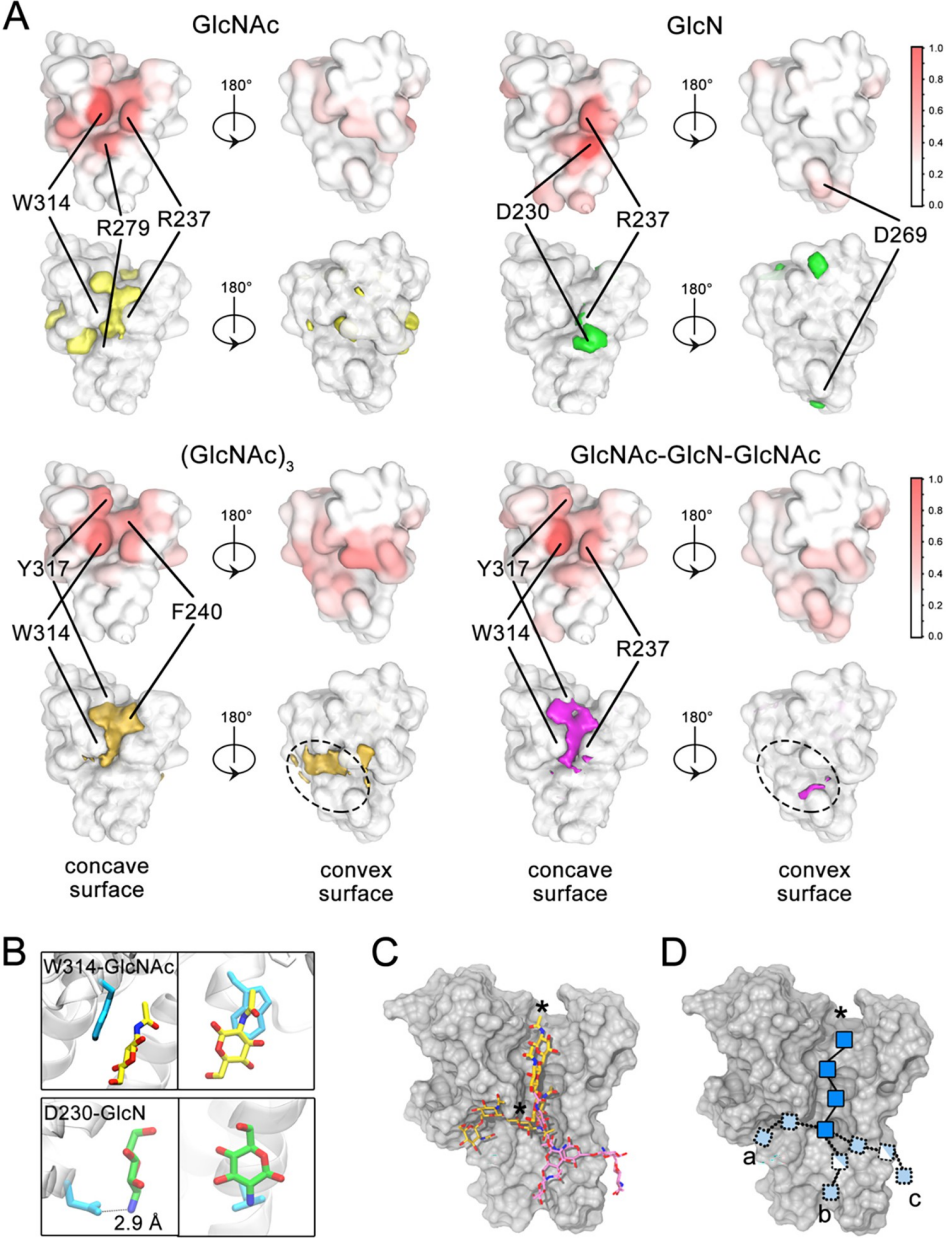
MD simulations reveal GlcNAc and GlcN binding sites along the concave TPR surface. A) Statistical analysis of MD simulations of PgaA 220–340 with 50 mM GlcNAc monomers, 50 mM GlcN monomers, 7 mM (GlcNAc)_3_ trimers, and 7 mM GlcNAc-GlcN-GlcNAc trimers. Two types of illustrations are used to show interaction regions: The upper illustration using a white-to-red color scheme indicates the fraction of time that a protein residue is bound to a sugar over the course of the simulation, while the lower illustration depicts the spatial density distribution for each bound sugar at 0.25 occupancy. B) Snapshots from MD simulations with monomers. C) Snapshot of MD simulations with trimers. D) Potential dPNAG pathway on PgaA 220–340 based on trimer binding orientations. The star indicates the reducing end of the polymer. The superhelical axis of the TPR domain is oriented vertically with the C-terminal end leading to the porin pointing upwards.

Given our observations that dPNAG binds to the TPR module, we next set out to determine the potential binding sites for PNAG and dPNAG. We have previously successfully employed molecular dynamics (MD) simulations to probe the binding of monosaccharides to PgaB [19], and adopted a similar approach to examine the binding of mono-, and trisaccharides to PgaA-220-340. We performed simulations on four ligands in total: GlcNAc, GlcN, (GlcNAc)_3_, and GlcNAc-GlcN-GlcNAc (**Fig 5**). To obtain statistically meaningful results, each simulation was repeated multiple times, with a combined simulation time of 120 μs (**S1 Table**). Consistent with our tryptophan quenching data (**Fig 4C),** each ligand, with the exception of GlcN, interacts most frequently with residue W314 (**Fig 5A**) which lines the groove on the concave surface of the TPR module. R237 is the second residue most frequently involved in interactions. This residue is located on the opposite side of the groove to W314. Additional residues in the groove that are frequently involved in interactions are R279 (especially with the GlcNAc monomer), F240 [(GlcNAc)_3_], and Y317 [(GlcNAc)_3_ and GlcNAc-GlcN-GlcNAc)]. GlcN interacts most frequently with D230, most likely due to the negative charge of this residue and the free amine on the sugar. The binding frequency analysis of our MD simulations suggests that the top half of the groove can bind both PNAG and dPNAG, and that these interactions occur predominantly with the conserved residues that line the groove. The simulations also suggest that the bottom half of the groove binds sugars with weaker affinity and/or a more heterogeneous ensemble of saccharide binding conformations (**Fig 5A**).

### MD analysis reveals distinct GlcN and GlcNAc binding modes

To understand the driving forces behind the dPNAG interaction with PgaA-220-340 we first analysed the binding modes of GlcNAc and GlcN, the building blocks of the polymer. As described above, GlcNAc and GlcN interact most frequently with W314 and D230, respectively (**Fig 5A and 5B)**. To better understand how the monomers interact with the protein and the relative orientations that the sugars could adopt, we defined planes for each sugar ring and the side-chains and computed for the GlcNAc-W314 interaction pair, the plane tilt angle ω_1_, the plane distance d_1_, and the plane rotation angle θ_1_; and for the GlcN-D230 interaction pair, the plane tilt angle ω_2_, the distance d_2_, and the plane rotation angle θ_2_ (**S14 Fig**). We observed a conformational basin (32 ± 9% occupancy) located at (ω_1,_ d_1_) = (20 ± 20°, 0.5 ± 0.1 nm), which suggests that the binding of GlcNAc to W314 involves a parallel stacking of the sugar ring on the tryptophan indole (**S15A and S15C Fig**). In this basin, most conformations have the reducing end pointing in the same direction (**S15C and S15E Fig**). In contrast, GlcN binds to D230 mainly via a salt-bridge (**Fig 5B**). A basin at (ω_2,_ d_2_) = (90 ± 50°, 0.35 ± 0.05 nm) with population of 41 ± 9% indicates binding perpendicular to the D230 sidechain (**Figs 5B and S16**). In summary, our analyses suggest that for GlcNAc the preferred binding mode is stacking with W314, whereas for GlcN electrostatic interactions with D230 seem to play the dominant role. The observed differences for the two dPNAG building blocks suggest that de-acetylation could affect the binding of polymer and might be used to differentiate between PNAG and dPNAG.

### MD trajectories for trimers are consistent with polymer translocation along the superhelical TPR axis

To assess whether the binding sites identified using monosaccharides are utilized by sugar units within a polymer and to gain insight into possible dPNAG secretion pathways, we next analyzed the orientation of (GlcNAc)_3_ and GlcNAc-GlcN-GlcNAc relative to W314 (**S17 and S18 Figs**) and GlcNAc-GlcN-GlcNAc relative to D230 (**S19 Fig**). Specifically, we computed the orientation of the middle sugar unit of each trimer, as we anticipate that this unit will better represent the conformation of sugars within a polymer. To gain information on the direction of trimer binding, we also analyzed the position of the reducing and non-reducing ends of the polymer (**S20 Fig**).

Using the analysis developed for the monosaccharides, we observed a stacking interaction of the central sugar with W314, with the trimer orientated parallel to groove 1 (defined in **Fig 3C**) of the TPR module (panel II in both **S17B and S18B Figs**). This pattern suggests that the most prevalent observed monomeric interaction with W314 is realized within a polymer. Our directional analysis indicates a preference for the reducing end pointing “upwards” towards the porin (**S20 Fig**), which is more pronounced for (GlcNAc)_3_ (62%) compared to GlcNAc-GlcN-GlcNAc (52%). We also observed binding patterns involving an electrostatic interaction of the charged central sugar unit with D230 (**S19B Fig**). Similar to the interaction of GlcNAc-GlcN-GlcNAc with W314, when interacting with D230 the trimer orientation with the reducing end pointing towards the porin is only marginally preferred (52%) (**S20C Fig**). Combining the observed modes of trimer binding (**S21 Fig**) suggests potential pathways that the dPNAG/PNAG polymer may follow along the TPR module. Our data suggest multiple possible binding modes at the bottom of the concave surface (**Fig 5C**), which would either allow a pathway from the lower N-terminal TPR region along the axis (pathway b in **Fig 5D**) or an entry from the side (pathways a and c in **Fig 5D**). The groove in the upper section of the module appears to be well suited to guide the polymer towards the porin, with a preference for the reducing end of the polymer pointing forwards.

### Full-length models of PgaA predict a pathway from the concave TPR surface to the outer-membrane pore

**Fig 6:**
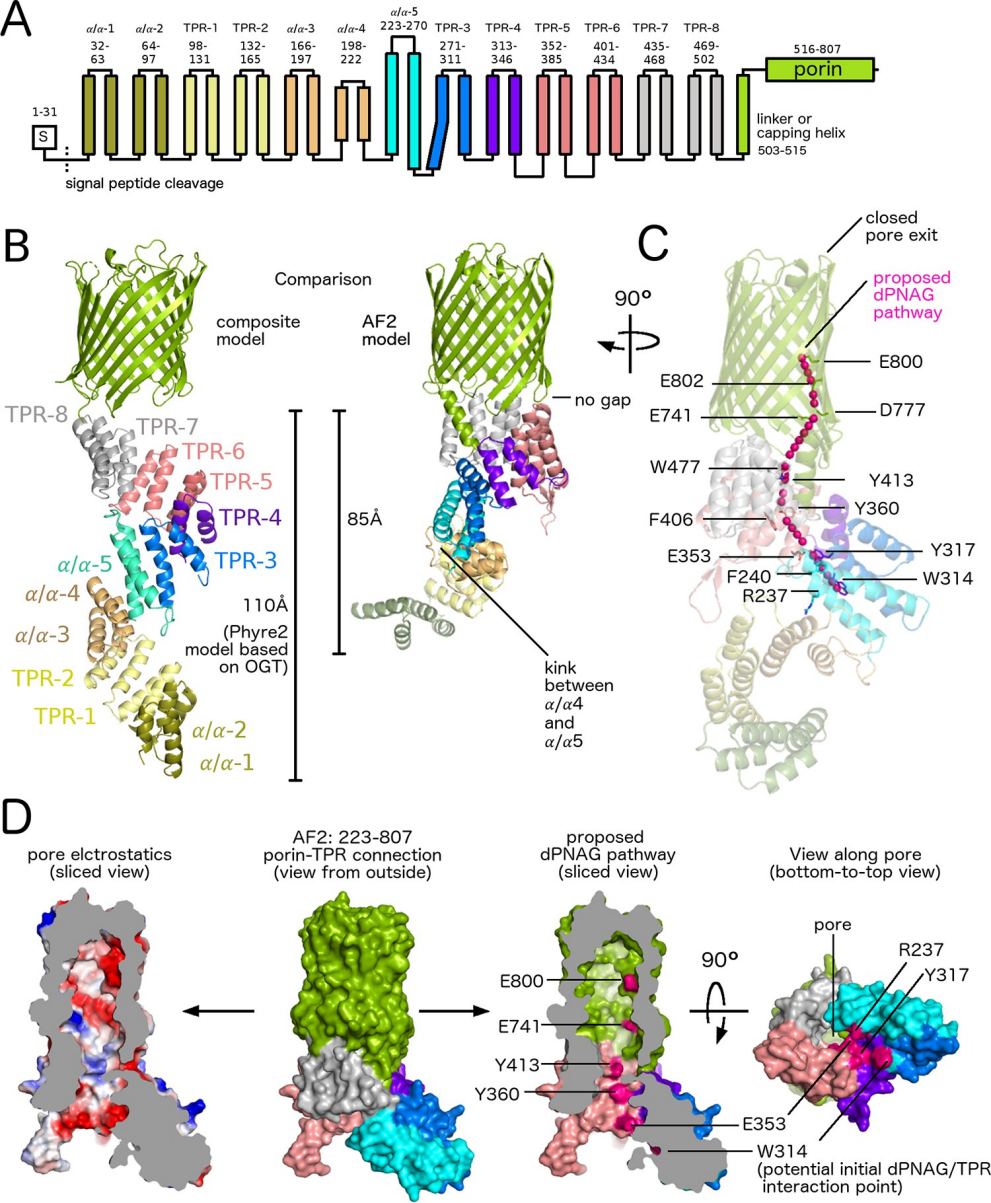
Homology and AlphaFold2 models of PgaA reveal a potential pathway for dPNAG into the outer-membrane porin. A. Topology model of PgaA based on structure prediction servers HHpred and TPRpred [22,23]. B. Left side: Composite model of PgaA. The TPR region is a Phyre2 model [21] based on human O-linked GlcNAc transferase (OGT, PDB ID 1W3B)[18] combined with the crystal structure of PgaA-220-367 (this work). The overall sequence identity of the TPR domain and OGT is only 14%. We expect the accuracy of the model to be higher in regions with a continuous TPR repeat (eg TPRs 5–8) since OGT shows continuous superhelical TPR fold. We cannot exclude the possibility of deviations from a continuous TPR superhelix, as for example seen in BcsC (PDB 5XW7) [4], which for PgaA seems more likely near the unclassified α/α-repeats. Right side: Alphafold2 (AF2) model of PgaA. C. Proposed dPNAG pathway shown together with a semi-transparent view of the AF2 model of PgaA. The proposed pathway runs along the labeled residues. Residues R237, W314, Y317 and F240 were selected based on the MD simulation in this work; residues E353, Y360, Y413 and W477 were selected based on their position on the concave TPR surface in the AF2 model; residues E741, D777, E800 and E802 were selected based on a complementation study with single-point mutants showing reduced biofilm formation [6]. D) Surface views of the AF2 model of PgaA-223-807.

The TPR domain of PgaA is the only part of the PgaA/B secretion complex that hasn’t been structurally characterized. With the exception of AlgK [3], only fragments of the TPR domains of PelB [5], BcsC [4,20], and PgaA (this work) have been crystallized. To gain better insight into structure-function relationships we generated full-length models of PgaA. First, we used our crystal structure in combination with Phyre^2^ [21] and HHpred [22,23] to generate a model of the TPR domain (**Fig 6A and 6B**). The result suggests the presence of 13 α/α-repeats, 8 of which have bona fide TPR consensus sequences (**Fig 6A**). The presence of the canonical TPR motifs predicts a superhelical fold that is closer to the general TPR-fold seen in OGT, than the wider Sel1-like repeat fold observed in AlgK [3] (**S22 Fig**). Next, we used the crystal structure of BcsC containing the porin and the terminal TPR repeat (PDB 6TZK) [20] to build the entire PgaA structure (**S23 Fig**). First, the terminal motif (TPR8) of our PgaA TPR model was aligned with the terminal motif (TPR19) of the BcsC porin structure. Then, the previously determined structure of the PgaA porin was aligned with the BcsC porin. Both porins are 16-stranded beta-barrels with a similar oval shape that align with an rmsd_Cα_ of 2.9 Å when most of the extracellular loops are ignored (**S24 Fig**). The resulting model of PgaA indicates that the porin and the TPR domain adopt a linear assembly, which would orientate the 110 Å long TPR domain perpendicular to the outer membrane (**Fig 6B**). During the preparation of this manuscript the structure prediction program Alphafold2 (AF2) [24,25] became available. We first compared the crystal structure of PgaA-224-342 to the AF2 model of PgaA and found a similar overall fold of both chain A (rmsd_Cα_ = 0.6 Å) and chain B (rmsd_Cα_ = 0.8 Å) (**S25A Fig**). We also compared our composite model to the full-length Alphafold2 (AF2) model (**Fig 6B**) and found two noticeable differences: (i) a kink between α/α-4 and α/α-5, reducing the length to 85 Å; and (ii) no gap between the upper TPR rim and the porin. The kink between α/α-4 and α/α-5, formed by residues 214–227, have a low predicted confidence level (**S25B Fig**), suggesting perhaps that this region could form a flexible hinge or might in fact be modeled incorrectly by AF2. In contrast, the residues involved in the interaction between the porin and the TPR domain are modelled with a high confidence level (**S25B Fig**), supporting the seamless connection between the TPR and the porin (**Fig 6D**). A closer inspection of TPRs 5–8 of both the homology and the AF2 model indicates that the concave TPR surface leads to the pore of the porin and is decorated with aromatic and acidic residues (shown in **Fig 6C and 6D** for the AF2 model), which would provide an optimal pathway for dPNAG to cross from the periplasmic TPR domain to the outer-membrane porin for secretion.

## Discussion

**Fig 7:**
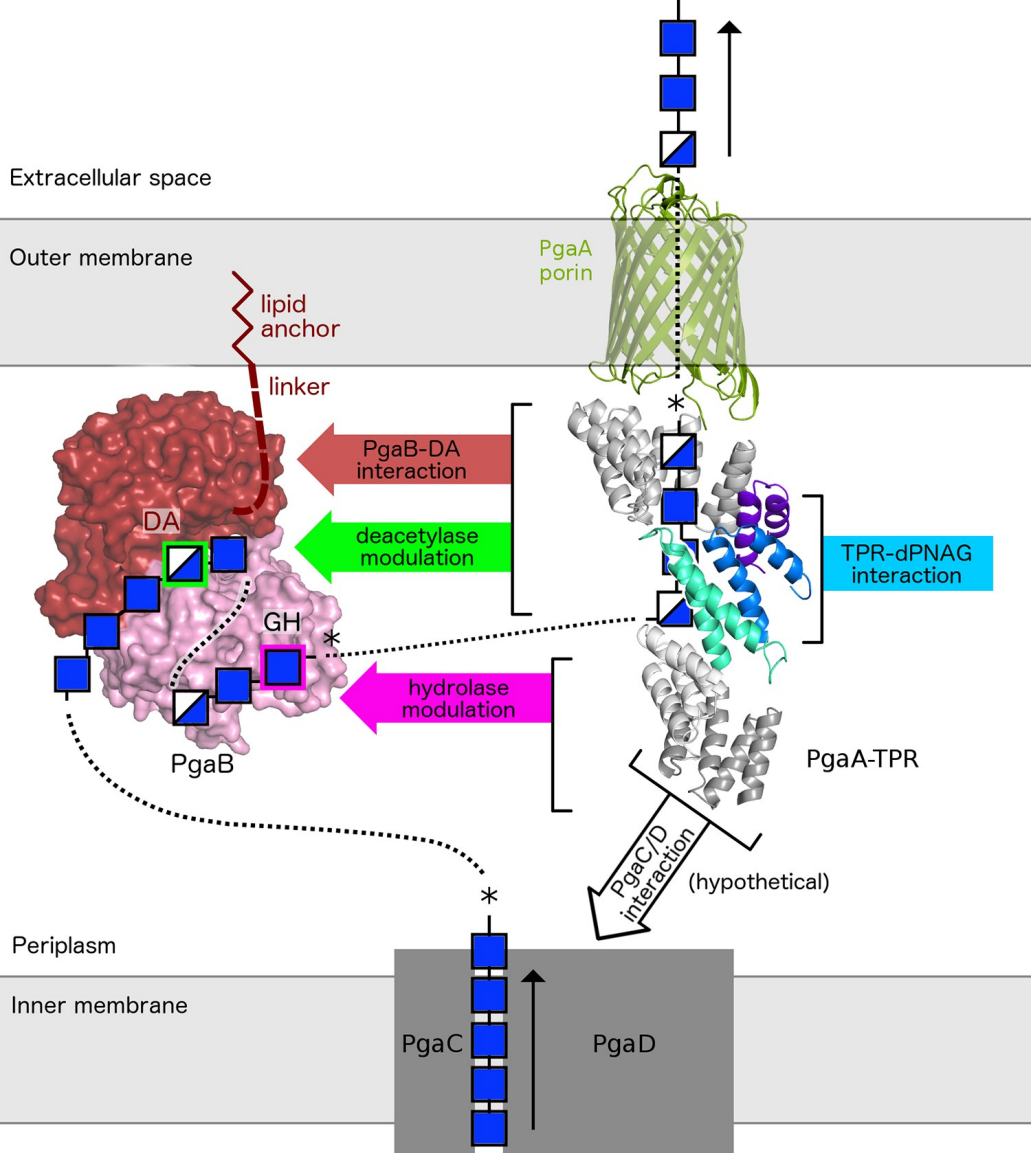
The TPR domain is a central scaffold in the PNAG processing and secretion mechanism. Summary of the functions of the TPR domain in PNAG processing and secretion. Colored signs/arrows indicate different TPR functions within the complex. The PNAG/dPNAG polymer likely runs continuously from synthesis to secretion as indicated by dotted lines. Where data supports, we have displayed the location of the polymer using a box, coloured using the standard nomenclature for GlcNAc and GlcN. The box sizes are not to scale. A star indicates the expected reducing end of the polymer. The direction of polymerization by PgaC/D is based on polymerization truncation experiments [32], which suggests polymer extension occurs at the non-reducing end. The direction of polymer being processed by PgaB-GH is based on a co-crystal structure of PagB and a PNAG hexamer (4P7R), and at the TPR domain it is based on MD simulation (this work). PgaB (using coordinates from PDB ID 4FD9) is shown in surface representation. The composite PgaA model (using coordinates for porin from PDB ID 4Y25) is shown in cartoon representation, with the crystallized TPR model (this work) is shown in green, blue, and purple.

In this study we report the characterization of the TPR domain of PgaA and show that this scaffolding protein plays three distinct roles in PNAG biosynthesis. Our data support roles for the TPR domain in the formation of a secretion complex via its interaction with PgaB; the modulation of PgaB’s de-acetylase and glycoside hydrolase activities, which has implications for the percent deacetylation and length of the secreted polymer; and the binding of dPNAG polymer and thus a role in polymer export. While the exact pathway for PNAG/dPNAG crossing the periplasm is still unknown, our current results allow us to predict a potential mechanism for periplasmic PNAG processing (**Fig 7**). After PNAG is produced in the inner membrane it has to crossover to the PgaA/B secretion complex. Our homology model predicts that the TPR domain extends perpendicular from the outer membrane 110 Å into the periplasm, and 85 Å in the AF2 model due to the kink between α/α-4 and α/α-5 (**Fig 6B**). The periplasmic space in *E*. *coli* K12 has been reported to be as small as 106Å [26], suggesting that the TPR might bridge PgaC/D and PgaA/B. While, there is currently no experimental evidence available that these protein complexes are physically linked in the PNAG system, an interaction between the TPR domain containing AlgK and the inner membrane c-di-GMP receptor Alg44 has been observed in the alginate secretion system [27]. We hypothesize that the N-terminal region of PgaA, residues 33–220, would be involved in this interaction, and further given our data demonstrating that PgaA interacts with PNAG/dPNAG that production and secretion of polymer would help stabilize a PagABCD complex. Further experimentation is necessary to determine the exact nature of the interaction between the PgaC/D and PgaA/B complexes and how to polymer bridges these complexes.

Once in contact with the PgaA/B complex, the polymer needs to interact with PgaB (**Fig 7**), as partial deacetylation of the polymer is required for secretion and biofilm formation [9]. We have previously predicted using MD simulations of PgaB with GlcNAc and GlcN that the polymer might wrap around PgaB from the N-terminal deacetylase to the C-terminal endo-acting glycoside hydrolase domain [11,19] (**Fig 7**). The notion that the polymer first interacts and is partially deacetylated prior to its interaction with the glycoside hydrolase domain of PgaB is also supported by characterization of the glycoside hydrolase domain that found that the enzyme can cleave dPNAG but not PNAG [11]. After interacting with PgaB, the partially deacetylated polymer would then interact with the TPR domain of PgaA and cross the outer membrane via the porin. Eventually, the polymer would span from PgaC/D via PgaA/B to the extracellular space (**Fig 7**), at which point it could be cleaved by the glycoside hydrolase domain of PgaB. The frequency of cleavage of the translocating polymer might dictate the chain-length of the secreted dPNAG, which might subsequently affect biofilm characteristics. Both cell-associated and cell-free forms of dPNAG have been observed in *E*. *coli* K-12 [28]. Our interaction data (**Fig 1C**) coupled with our activity assays (**Fig 2B**) presented herein suggest that the TPR region PgaA-221-515, proximal to the porin, interacts with the deacetylase domain of PgaB, and that this interaction increases deacetylase activity 2.3-fold. In the Pel system a comparable 1.7-fold increase in deacetylase activity was reported upon complex formation [5]. This activation by the TPR domain might ensure that partially deacetylated PNAG, which can be thought of as the “activated glue” for biofilm formation, is only generated if it can be readily transported out of the cell via the TPR and the porin. We also detected a 13-fold increase (from EC_50_ = 302.7 ± 82.3 nM alone to EC_50_ = 22.8 ± 5.1 nM in complex) in glycoside hydrolase activity (**Fig 2C**) when PgaA and PgaB interact. In contrast, in the Pel system a 4.2-fold decrease in hydrolase activity (from EC_50_ = 30.0 ± 1.4 nM alone to EC_50_ = 125.3 ± 4.2 nM in complex) was reported [5]. Our enzymatic assays suggest that the N-terminal TPR region PgaA-32-220 is involved in modulating the activity of the PgaB glycoside hydrolase domain, suggesting that there could be a physical interaction between both domains (**Fig 7**). The hinge between α/α-4 and α/α-5 in the TPR domain suggested by the AF2 model might provide some flexibility in this region and enable it to interact with both the PgaC/D complex and PgaB’s hydrolase domain. While the exact biological role of glycoside hydrolases in synthase-dependent exopolysaccharide biosynthesis is still under investigation, it has been recently reported that deletion of the glycoside hydrolase BCE_5582 in *B*. *cereus* results in a thicker biofilm [29], suggesting that glycoside hydrolases might play a role in altering the characteristics of the biofilm, for example by affecting the polymer chain length and/or the ratio of cell-associated to cell-free polymer. In addition AlgL, the lyase in the alginate system, has recently been shown to be involved in homeostasis, responsible preventing cell lysis by clearing the periplasm of any accumulated alginate polymer [30]. The biological function of hydrolase BcsZ in the cellulose biosynthetic system is unclear, but is has also has hypothesized that it might prevent periplasmic polymer accumulation and/or influence the formation of cellulose microfibrils in the extracellular space by cleaving translocating polymer [31]. Taken together, these data suggest that glycoside hydrolase domain of PgaB might also have multiple functions.

In the ABC transporter and Wzx/Wzy-dependent exopolysaccharide systems the polysaccharide copolymerase (PCP) and outer-membrane polysaccharide export (OPX) protein form a tube-like channel to bridge the periplasmic gap [1]. The TPR domain in synthase dependent systems is unlikely to form an enclosed channel or pore based on its superhelical fold. While this may seem to be a mechanical disadvantage, it might be an elegant mechanism to allow the participation of a variable number of enzymes that can chemically alter the polymer before secretion. The repetitive modular character of TPR domains might also reflect an evolutionary strategy to optimize the processing of different exopolysaccharides by recruiting pre-existing enzymes, this strategy of “rewiring” pre-existing components during evolution has been suggested for other scaffolding proteins [13]. In fact, the operons across the different synthase-dependent systems vary significantly in the number of periplasmic processing enzymes. The alginate system has a total of five periplasmic enzymes (three acetylating enzymes AlgJ/F/X, one epimerase AlgG and one lyase AlgL, whereas the Pel and PNAG systems have only one large dual-functional deacetylase/glycoside hydrolase enzyme, PelA and PgaB, respectively. Similarly, the cellulose producing system contains one periplasmic enzyme (BcsZ), while the acetylated cellulose system contains four (WssD/F/G/I) [1]. The geometry of the TPR fold seems well suited to guide a polymer towards the porin for export via its concave surface, suggesting that perhaps the convex surface binds and modulates the activity of polymer processing enzymes. The TPR domain would thus also have the role of an assembly-line type scaffold, facilitating the transfer of the polymer to different proteins/domains in the pathway. Demonstrating that the polymer binds to the TPR is challenging due to substrate availability/solubility and because the interaction is likely not tight so as to allow movement along the protein for secretion. We have overcome some of these limitations by using short synthetic oligosaccharides and used three different methods to demonstrate that the TPR module formed by TPR-3/4 and α/α-5 (**Figs 3 and 6B**) binds to PNAG. Tryptophan quenching and MD simulations both indicated binding to residues on the concave TPR surface, and suggest that W314 is a critical residue involved in the interactions. Both tryptophan quenching and mass spectrometry analysis indicated a preference for binding of dPNAG over PNAG, which suggest this binding site might be used for export (see discussion about the role of deacetylation above). Binding affinities in the low mM range are common for protein-polysaccharide interaction, for example the K_d_ for PNAG oligomer to PgaB has been reported to be 1.3 ± 0.2 mM [19]. Despite this low binding affinity, the polymeric nature of the polymer with multivalent interactions will likely prevent a complete loss of interaction with the protein, meaning that some parts of the polymer might stay bound while others can shift in order to allow forward motion. The MD simulation with PNAG and dPNAG trimers revealed a subset of binding conformations parallel to the superhelical TPR axis along the concave surface, a binding mode that would be compatible with the binding of dPNAG to a continuous stretch of TPR residues (**Fig 7**). Related to this last observation, our homology model of neighboring TPRs 5–8 predicts that the concave surface leading towards the porin contains many acidic and aromatic residues (**Fig 6C and 6D**), potentially allowing this region to funnel dPNAG towards the porin (**Fig 7**). A directional analysis of simulated trimers revealed a preference for the reducing end pointing towards the porin, which would be a consistent with PNAG elongation by the synthase PgaC/D occurring form the non-reducing terminus (i.e. adding sugar units to the rear non-reducing end of the polymer while the reducing end travels forwards), a mechanism that we recently proposed based on polymerization truncation experiments [32].

Our study suggests that the TPR domain is divided into modules with different functionalities: binding to PgaB, modulation of PgaB deacetylase activity, modulation of PgaB glycoside hydrolase activity, and dPNAG/PNAG binding (**Fig 7**). The ability to bind both product (dPNAG) together with its modifying enzyme (PgaB) suggests that the TPR domain of PgaA acts as a scaffolding domain. Modulation of both the deacetylase and glycoside hydrolase activity could be used to control deacetylation levels as well as the cleavage rate of the secreted dPNAG. An example for a TPR-scaffold protein is the well-studied “HSP70 and HSP90 organizing protein” (HOP), which links chaperones HSP70 and HSP90 and modulates their activities [33]. Scaffold proteins are known as key regulators in complex pathways [12], and recognizing the TPR domain of PgaA as such raises the intriguing question of whether it could have a regulatory role within the pathway of synthase-dependent exopolysaccharide secretion. While the production of polymer by the synthase is regulated by intracellular cyclic-di-GMP, the periplasmic secretion complex might be a second regulatory system to fine-tune biofilm characteristics or to provide feedback from the downstream processing and secretion processes. Understanding the underlying molecular and evolutionary mechanisms of biofilm-related exopolysaccharide production is an important task in the fight against chronic infections and antibiotic resistance.

## Materials and methods

### Cloning, expression and protein purification for interaction studies and enzymatic assays

*E*. *coli* K-12 *pgaA* residues 32–807 was subcloned into the pET28 vector using inverse PCR, restriction digest, and ligation cloning (NdeI and HindIII sites) using pPGA372 (*pgaABCD* operon on pUC19) as the template. This produced the expression plasmids pET28-PgaA which encodes PgaA 32–807 with an N-terminal thrombin cleavable His6-tag. PgaA residues 32–807 was then subsequently subcloned into the vector pETDL (pET28 MCS cloned into the pET26b vector that has the endogenous NdeI site mutated) using restriction digest and ligation cloning (NdeI and HindIII sites). This produced the expression plasmid pETDL-PgaA 32–807 which encodes PgaA with an N-terminal *pelB* leader sequence followed by a thrombin cleavable His6-tag.

For PgaA/B complexes (His-PgaA/PgaB, His-PgaA-Δ514/PgaB, His-PgaA-32-515/PgaB, His-PgaA/PgaB-D115A, His-PgaA/PgaB-DA, His-PgaA-Δ220/PgaB, His-PgaA-Δ220/PgaB-C21S) the pETDL-PgaA-32-807 plasmid was used as a template to amplify *pgaA*, residues 32–807 with the N-terminal pelB leader and His6-tag. This sequence was subcloned into the pQLinkN vector using restriction digest and ligation cloning (BamHI and NotI sites). *pgaB* residues 1–672 were amplified from Topo 2.1-PgaB (an existing Topo 2.1 plasmid with the *pgaB* genomic sequence) and subcloned into pQLinkN. The two plasmids were combined to generate the co-expression vector pQLinkN-*his*-*pgaA/pgab* [6,34]. Mutations and deletions were performed on the parent pQLinkN plasmids which were subsequently combined to generate all the constructs for the pulldown experiments, glycoside hydrolase and deacetylase assays.

For expression of membrane proteins (PgaA and PgaA/B complexes) BL21 cells were transformed with the appropriate co-expression plasmids. A single colony was used to inoculate 5 mL of LB-Amp_100_ in a 12 mL plastic culture tubes and grown overnight at 37°C with shaking (200 rpm). The overnight cultures were used to inoculate 1L of LB-Amp_100_, which were then grown to an OD_600_ = 0.6. The cultures were induced with 1 mM IPTG (final) and incubated over night at 18°C. Cells were harvested by centrifugation at 5,000*g* for 15 min and re-suspended in 40 mL of 1X phosphate buffered saline (1X PBS, 11.8 mM total phosphate concentration, pH 7.4) with protease inhibitors. Cells were lysed by homogenization through an Emulsiflex-c3 (4 passes, 10-15K psi) or sonication (Misonix 3000) on an ice bath at 65% output for 1 min and 30 sec. The lysate was centrifuged at 40,000*g* for 30 min at 4°C. The supernatants were discarded, and pellets resuspended in 20 mL of 1X PBS supplemented with 1% (w/v) n-dodecyl-β-d-maltoside (DDM) based on a previously published protocol [6]. Cellular debris and membranes were removed by centrifugation at 40,000*g*. The supernatant was flowed through a gravity column packed with 4 mL NiNTA resin and pre-equilibrated with 1X PBS supplemented with 0.5% (w/v) DDM. The column was washed with 5 column volumes of 1X PBS with 0.2% (w/v) DDM and 20 mM imidazole and then eluted with elution buffer (20 mM Tris pH 8, 150 mM NaCl, 260 mM imidazole, 0.1% w/v DDM). The elution fraction (~10 mL) was concentrated to 2 mL and injected into a HLS200 gel filtration column pre-equilibrated with SE buffer (20 mM Tris pH 8, 150 mM NaCl, 0.1% (v/w) DDM).

Soluble constructs without N-terminal *pelB* leader sequence (PgaA-32-515, PgaA-352-502, PgaA-368-482, PgaA-368-515, PgaA-352-464, PgaA-401-515, PgaA-32-367, PgaA-32-220, PgaA-32-367, PgaA220-367) were created using restriction digest and ligation cloning (NdeI and HindIII sites). Point mutants of PgaA-220-367 (W267Y, W314Y and W318Y) were created with the QuikChange Lightening Mutagenesis kit by using plasmid pET28-PgaA-220-367 as template. All soluble proteins were expressed as described below for the native PgaA-32-367 for crystallization trials. Solubility of PgaA-32-515, PgaA-352-502, PgaA-368-482, PgaA-368-515, PgaA-352-464 and PgaA-401-515 was tested in various buffer conditions. Lysis and nickel purification of PgaA-32-220 was performed in buffer containing 50 mM MES pH 6, 300 mM NaCl and 5% (v/v) glycerol. Nickel purification was performed similar to native Pga-32-367 and is described below. Mass spectrometry of PgaA-32-515 was performed by SPARC BioCentre (Molecular Analysis), The Hospital or Sick Children, Toronto, Canada.

The cloning, expression and purification protocol of PgaB-22-672 has been published previously [10]. Strains, plasmids, and primers used in this study are summarized in **S2 Table**.

### Pull down experiments and western blotting

To test interactions between *pgaA* and *pgaB* constructs, BL21 cells were transformed with the appropriate co-expression plasmids. A single colony was used to inoculate 5 mL of LB-Amp_100_ in a 12 mL plastic culture tubes and grown overnight at 37°C with shaking (200 rpm). 2.5 mL of overnight cultures were used to inoculate 50 mL of LB-Amp_100_, which were then grown to an OD_600_ = 0.6. The cultures were induced with 1 mM IPTG (final) and incubated over night at 18°C. Cells were harvested by centrifugation at 5,000*g* for 15 min, re-suspended in 2 mL of 1X phosphate buffered saline (1X PBS) with protease inhibitors and sonicated (Fisher Scientific Sonic Dismembrator Model 100) on an ice bath at 40% output for 1 min. The lysate was centrifuged at 21000*g* for 30 min at 4°C. The supernatants were discarded, and pellets resuspended in 2 mL of 1X PBS supplemented with 1% w/v DDM. Cellular debris and membranes were removed by centrifugation at 21000*g* and the supernatants were subjected to Ni-NTA batch purification. Briefly, 100 μl of NiNTA resin was added to 1.5 mL Eppendorf tubes. Resins were pre-equilibrated with 1X PBS supplemented with 0.5% DDM. Supernatants were added and incubated, rotating for 30 min at 4°C. The samples were spun down at 21000*g* at 4°C. Supernatants were discarded and the resin was washed 3 times with 10 bed volumes of 1X PBS supplemented with 0.5% DDM. Proteins were eluted with elution buffer.

15 μl of Ni-NTA elutions were combined with 15 μl of 2X SDS-loading buffer (100 mM Tris-Cl pH 6.8, 4% (w/v) sodium dodecyl sulfate, 0.2% (w/v) bromophenol blue, 20% (v/v) glycerol, 200 mM β-mercaptoethanol), heated to 95°C and subjected to electrophoresis (35 min, 280 V, 50 mA, 10 W). Following electrophoresis, proteins were transferred onto a PVDF membrane and blocked with PBS-T buffer (1X PBS, 0.05% Tween-20) containing 5% w/v skim milk. The membrane was incubated with either a mouse monoclonal anti-his (1:1500) (Abgent) or a rabbit polyclonal primary antibody (1:2500) against PgaB, rocking for 1 hr at room temperature. Membranes were then washed 3X with 15 mL of PBS-T and further incubated with a horseradish peroxidase conjugated goat anti-Mouse (PgaA) or a goat-anti rabbit secondary antibody (PgaB) for 1 hr. Membranes were washed 3X with 15 mL PBS-T and exposed using the SuperSignal West Pico PLUS chemiluminescent substrate (Thermo Scientific).

### Fluorescamine de-acetylation assay

Each sample was prepared in triplicate to a final volume of 10 μl in 50 mM HEPES, pH 7.5, 10 μM nickel chloride with 10 μM enzyme and 10 mM PNAG hexamer and incubated for 24 hr at 20°C. To correct for the presence of free amines on the enzyme, each tested enzyme was added to control samples at a final concentration of 10 μM just prior to reaction with fluorescamine. The reaction was performed by adding to each 10 μl sample, 20 μl of 0.5 M borate buffer, pH 9.0, and 10 μl of a freshly prepared 20 mg/ml fluorescamine solution in dimethylformamide and mixed by pipetting. The reaction was then allowed to stand at room temperature for 10 min before adding 80 μl of deionized water. An 80 μl aliquot of the solution was removed from each sample and transferred to a Corning 3686 half-area 96-well plate for measurement in a Synergy Neo2 plate reader (360-nm excitation, 460-nm emission, 5-nm slit widths). Glucosamine solutions were used as standards to calculate amine concentration.

### Glycoside hydrolase disruption assay

Cultures of *S*. *epidermidis* clinical isolate SE801 were grown overnight at 37°C with shaking at 200 rpm in LB broth without antibiotics. The next day cultures were sub-cultured 1:100 into tryptic soy broth (TSB), mixed thoroughly, and 100 μl was added to individual wells of a sterile Cellbind surface plate (Corning) and the plates were incubated statically for 24 h at 37°C to allow for biofilm formation. To eliminate edge effects, 200 μl of sterile water was placed in all outside wells and the plate was sealed with parafilm. After incubation non-adherent cells and media were removed by washing the plate with deionized water three times. The wells were filled with 95 μl of 100 mM HEPES buffer pH 7.0 followed by 5 μl of varying concentrations of protein. Reactions were incubated for 2 h at 20°C on a rotating nutator at which time the reaction was quenched by washing the plates with deionized water three times. The wells were then stained with 150 μl of 0.1% (w/v) crystal violet for 10 min and washed again three times with deionized water. The remaining dye was solubilized with 100 μl of 95% (v/v) ethanol for 10 min with rotation, after which time the absorbance was measured at 595 nm.

### Cloning, expression and protein purification of PgaA-32-367 for crystallization

The introduction of a stop codon into plasmid pET28-PgaA 32–807 (see above) at residue 368 was used to produce plasmid pET28-PgaA32-367 with the QuikChange Lightening Mutagenesis kit. The resulting plasmid was transformed into BL21 CodonPlus for native PgaA-32-367 protein and B834 for SeMet-PgaA-32-367 protein. A single colony was used to inoculate 50 or 100 mL of LB-Kan_50_ for native and SeMet PgaA-32-367, respectively, in an Erlenmeyer flask and grown overnight at 37°C with shaking (200 rpm). For native PgaA-32-367 the 50 mL of overnight culture was used to inoculate 2 L of LB-Kan_50_; for SeMet-PgaA-32-367 the 100 mL overnight culture was harvested by centrifugation to remove the LB media and re-suspended in 2 L of M9 minimal media-Kan_50_ supplemented with 4 ml 1 M MgSO_4_, 20 ml 40% w/v glucose, 4 mL 12.5 mg/mL FeSO_4_, 2 mL 1 mg/mL vitamin mix (riboflavin, niacinamide, pyridoxine monohydrodrochloride, thiamine), 20 mL 4 mg/mL amino acid mix 1 (no trp/tyr/phe/met), 20 mL 4 mg/mL amino acid mix 2 (trp/tyr/phe) pH 8.0, and 8 mL 40 mg/mL seleno-methionine. Both native and SeMet protein cultures were grown to an OD_600_ = 0.4–0.5 and then moved to 18°C for 20–30 min until the OD_600_ = 0.6–0.7 and then induced with 1 mM IPTG (final) and incubated overnight. Cells were harvest by centrifugation at 5,000*g* for 15 min, re-suspended in 40–50 mL of TPR lysis buffer (50 mM Tris pH 7.5, 300 mM NaCl, 10 mM imidazole, 5% v/v glycerol, and a protease tablet), and lysed by homogenization through an Emulsiflex-c3 (4 passes, 10-15K psi). Cellular debris was removed by centrifugation at 30,000*g* for 30 min. The supernatant was flowed through a gravity column packed with 4 mL NiNTA resin and pre-equilibrated with TPR wash buffer (20 mM Tris pH 8, 300 mM NaCl, 5% v/v glycerol, and 10 mM imidazole pH 8). The column was washed with 5 column volumes PgaA wash buffer, 2 column volumes of TPR wash buffer with 20 mM imidazole, and then eluted with TPR wash buffer supplemented with 250 mM imidazole pH 8. The elution fraction (~10 mL) was then dialyzed in 1 L TPR buffer (20 mM Tris pH 7.5, 300 mM NaCl, 5% v/v glycerol) overnight at 4°C. To remove the histidine tag, the protein was incubated with 1 μL of restriction grade thrombin (Novagen) to 4 mg of protein at room temperature for 60–120 min. The solution was then subjected to a second round of Ni purification, where the flow-through and wash fractions were run on a gel to identify where the untagged protein eluted. The fractions containing the untagged protein (flow-through and wash 1) were concentrated to 2 mL and injected into a HLS200 gel filtration column pre-equilibrated with TPR SE buffer (20 mM Tris pH 8, 150 mM NaCl, 5% v/v glycerol). Strains, plasmids, and primers are summarized in **S2 Table**.

### Crystallization and crystal structure determination

PgaA-32-367 (His-6 tagged and untagged) was subjected to crystallization trials using sitting drop vapour diffusion at 10–15 mg/mL using the MCSG-1 to MCSG-4 screens and a Crystal Gryphon drop setter (Art Robbins Instruments). Initial crystals were observed in condition MCSG-1 #23 (20% w/v PEG 8000, 0.1 M Tris pH 8.5, and 200 mM MgCl_2_) after 2–3 weeks. Optimized crystals of native PgaA-32-367 and SeMet-PgaA-32-367 were obtained without the His6-tag, at a concentration of 16 mg/mL in: (1) 15% w/v PEG 8000, 0.1 M Tris pH 7.1, 200 mM MgCl_2_, and 3% w/v cadaverine; and (2) 10% w/v PEG 8000, 0.1 M Tris pH 7.6, 200 mM MgCl_2_, and 3% w/v xylitol, respectively. Crystals were harvested and cryo-protected with well solution including 20% v/v ethylene glycol before vitrification. Native and Se-SAD diffraction data were collected at beamline X29 NSLS with 360° of data at 1° ϕ oscillation. The data were indexed, integrated, scaled, and mergedusing HKL2000 [35]. SHELXD and SHELXE [36] were used to determine 3 selenium sites in the asymmetric unit and to calculate density-modified phases, respectively. Anomalous density around the selenium sites helped to find the correct register of the similar looking TPR motifs. The Se-sites were used as initial reference points and each residue was added manually in Coot. Since the crystals diffracted anisotropically, we truncated data outside an ellipsoid intersecting with the three principal axes (along 1/*a*, 1/*b*, 1/*c*) at 3.00, 3.00, and 2.85 Å, respectively and scaled the remaining data using the California at Los Angeles (UCLA) diffraction anisotropy server [37]. This reduced the completeness in the higher resolution shells but increased the overall *I*/σ*(I*) value of the remaining data (see **Table 1** for details) and improved features in the electron density. During alternating manual model building in Coot [38] and refinement with REFMAC[39] we noticed that PgaA-32-367 had apparently fragmented and the asymmetric unit contained only two copies of the C-terminal fragment. Chain A and B were modeled from residues 224–359 and 220–343, respectively. The final model was refined isotropically with non-crystallographic symmetry restraints between chain A and B and Translation-Libration-Screw (TLS) constraints (using one TLS group each for chains A and B) against anisotropically scaled native data with resolution cut-off between 2.85 and 3.00 Å resulting in R_work_/R_free_ of 23.5%/26.4%. Data and refinement statistics are summarized in **Table 1.**

### Synthesis of PNAG and dPNAG compounds

PNAG and dPNAG oligomers: PNAG pentamers (for mass spectrometry analysis) and PNAG hexamers (for fluorescamine assay) were synthesized as outlined previously [9,40]. A mix of dPNAG oligomers (6-,7-,8-mers) with a deacetylation level of approximately 50% and a random distribution of deacetylated sites was used for our tryptophan quenching assay. In this case the average molecular weight was used for calculation of concentrations. The synthesis of a defined dPNAG pentamer (GlcNAc)_2_-GlcN-(GlcNAc)_2_ (for mass spectrometry analysis) was performed as published previously[14].

### Tryptophan quenching

Fluorescence measurements were carried out at 20°C in a quartz cuvette (type no. 115F-QS; Hellma Analytics) using a PTI QuantaMaster 80 steady-state fluorometer (Photon Technology International), with a 4-nm bandwidth for both excitation and emission and a speed of 2 nm/s. Fluorescence spectra were collected between 300 nm and 400 nm with an excitation wavelength of 280 nm and a peak emission wavelength of 334 nm. All samples were prepared in triplicates 10 minutes ahead of the collection in a solution containing 1 μM protein, 20 mM Tris buffer pH 8.0, 150 mM sodium chloride and 1 mM concentrations of dPNAG oligomers. Data measured for each sample was corrected for ligand fluorescence and inner filter effect. All ligands had linear fluorescence over the concentration range used in this study and did not exceed 5% of PgaA-220-367 fluorescence. To obtain an approximate binding affinity the dPNAG oligomer concentration was varied between 0–10 mM. Protein folding and stability were assessed by performing CD scans from 200–300 nm and temperature melts at 222 nm using a Jasco J-1500 Circular Dichroism instrument.

### Mass spectrometry analysis

The dissociation constants (K_d_) for PgaA proteins (P) with PNAG and dPNAG ligands (L) interactions were measured in 200 mM aqueous ammonium acetate (pH 6.8) using the direct electrospray ionization mass spectrometry (ESI-MS) assay. Solutions of PgaA220-367 (5.7 μM) and PgaA32-367 (11.6 μM) were used for binding assay. The PNAG initial concentrations varied from 23 to 90 μM, and the dPNAG initial concentrations varied from 30 to 150 μM. Binding measurements were carried out in positive ion mode using a Synapt G2S ESI-Q-IMS-TOF mass spectrometer (Waters, Manchester, UK) equipped with nanoflow ESI (nanoESI) source. NanoESI was performed by applying a voltage of ~1 kV to a platinum wire inserted into the nanoESI tip, which was produced from a borosilicate glass capillary (1.0 mm o.d., 0.68 mm i.d.) pulled to ~5 μm o.d. using a P−1000 micropipette puller (Sutter Instruments, Novato, CA). The source temperature and gas flow rate was 60°C and 2 mL/min, respectively. The Cone, Trap and Transfer voltages were 20 V, 3 V and 1 V, respectively. MassLynx software (version 4.1) was used for data acquisition and processing.

The K_d_ for protein (P)—ligand (L) binding was calculated from the abundance ratio of L-bound to free P ions (i.e., *R*), measured by ESI-MS: *R* = *Ab*(PL)/*Ab*(P) = [PL]/[P], K_d_ = [P][L]/[PL] = [L]_0_/*R—*[*P*]_0_/(*R*+1), where [P]_0_ and [L]_0_ are initial concentrations of protein and ligand, correspondingly [41]. Non-specific protein-ligand binding was corrected as published [42].

PgaA32-220 was analyzed using the SolariX 15 T FT-ICR mass spectrometer (Bruker-Daltonics, Billerica, MA), equipped with a dynamically harmonized ParaCell and nanoESI source. Analysis of the PgaA32-220 sample revealed at least 14 proteins with MWs ranging from 17,008 to 18,674 Da, which indicated the possibility of its degradation. Interactions of PgaA32-220 to PNAG and dPNAG were tested and showed no binding. The instrumental parameters for these measurements were: glass capillary exit voltage (200 V); source drying gas (N_2_, 4 L/min and 120°C); deflector plate (220 V). The RF amplitude of the ion-funnels was 300 V peak-to-peak, and the applied voltages were 150 V and 6 V for funnels 1 and 2, respectively. The voltage of skimmer 1 was 30 V and the skimmer 2 voltage was 5 V. Accumulation time in the hexapole collision cell was 0.7 s; the time-of-flight was 2 ms. The operating pressure in UHV region was ~5×10^−10^ mbar. Data acquisition was performed using the FtmsControl software (version 2.2). The time-domain signal, consisting of the sum of 25 transients, containing 2M data points per transient, was subjected to one zero-fill prior to Fourier-transform.

### Molecular dynamics simulations

All simulations were performed without any spatial restraints on the structure of PgaA-220-340 and the protein structure was well retained during simulations, as indicated by the low Cα RMSD value of 0.25 nm after 800 ns (**S26 Fig**).

#### System setup for PgaA in solutions of monosaccharides

The all-atom model consisted of the PgaA protein and monosaccharides (either GlcNAc or GlcN), solvated by explicit water molecules. A total of 21 GlcNAc or GlcN monomers were added to each system to maintain a sugar concentration of 50 mM. For PgaA with GlcNAc and with GlcN, 1 and 21 Cl^-^ ions were added to neutralize the net charge of the systems, respectively, and 44 Na^+^ and 44 Cl^-^ ions were added to maintain a salt concentration of 100 mM. 20 simulation replicas were built with a box size of 9.0 × 9.0 × 9.0 nm^3^ for each model system. For all 40 replicas, initial velocities were generated by a random seed.

#### System setup for PgaA in solutions of trisaccharides

The all-atom simulation system was composed of the PgaA protein and molecules of trisaccharides of the same type, solvated by explicit water molecules. A total of 3 trisaccharides were added to each system to maintain a sugar concentration of 7 mM. 1 and 4,Cl^-^ ions were added to neutralize the net charge of the systems systems containing (GlcNAc)_3_ and GlcNAc-GlcN-GlcNAc, respectively. 44 Na^+^ and 44 Cl^-^ ions were added to maintain a salt concentration of 100 mM. For each model system, 40 simulation replicas were built with a box size of 9.0 × 9.0 × 9.0 nm^3^. For all 80 replicas, initial velocities were generated by a random seed.

#### Simulation protocol

All simulations were performed using the GROMACS 5.1.4 simulation package [43]. PgaA and Na^+^ and Cl^-^ ions were modeled by the AMBER99sb force field [44]. The structures of neutral monosaccharide GlcNAc and trisaccharides (GlcNAc)_3_ were generated using the web-based Glycam Biomolecule Builder (http://glycam.org/), and the structures of charged monosaccharide GlcN and trisaccharides, GlcNAc-GlcN-GlcNAc and (GlcNAc)_2_-GlcN, were obtained by modifying the neutral forms using the molecule editor Avogadro [45]. All saccharides were modeled by the GLYCAM force field [46]. The TIP5P water model was used here to avoid possible nonphysical aggregation of sugars at low concentration, according to a previous study [47]. The systems were first energy-minimized using the steepest descent method, followed by a pre-equilibration phase of 2 ns with quadratic position restraints on the protein backbone using a force constant of 1000 kJ mol^-1^ nm^-2^. Subsequently, simulation systems consisting of protein with sugar monomers, with sugar dimers, and with sugar trimers were run without restraints for 300 ns, 300 ns, and 800 ns, respectively. Lennard-Jones interactions were evaluated using a distance cutoff of 1.2 nm. Coulomb interactions were calculated using the smooth particle mesh Ewald method with a real-space cutoff of 1.1 nm [48,49]. Covalent bonds with H atoms were constrained using the P-LINCS algorithm [50]. No dispersion correction was applied. Simulation in the isothermal-isobaric ensemble was achieved by isotropic coupling to a Parrinello-Rahman barostat at 1 bar with coupling constants of 2 ps [51]. The aqueous solution, sugar, and protein were coupled separately to a temperature bath at 300 K with a coupling constant of τ_T_ = 0.4 ps using the Berendsen algorithm [52]. The leap-frog stochastic dynamics integrator integrator was used and the integration time step was 2 fs [53]. Taken together, the total sampling times for simulations of PgaA with sugar monomers and trimers are 12 and 96 μs, respectively.

#### Analysis

The contacts between the protein and the sugars were computed on all non-hydrogen atoms within a cutoff distance of 0.45 nm. The definitions of metrics for characterizing sugar orientations are illustrated in **S14 Fig**. The angle ω_1_ is defined as the angle between the plane of the TRP indole and the plane of the GlcNAc ring. The distance d_1_ is defined as the distance between the center of mass (COM) of indole and the COM of the GlcNAc ring. Similarly, the angle ω_2_ is defined as the angle between the plane of the ASP side chain and the plane of the GlcN ring. The distance d_2_ is defined as the distance between the Cγ atom of ASP and the nitrogen atom of GlcN. Definitions of the angles θ_1_ and θ_2_ are provided in Figs 3 and 4, respectively. Basins were arbitrarily defined by selecting a center and appropriate boundaries; the occupancy of each basin was calculated by dividing the number of frames belonging to the basin by the total number of frames. VMD was used to make snapshots and calculate the spatial distribution function [54]. The MDTraj package was incorporated in all analysis [55].

### Graphical representation of macromolecules

Molecular Graphics were generated with PyMOL Molecular Graphics System (DeLano Scientific, http://www.pymol.org/), which is curated by SBGrid [56].

## Supporting information

S1 Fig Construct design for PgaA/B pulldown experiments. A) The TPR domain without porin (PgaA-32-515) is not detected in the elution. B) The TPR construct without the porin (PgaA-32-515) domain is prone to degradation. Two major bands well below the expected molecular weight of the protein (55 kDa) are observed. C) Elution profile from size exclusion chromatography using a Bio-Rad ENrich SEC650 column. D) Mass spectrometry analysis of fragmented PgaA-32-515. Samples were extracted from a 10% SDS-polyacrylamide gel. Detected peptides are highlighted in yellow, sites for potential posttranslational modifications are highlighted in green.
(PNG)

S2 Fig PgaA and PgaB do not form a complex when separately purified and mixed, only when co-expressed or co-purified. A) PgaA and PgaB were separately expressed and purified, and then mixed in a 1:1 ratio before analysis by size exclusion chromatography using a Bio-Rad ENrich SEC650 column. PgaA (green) elutes at 12 ml, PgaB-22-672 (red) at 14 ml. The mixture of PgaA and PgaB-22-672 (blue) contains no additional peak or shift or reduction of the peak corresponding to PgaB, indicating no interaction on the column. B) Proteins were separately expressed and then co-purified by mixing cell-pellets (1:1 ratio by weight) before cell lysis, and compared to the co-expressed complex. The complex obtained by co-expression elutes at 11 ml. Peaks in the co-purified samples at 11 ml contain both PgaA and PgaB, indicating complex formation. This is true for both soluble PgaB-22-672 and full-length PgaB.
(PNG)

S3 Fig Crystal structure of PgaA-220-367 contains a domain swap involving TPR-5 and crystal packing interactions involving the binding grooves. A) TPR-5 of Chain A interacts with a symmetry related molecule. Chain A is colored green and its symmetry mate Chain A’ is colored in orange. B) The binding pocket of Chain A is occluded by residues from Chain B and a symmetry related molecule Chain A’. The binding pocket of Chain B is occluded by residues from Chain A and Chain A’. Residues lining the binding groove (R237, W314, Y317) are shown in pink and magenta for Chain A and B, respectively. Hydrogen bonds and salt-bridges are indicated by dashed lines. The following aromatic interactions occur: Y317-A with F340-B, W314-A with H342-B, Y317-B with F-340-A.
(PNG)

S4 Fig The crystallized TPR module displays an increased superhelical curvature. Comparison of the crystal structure of PgaA-220-367 with the O-linked GlcNAc-transferase (OGT, grey) (PDB 1W3B) [18]. When TPR-3 (blue) and TPR-4 (purple) are aligned with the TPR motifs of OGT (rmsd_Cα_ = 1.7 Å for 62 aligned residues), the α/α-5 motif (green) differs noticeably (rmsd_Cα_ = 3.1 Å for 32 compared residues) and appears to increase the curvature of the crystallized module by a shift towards the concave surface.
(PNG)

S5 Fig Mass spectrometry analysis reveals that the dPNAG sample is a mixture of partially de-acetylated 6,7,8-mers. ESI mass spectrum was obtained using a G2S ESI-Q-IMS-TOF mass spectrometer in positive mode for a 50 mM aqueous ammonium acetate (pH 7) solution of dPNAG.
(PNG)

S6 Fig Stability control for PgaA-220-367 mutants. A) Size exclusion profiles on a SEC650 column. B) Coomassie stained SDS PAGE gel. C) Circular dichroism (CD) wavelength scan. D) CD melting curves at 222 nm. Tm values and standard errors were obtained by a Boltzmann sigmoidal fit using GraphPad Prism version 6.0 for Mac OS X.
(PNG)

S7 Fig C-terminal TPR constructs are insoluble when expressed in the cytoplasm of *E*. *coli* BL21 cells. Constructs are labelled; L and S represent the lysate and soluble fractions after cell lysis. PgaA-352-502, 368–482, 368–515 and 352–464 were lysed in buffer containing 50 mM Tris pH 7, 300 mM NaCl. PgaA-401-515 and 368–502 were lysed in 50 mM Tris pH8, 300 mM NaCl 10 mM imidizole and 5% (v/v) glycerol.
(PNG)

S8 Fig Representative ESI mass spectrum of Pga-32-220. Mass spectrum was acquired using a 15 T FT-ICR mass spectrometer in positive mode for an aqueous ammonium acetate (200 mM, pH 7) solution of PgaA-32-220 (4 μM). Inset shows PgaA-32-220 ion peaks (at charge state +8) corresponding to different species and molecular weight values of PgaA-32-220.
(PNG)

S9 Fig ESI mass spectra of PgaA-32-220 with ligands. Mass spectra were acquired using a 15 T FT-ICR mass spectrometer in positive mode for aqueous ammonium acetate (200 mM, pH 7) solutions of PgaA-32-220 (4 μM) and (top) PNAG (82 μM), (bottom) dPNAG (60 μM).
(PNG)

S10 Fig Representative ESI mass spectrum of PgaA-220-367. Mass spectrum was acquired using a G2S ESI-Q-IMS-TOF mass spectrometer in positive mode for an aqueous ammonium acetate (200 mM, pH 7) solution of PgaA-220-367 (11.6 μM).
(PNG)

S11 Fig Representative ESI mass spectra of PgaA-220-367 with ligands. Mass spectra were acquired using a G2S ESI-Q-IMS-TOF mass spectrometer in positive mode for aqueous ammonium acetate (200 mM, pH 7) solutions of PgaA-220-367 (P, 11.6 μM), reference protein (Se155-4 single chain variable fragment, P_ref_, 1 μM) and (top) PNAG (82 μM), (middle) dPNAG (60 μM) and (bottom) human milk pentasaccharide LNF1 (75 μM).
(PNG)

S12 Fig Representative ESI mass spectrum of PgaA-32-367. Mass spectrum was acquired using a G2S ESI-Q-IMS-TOF mass spectrometer in positive mode for an aqueous ammonium acetate (200 mM, pH 7) solution of PgaA32-367 (5.7 μM). MW of PgaA-32-367 major species is 40 570 Da. On the left side (at lower m/z range) ion peaks corresponding to unfolded protein can be observed.
(PNG)

S13 Fig Representative ESI mass spectra of PgaA-32-367 with ligands. Mass spectra were acquired using a G2S ESI-Q-IMS-TOF mass spectrometer in positive mode for aqueous ammonium acetate (200 mM, pH 7) solutions of PgaA-32-367 (P, 5.7 μM), reference protein (Se155-4 single chain variable fragment, P_ref_, 1 μM) and (top) PNAG (82 μM), (middle) dPNAG (60 μM) and (bottom) human milk pentasaccharide LNF1 (75 μM).
(PNG)

S14 Fig Definitions of metrics for describing sugar orientations. Schematic illustrations of (A) the plane rotation angle θ_1_, plane tilt angle ω_1_, and the plane distance d_1_ for the interaction pair GlcNAc–TRP; (B) the plane rotation angle θ_2_, plane tilt angle ω_2_, and the aspartate-C_**γ**_ to GlcN-N^+^ distance d_2_ for the interaction pair GlcN–ASP. The angles θ_1_ and θ_2_ are defined as the angles between the vectors V_1_ and V_2_, while the angles ω_1_ and ω_2_ are defined as the angles between the vectors V_3_ and V_4_. The distance d_1_ is defined as the distance between the center of mass (COM) of TRP sidechain and the COM of GlcNAc.
(JPG)

S15 Fig Interaction of W314 with GlcNAc. (A) 2D histogram of the distributions of the plane tilt angle ω_1_ and plane distance d_1_ for GlcNAc binding to W314. (B) Schematic illustration of the plane rotation angle θ_1_, which is defined as the angle between V_1_ and V_2_. (C) Representative snapshots of the basin highlighted by a dashed cyan box in (A). Residue W314 is shown in blue and GlcNAc in yellow, two orientations 90 degrees apart are provided. (D) Average number of GlcNAc monomers bound to PgaA residues. The residues with the three highest average numbers of bound ligand are labelled. (E) Distribution of the plane rotation angle θ_1_ for the conformational basin highlighted by the dashed cyan box in (A).
(PNG)

S16 Fig Interaction of D230 with GlcN. (A) 2D histogram of the distributions of plane tilt angle ω_2_ and distance d_2_ for GlcN binding to D230. (B) Schematic illustration of the plane rotation angle θ_2_, which is defined as the angle between V_1_ and V_2_. (C) Average number of GlcN monomers bound to PgaA residues. (D) Distribution of the plane rotation angle θ_2_ for the basin of conformations highlighted by a dashed cyan box in (A). (E) Representative snapshots of the basin of conformations highlighted by the dashed cyan box in (A). Residue D230 is shown in blue and GlcN in green, two orientations 90 degree apart are provided.
(PNG)

S17 Fig Interaction of W314 with (GlcNAc)_3_. (A) 2D histogram of the distributions of plane tilt angle ω_1_ and plane distance d_1_ for (GlcNAc)_3_ binding to W314. The populations of conformational basins I, II, and III are 11 ± 4%, 5 ± 2%, and 10 ± 3%, respectively. (B) Representative snapshots of basins I, II and III. Residue W314 is shown in blue and (GlcNAc)_3_ in yellow, two orientations 90 degrees apart are provided as well as a view of the entire TPR module. (C) Average number of (GlcNAc)_3_ trimers bound to PgaA residues. (D) Distribution of the plane rotation angle θ_1_ for basins I, II and III.
(JPG)

S18 Fig Interaction of W314 with GlcNAc-GlcN-GlcNAc. (A) 2D histogram of the distributions of plane tilt angle ω_1_ and plane distance d_1_ for GlcNAc-GlcN-GlcNAc binding to W314. Populations of basins I, II, and III are 3 ± 2%, 3 ± 2%, and 10 ± 3%, respectively. (B) Representative snapshots of conformational basins I, II and III. Residue W314 is shown in blue and GlcNAc-GlcN-GlcNAc in pink; two orientations 90 degrees apart are provided as well as a view of the entire TPR module. (C) Averaged number of GlcNAc-GlcN-GlcNAc trimers bound to PgaA residues. (D) Distribution of the plane rotation angle θ_1_ for conformational basins I, II and III.
(PNG)

S19 Fig Interaction of D230 with GlcNAc-GlcN-GlcNAc. (A) 2D histogram of the distributions of plane tilt angle ω_2_ and distance d_2_ for GlcNAc-GlcN-GlcNAc binding to D230. Populations of conformational basins I, and II are 11 ± 4% and 5 ± 2%, respectively. (B) Representative snapshots of binding modes I and II. Residues W314 and D230 are shown in blue and GlcNAc-GlcN-GlcNAc in pink; two orientations 90 degree apart are provided as well as a view of the entire TPR module. (C) Average number of GlcNAc-GlcN-GlcNAc trimers bound to PgaA residues. (D) Distribution of the plane rotation angle θ_2_ for the conformation basins I and II defined in (A).
(JPG)

S20 Fig Directional analysis of trimers bound to PgaA-220-340. Projection of interactions of PgaA-220-340 with (A) (GlcNAc)_3_ or (C) GlcNAc-GlcN-GlcNAc on a 2D plane. All sugar conformations that involve interaction with either W314 or D230 are projected. The protein residues are projected as grey dots, with residues W314, W318, R279, R237, D230, F240 and Y317 highlighted as red stars. The coordinate origin is centered on W314. Sugar trimers are projected as orange and cyan arrows, corresponding to conformations with reducing ends pointing downward and upward, respectively. (B) Representative snapshots of sugar conformations with different orientations of reducing end (indicated by a black star), and the definition of arrows. The populations of conformations with reducing ends pointing downward (orange) or upward (cyan) are included at the top right corner of panels. The definition of the vector used for this analysis is shown by a dashed arrow.
(PNG)

S21 Fig Potential dPNAG pathways on PgaA-220-340. Potential pathways are labelled as A, B, C, and D. Residues that are frequently involved in contacts with dPNAG, including D230, R237, F240, R279, W314, Y317, and W318, are highlighted in the cartoon representation in the first column from left. Snapshots of sugar trimers adopted from either the same simulation or other simulations were imposed on the protein structure, as shown in the second and third columns. Schematic representations of potential dPNAG pathways are shown in the fourth column (right). The star indicates the reducing end of the trimer.
(JPG)

S22 Fig Comparison of TPR and Sel1-like superhelices. The left side shows OGT (PDB 1W3B, residues 77–383) [18] as an example for a TPR superhelix. The right side shows AlgK (PDB 3E4B, residues 71–388) [3] as an example for a Sel1-like superhelix. Both proteins contain 9 repeat motifs. At the top is a view along the superhelical axis, at the bottom is a side view. Both proteins are in rainbow colors with the N-terminus in blue and the C-terminus in red.
(PNG)

S23 Fig Generation of a homology model of PgaA. The Phyre^2^ model [21] of PgaA-38-502 is based on the crystal structure of OGT (PDB 1W3B) [18]. The orientation of the PgaA porin (PDB 4Y25) [6] towards the TPR domain is based on a superposition of a terminal TPR motif from a BcsC crystal structure (PDB 6TZK) [20] onto TPR8.
(PNG)

S24 Fig Alignment of the PgaA and the BcsC porin. The sequence identity between both porins is 10.6%. Secondary-structure matching (SSM) alignment [58] is possible (rmsd_Cα_ = 2.9 Å) when most extracellular loops are ignored, excluded loops shown as dotted lines. (A) Rainbow color scheme from the N-terminal end (blue) to the C-terminal end (red) shows that all 16 beta-strands align in register (strand 1 of BcsC with strand 1 of PgaA, etc). (B) Coloring by chain reveals slight differences in the oval shape, explaining the elevated rmsd value. Alignments were performed in Coot [38].
(PNG)

S25 Fig Evaluation of the AF2 model of PgaA. A) Comparison between crystal structure and AF2 model of PgaA-224-342. B) Confidence score of AF2 model of PgaA-32-807. The protein is colored by confidence level; solid red denotes high confidence (>90), solid blue denotes low confidence (<50).
(PNG)

S26 Fig Time evolution of RMSD of PgaA-220-340 during MD simulations. RMSD was computed for protein Cα atoms relative to the corresponding crystal structure PgaA-220-340. RMSD values in the time ranges of 0–300 ns and of 300–800 ns were averaged over all datasets and only datasets of sugar trimers, respectively. The RMSD value reached 0.25 nm after 800 ns. Standard deviation of mean was shown in red shadow.
(PNG)

S1 Table Summary of simulated systems. (DOCX)

S2 Table List of strains, plasmids, and primers used in this report. (DOCX)
