# Supplementary figures and images for: The TPR domain of PgaA is a multifunctional scaffold that binds PNAG and modulates PgaB-dependent polymer processing
Source: PLoS Pathog. 2022 Aug 5;18(8):e1010750. doi: 10.1371/journal.ppat.1010750 (PMC9384988; doi:10.1371/journal.ppat.1010750)

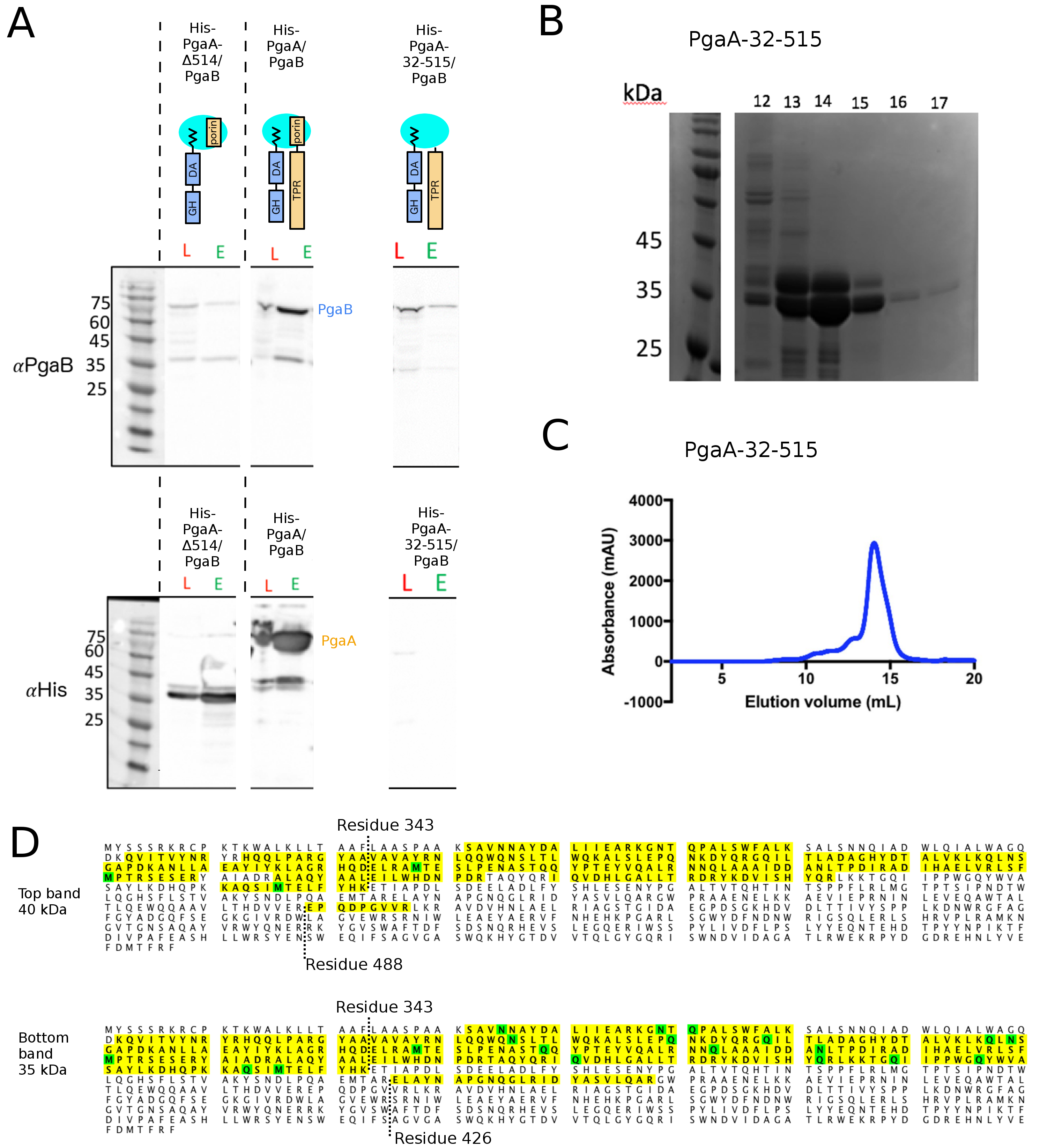

Supplement: S1 Fig — A) The TPR domain without porin (PgaA-32-515) is not detected in the elution. B) The TPR construct without the porin (PgaA-32-515) domain is prone to degradation. Two major bands well below the expected molecular weight of the protein (55 kDa) are observed. C) Elution profile from size exclusion chromatography using a Bio-Rad ENrich SEC650 column. D) Mass spectrometry analysis of fragmented PgaA-32-515. Samples were extracted from a 10% SDS-polyacrylamide gel. Detected peptides are highlighted in yellow, sites for potential posttranslational modifications are highlighted in green. (PNG) [file ppat.1010750.s001.png]

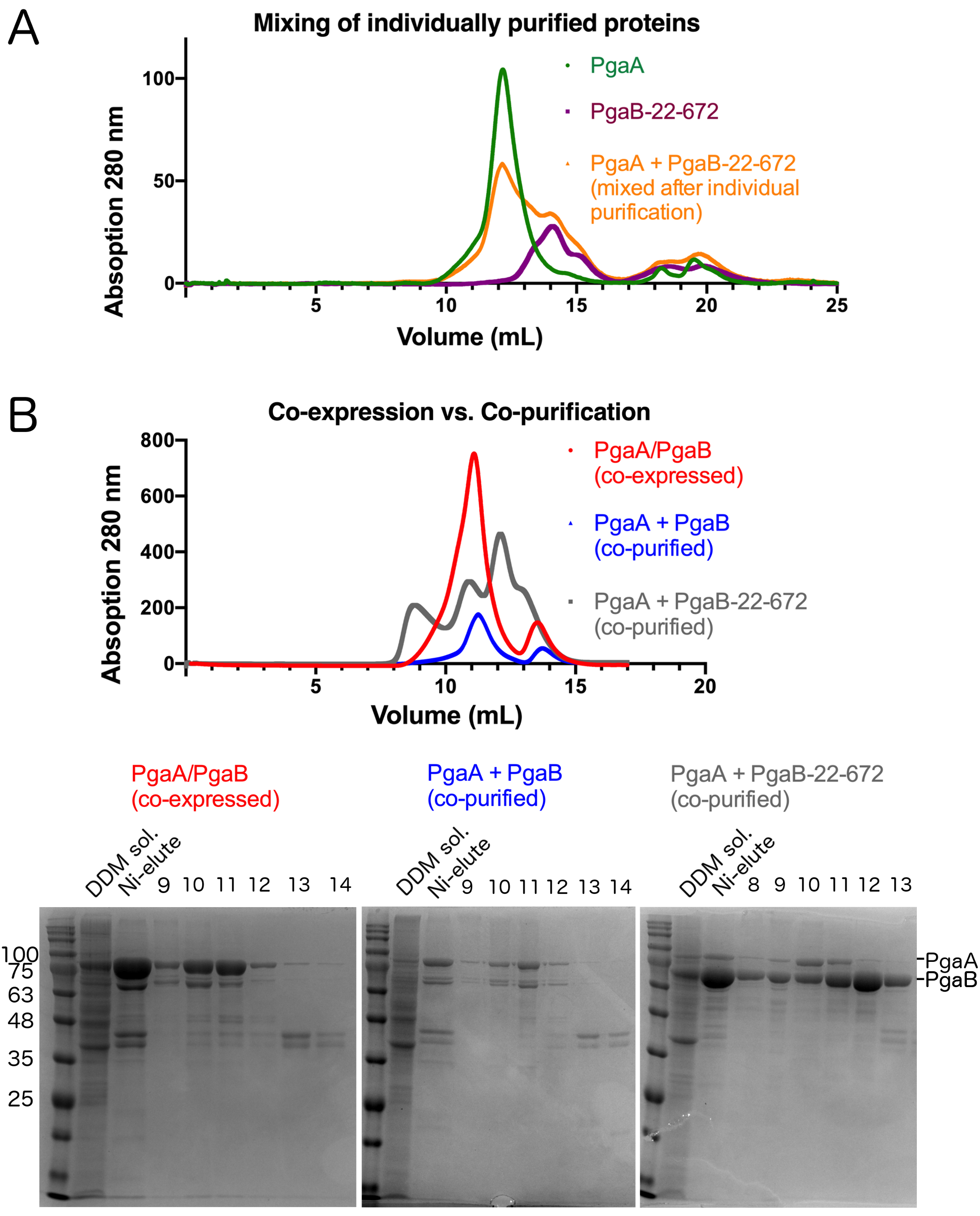

Supplement: S2 Fig — A) PgaA and PgaB were separately expressed and purified, and then mixed in a 1:1 ratio before analysis by size exclusion chromatography using a Bio-Rad ENrich SEC650 column. PgaA (green) elutes at 12 ml, PgaB-22-672 (red) at 14 ml. The mixture of PgaA and PgaB-22-672 (blue) contains no additional peak or shift or reduction of the peak corresponding to PgaB, indicating no interaction on the column. B) Proteins were separately expressed and then co-purified by mixing cell-pellets (1:1 ratio by weight) before cell lysis, and compared to the co-expressed complex. The complex obtained by co-expression elutes at 11 ml. Peaks in the co-purified samples at 11 ml contain both PgaA and PgaB, indicating complex formation. This is true for both soluble PgaB-22-672 and full-length PgaB. (PNG) [file ppat.1010750.s002.png]

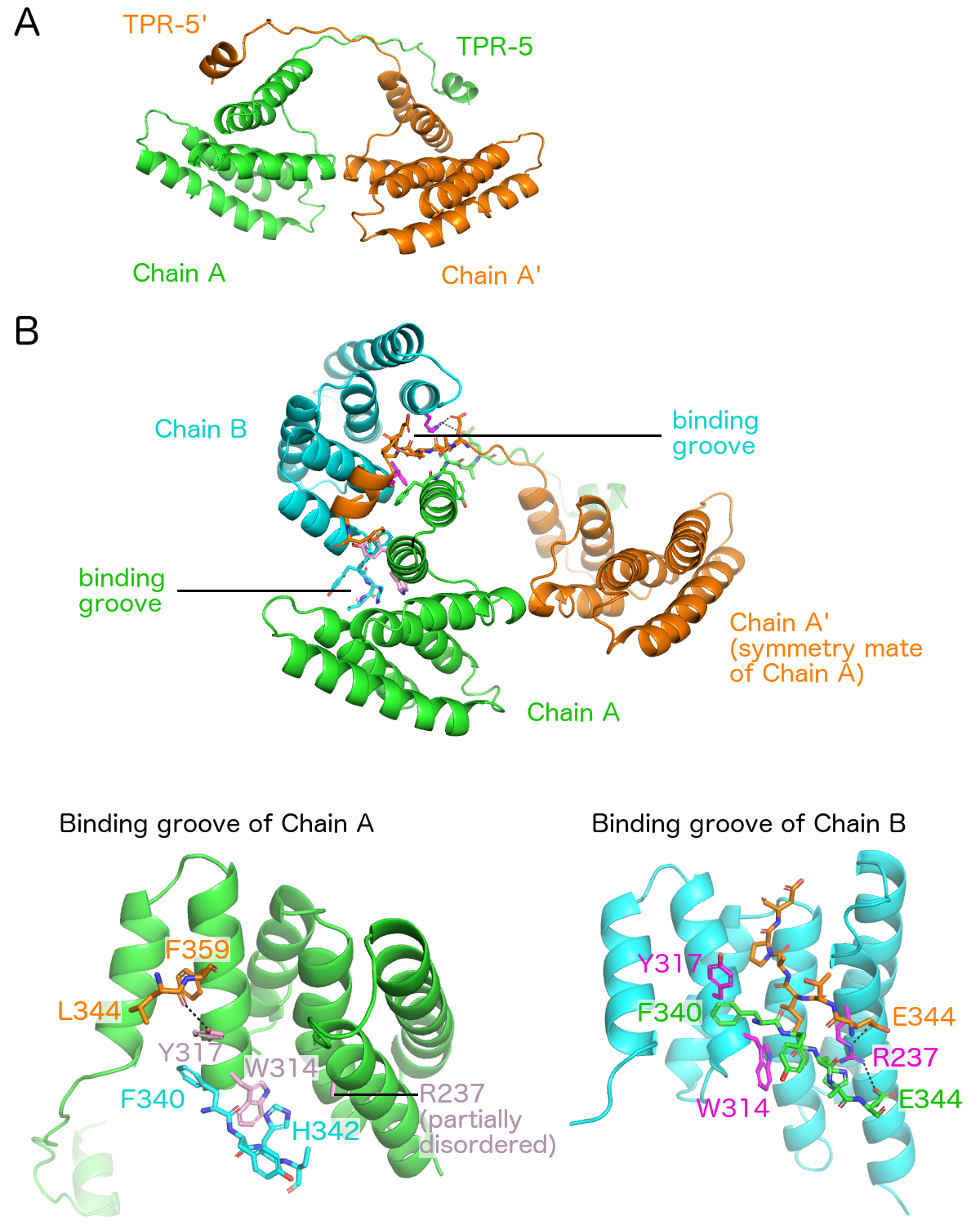

Supplement: S3 Fig — A) TPR-5 of Chain A interacts with a symmetry related molecule. Chain A is colored green and its symmetry mate Chain A’ is colored in orange. B) The binding pocket of Chain A is occluded by residues from Chain B and a symmetry related molecule Chain A’. The binding pocket of Chain B is occluded by residues from Chain A and Chain A’. Residues lining the binding groove (R237, W314, Y317) are shown in pink and magenta for Chain A and B, respectively. Hydrogen bonds and salt-bridges are indicated by dashed lines. The following aromatic interactions occur: Y317-A with F340-B, W314-A with H342-B, Y317-B with F-340-A. (PNG) [file ppat.1010750.s003.png]

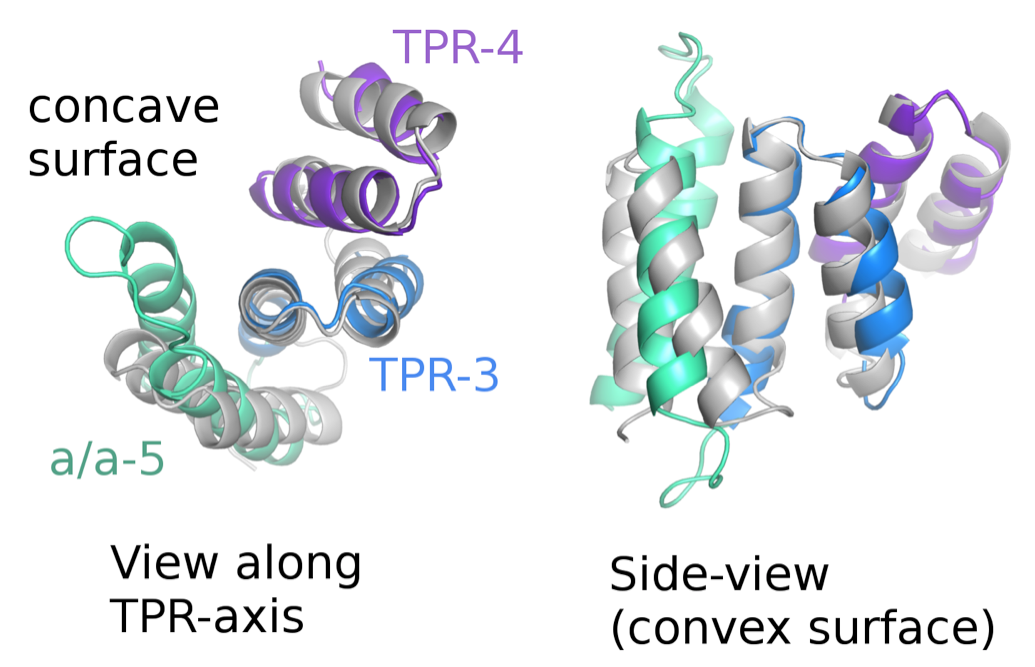

Supplement: S4 Fig — Comparison of the crystal structure of PgaA-220-367 with the O-linked GlcNAc-transferase (OGT, grey) (PDB 1W3B) [18]. When TPR-3 (blue) and TPR-4 (purple) are aligned with the TPR motifs of OGT (rmsdCα = 1.7 Å for 62 aligned residues), the α/α-5 motif (green) differs noticeably (rmsdCα = 3.1 Å for 32 compared residues) and appears to increase the curvature of the crystallized module by a shift towards the concave surface. (PNG) [file ppat.1010750.s004.png]

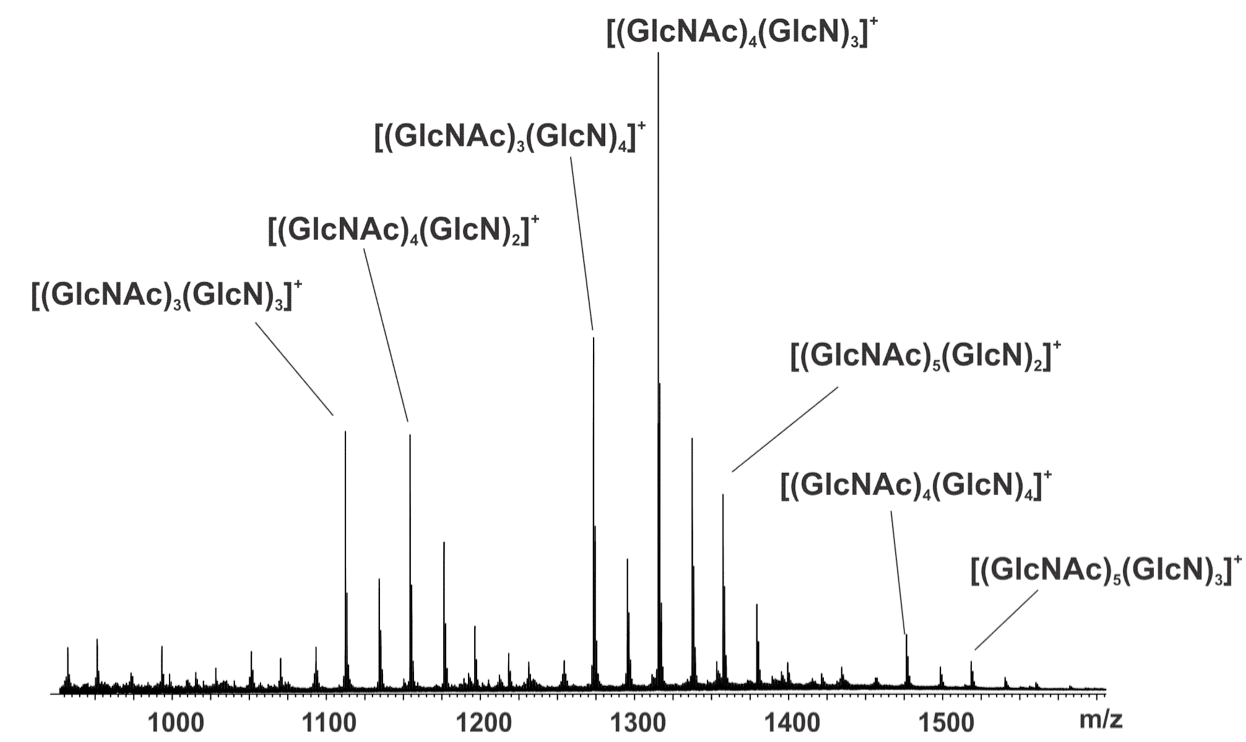

Supplement: S5 Fig — ESI mass spectrum was obtained using a G2S ESI-Q-IMS-TOF mass spectrometer in positive mode for a 50 mM aqueous ammonium acetate (pH 7) solution of dPNAG. (PNG) [file ppat.1010750.s005.png]

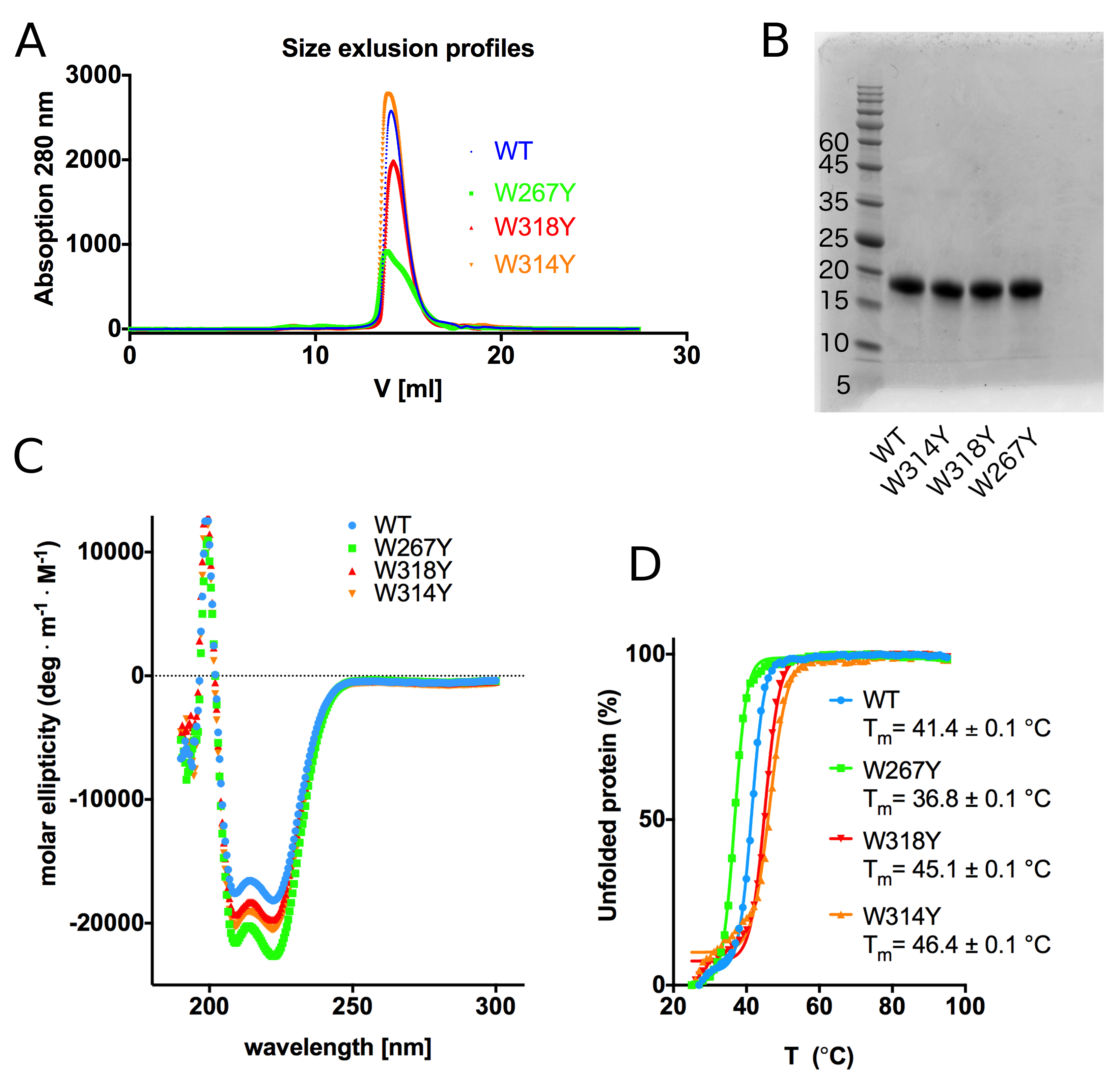

Supplement: S6 Fig — A) Size exclusion profiles on a SEC650 column. B) Coomassie stained SDS PAGE gel. C) Circular dichroism (CD) wavelength scan. D) CD melting curves at 222 nm. Tm values and standard errors were obtained by a Boltzmann sigmoidal fit using GraphPad Prism version 6.0 for Mac OS X. (PNG) [file ppat.1010750.s006.png]

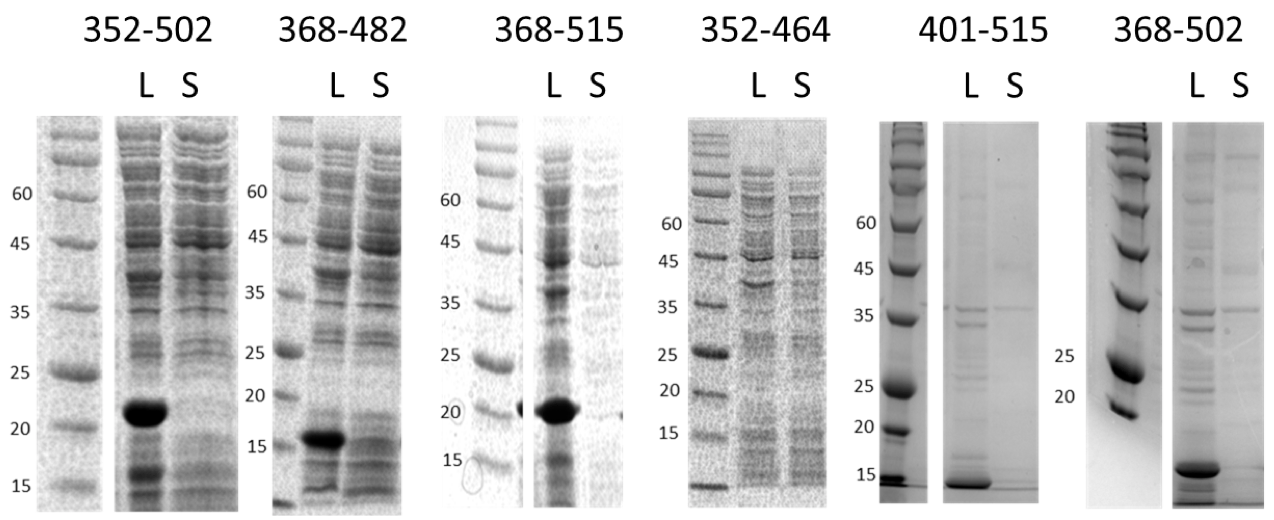

Supplement: S7 Fig — Constructs are labelled; L and S represent the lysate and soluble fractions after cell lysis. PgaA-352-502, 368–482, 368–515 and 352–464 were lysed in buffer containing 50 mM Tris pH 7, 300 mM NaCl. PgaA-401-515 and 368–502 were lysed in 50 mM Tris pH8, 300 mM NaCl 10 mM imidizole and 5% (v/v) glycerol. (PNG) [file ppat.1010750.s007.png]

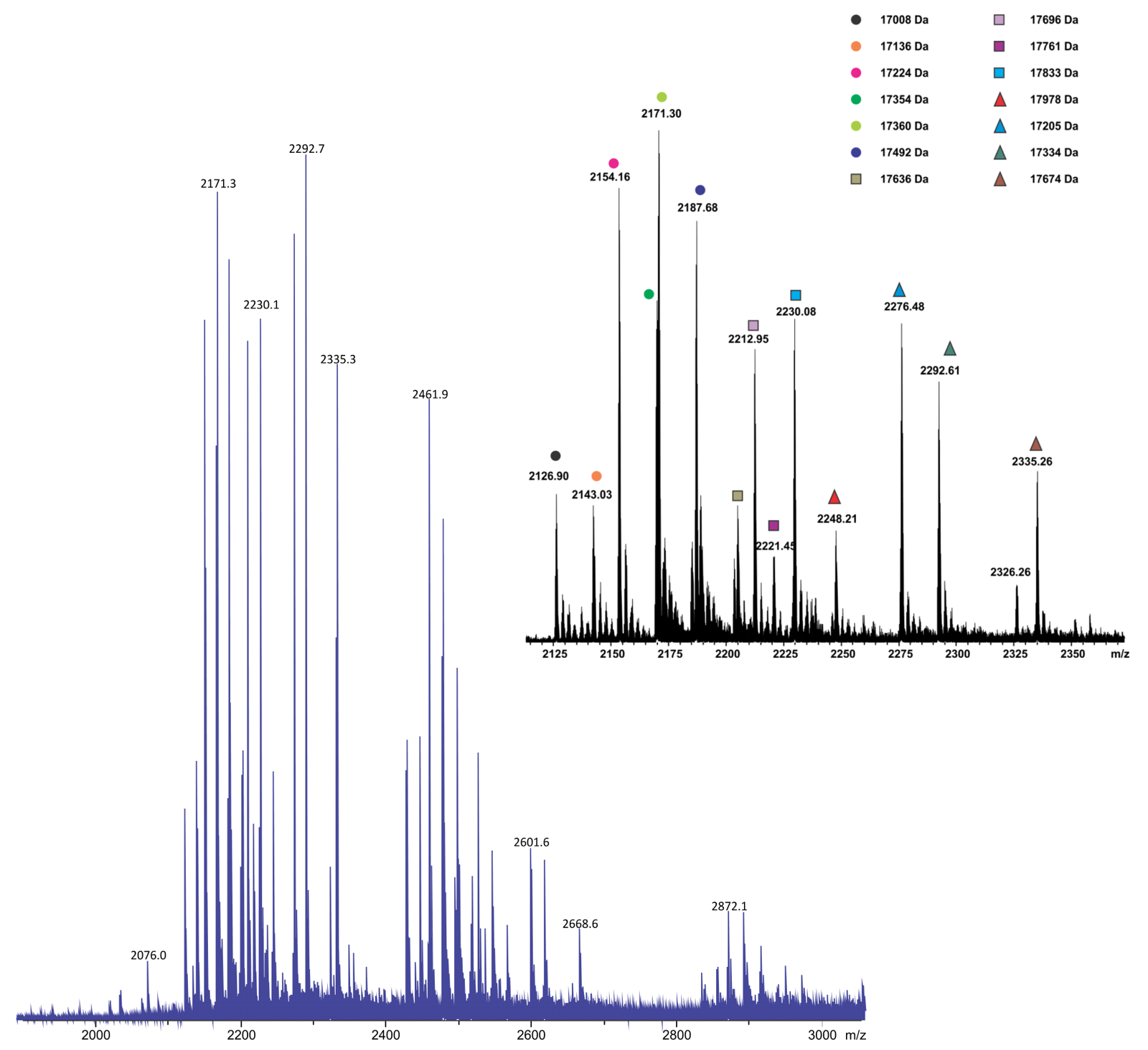

Supplement: S8 Fig — Mass spectrum was acquired using a 15 T FT-ICR mass spectrometer in positive mode for an aqueous ammonium acetate (200 mM, pH 7) solution of PgaA-32-220 (4 μM). Inset shows PgaA-32-220 ion peaks (at charge state +8) corresponding to different species and molecular weight values of PgaA-32-220. (PNG) [file ppat.1010750.s008.png]

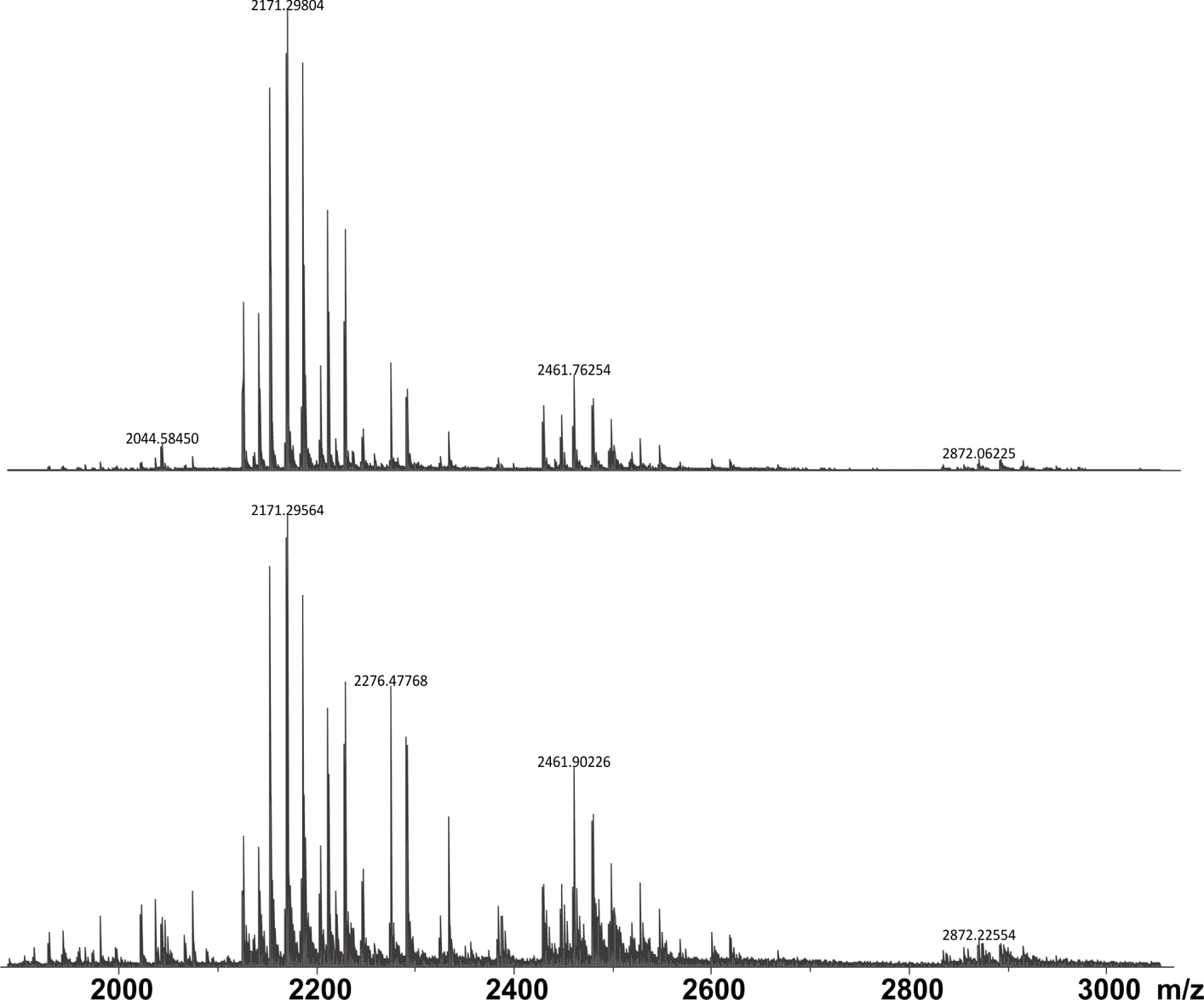

Supplement: S9 Fig — Mass spectra were acquired using a 15 T FT-ICR mass spectrometer in positive mode for aqueous ammonium acetate (200 mM, pH 7) solutions of PgaA-32-220 (4 μM) and (top) PNAG (82 μM), (bottom) dPNAG (60 μM). (PNG) [file ppat.1010750.s009.png]

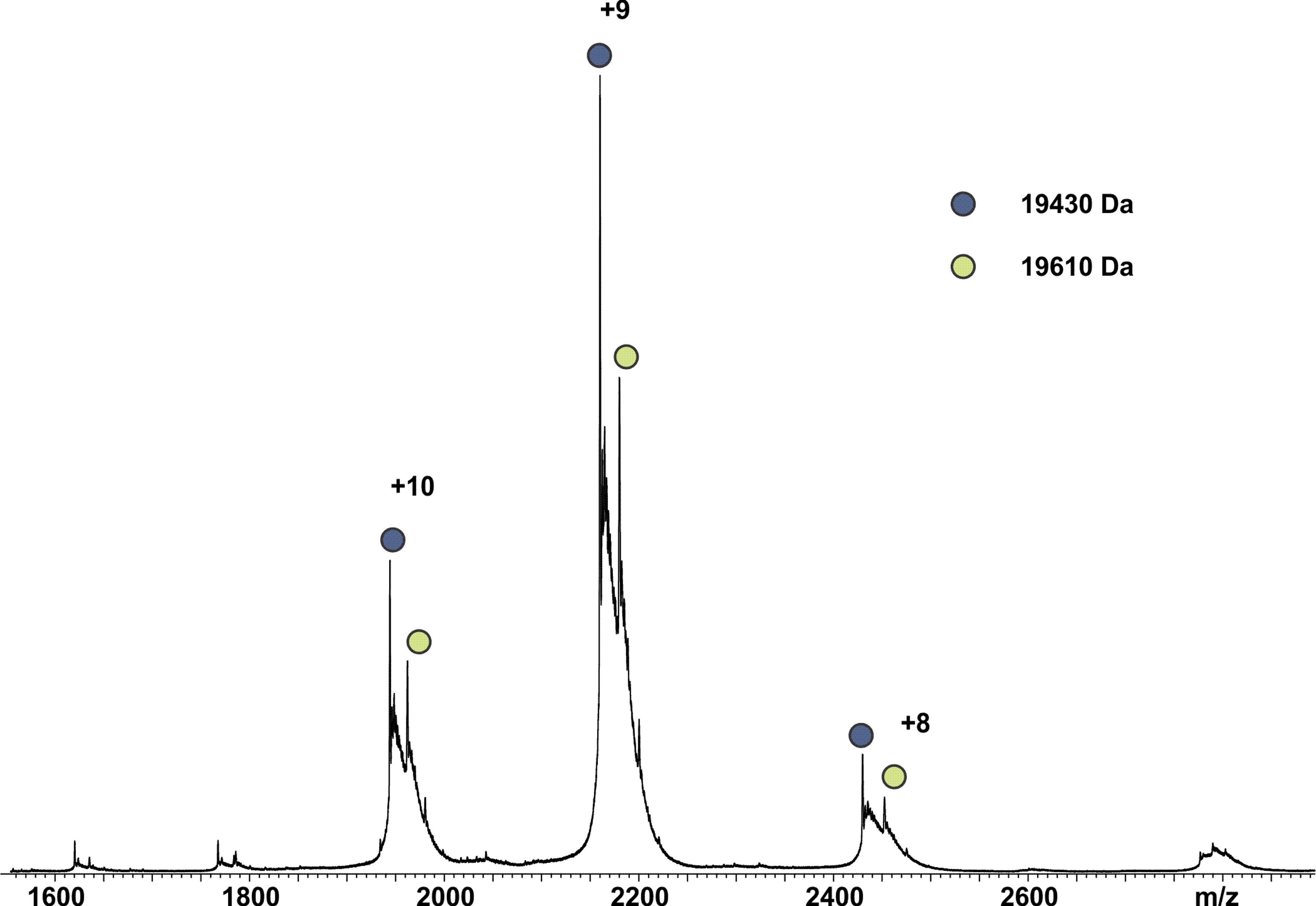

Supplement: S10 Fig — Mass spectrum was acquired using a G2S ESI-Q-IMS-TOF mass spectrometer in positive mode for an aqueous ammonium acetate (200 mM, pH 7) solution of PgaA-220-367 (11.6 μM). (PNG) [file ppat.1010750.s010.png]

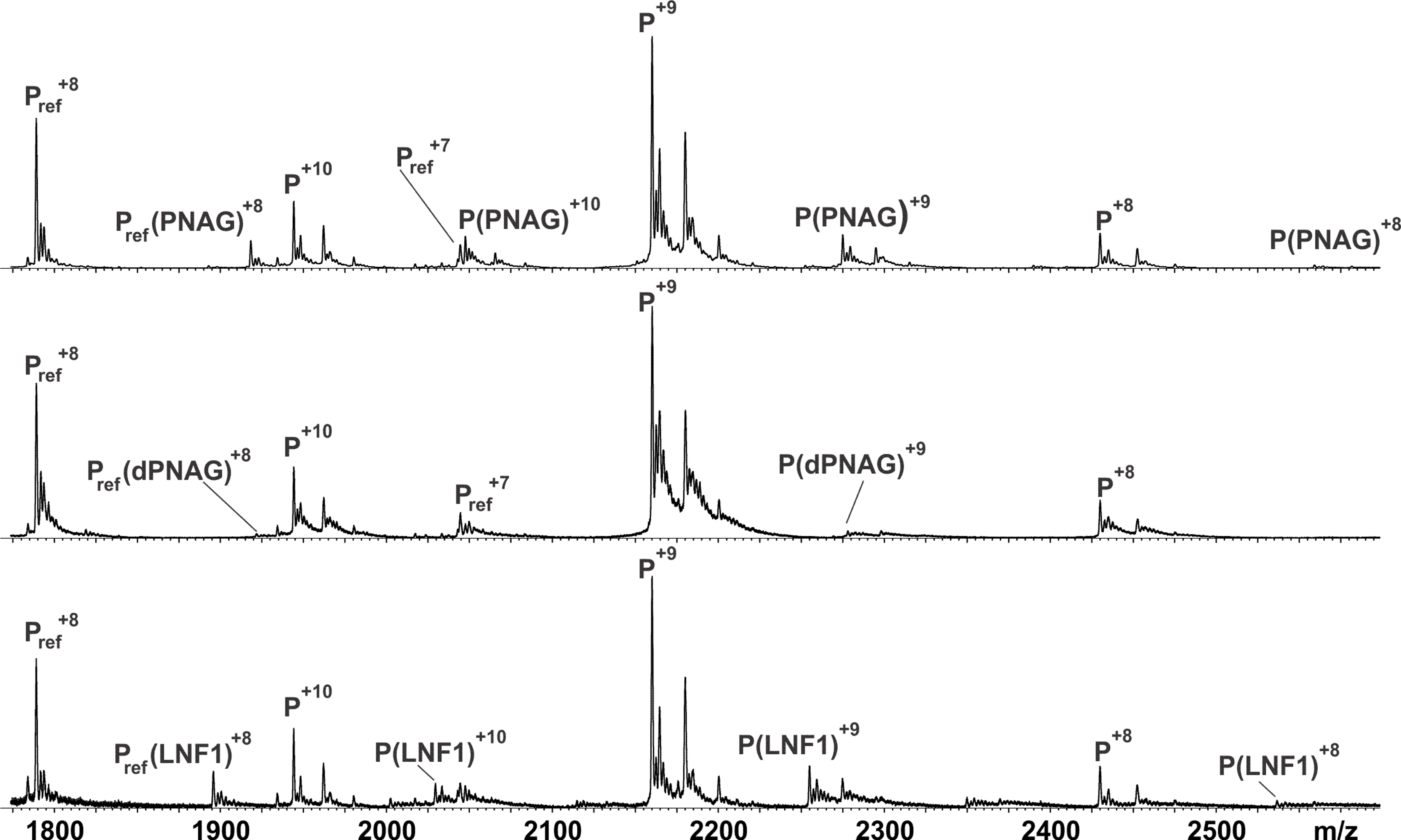

Supplement: S11 Fig — Mass spectra were acquired using a G2S ESI-Q-IMS-TOF mass spectrometer in positive mode for aqueous ammonium acetate (200 mM, pH 7) solutions of PgaA-220-367 (P, 11.6 μM), reference protein (Se155-4 single chain variable fragment, Pref, 1 μM) and (top) PNAG (82 μM), (middle) dPNAG (60 μM) and (bottom) human milk pentasaccharide LNF1 (75 μM). (PNG) [file ppat.1010750.s011.png]

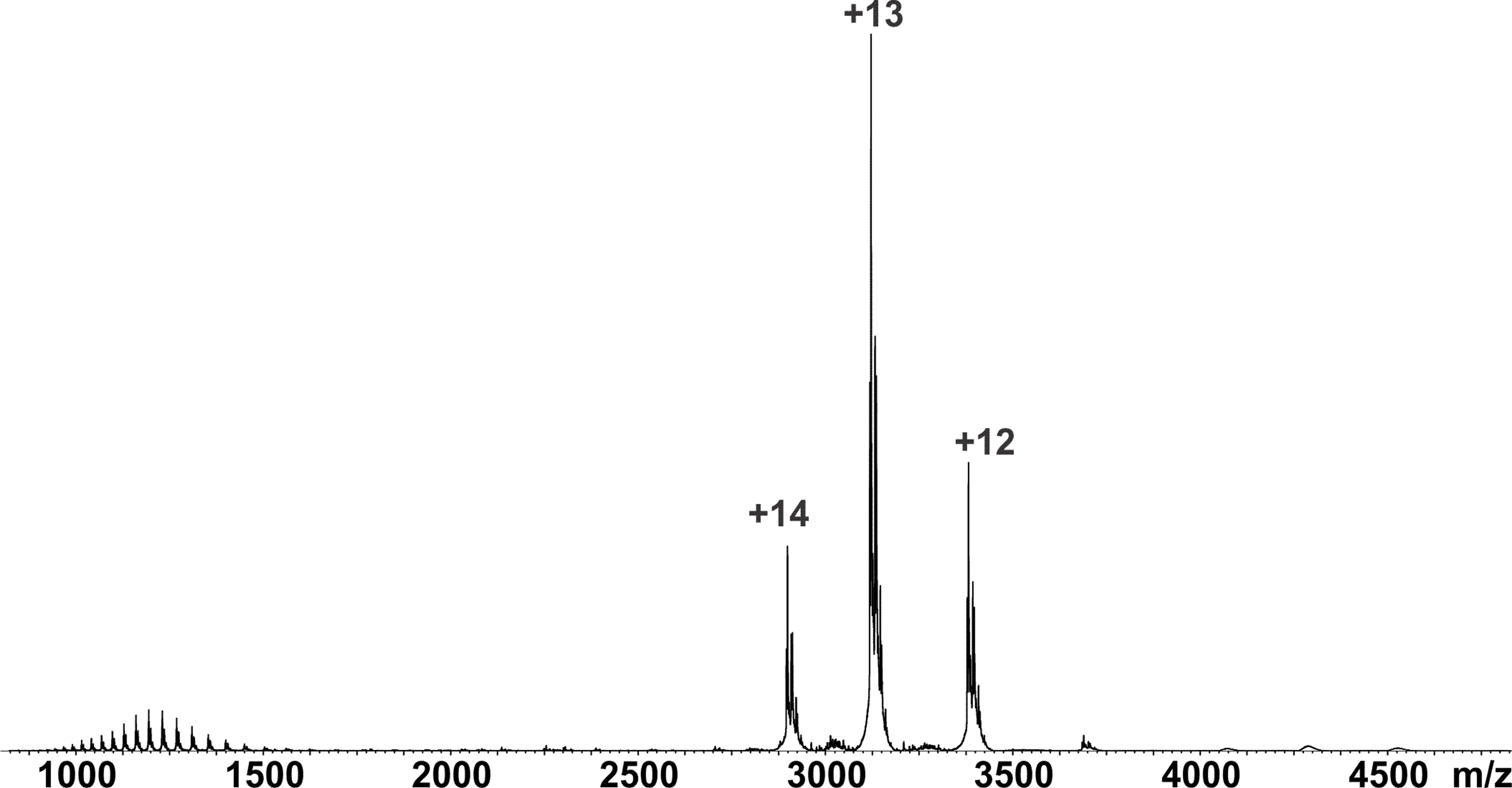

Supplement: S12 Fig — Mass spectrum was acquired using a G2S ESI-Q-IMS-TOF mass spectrometer in positive mode for an aqueous ammonium acetate (200 mM, pH 7) solution of PgaA32-367 (5.7 μM). MW of PgaA-32-367 major species is 40 570 Da. On the left side (at lower m/z range) ion peaks corresponding to unfolded protein can be observed. (PNG) [file ppat.1010750.s012.png]

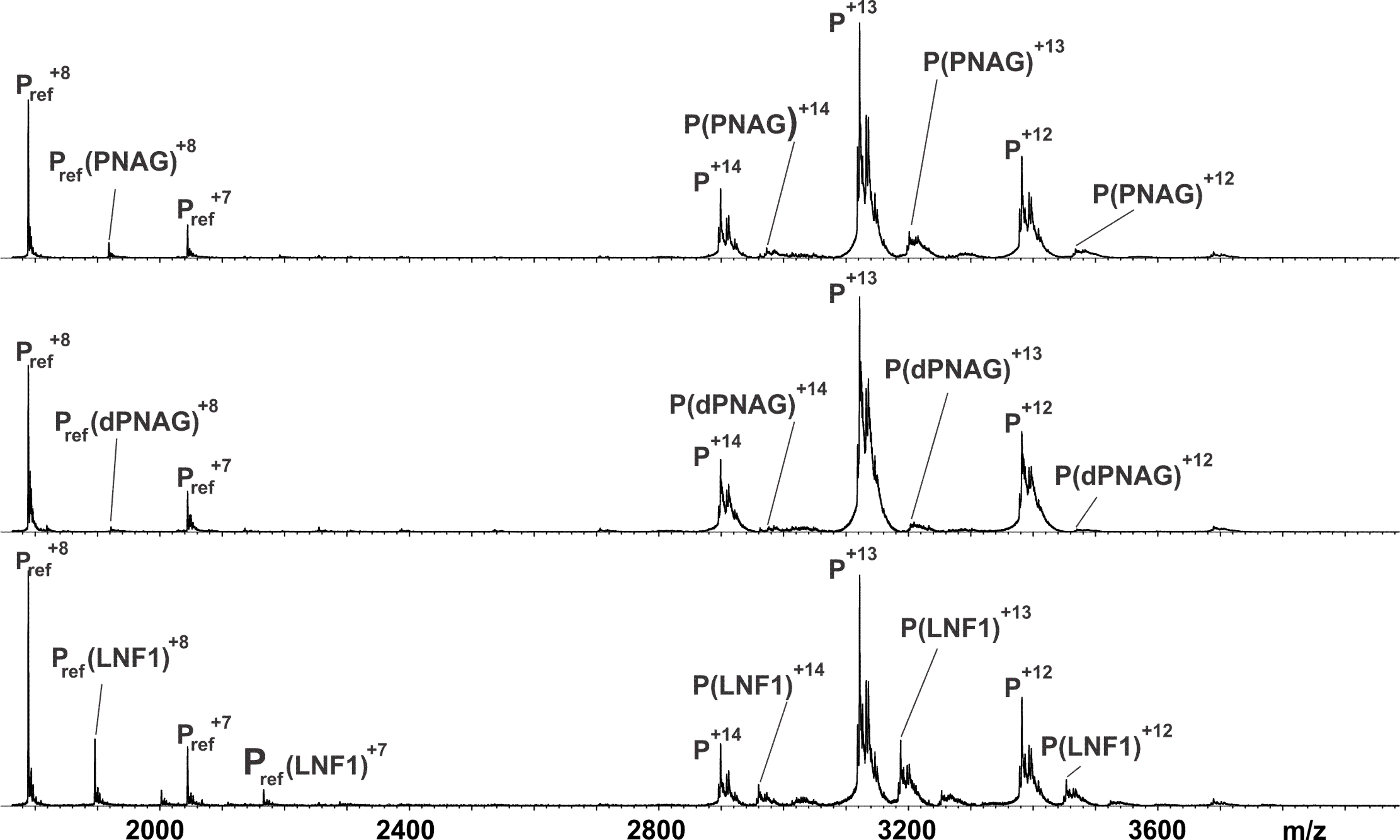

Supplement: S13 Fig — Mass spectra were acquired using a G2S ESI-Q-IMS-TOF mass spectrometer in positive mode for aqueous ammonium acetate (200 mM, pH 7) solutions of PgaA-32-367 (P, 5.7 μM), reference protein (Se155-4 single chain variable fragment, Pref, 1 μM) and (top) PNAG (82 μM), (middle) dPNAG (60 μM) and (bottom) human milk pentasaccharide LNF1 (75 μM). (PNG) [file ppat.1010750.s013.png]

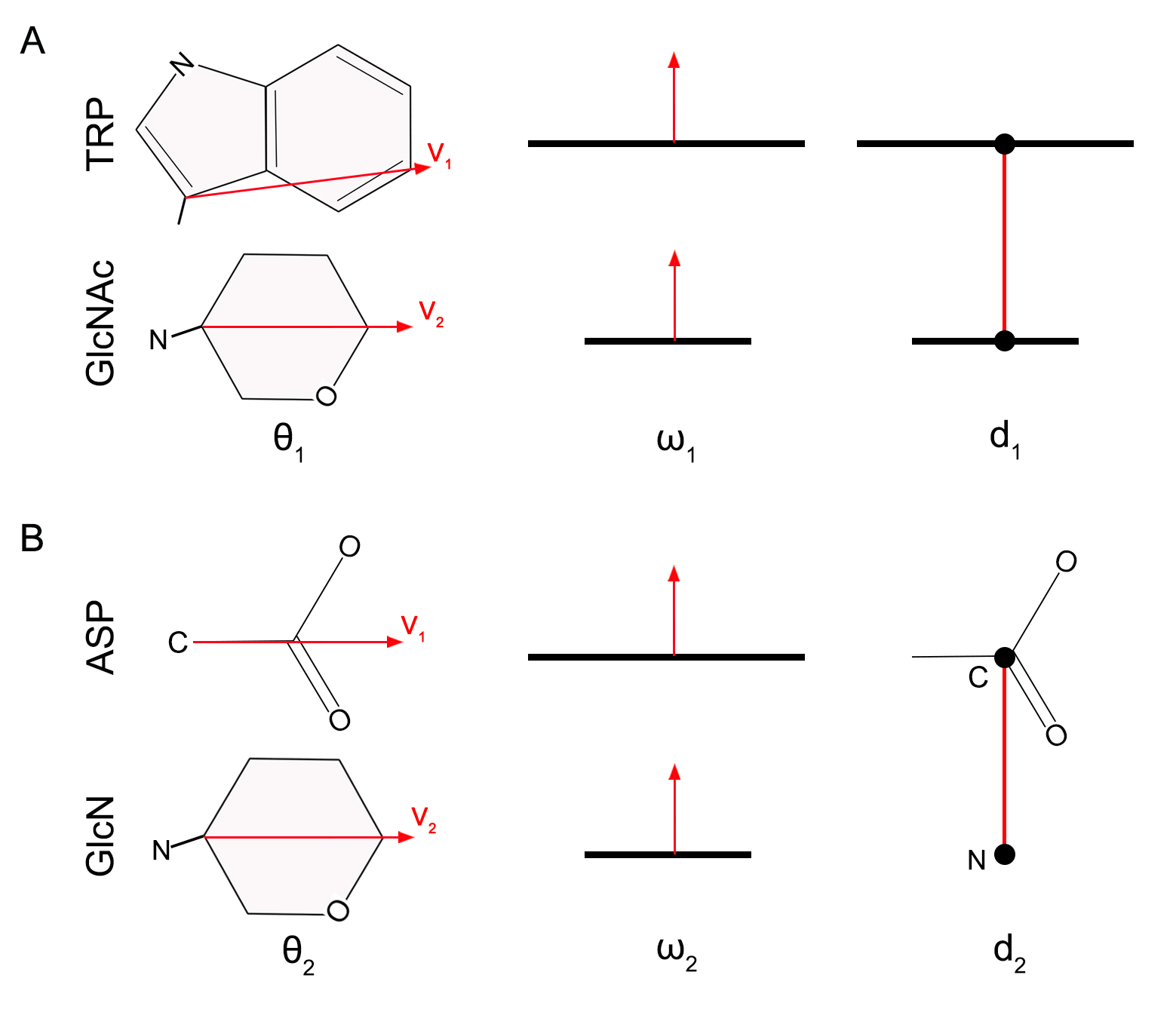

Supplement: S14 Fig — Schematic illustrations of (A) the plane rotation angle θ1, plane tilt angle ω1, and the plane distance d1 for the interaction pair GlcNAc–TRP; (B) the plane rotation angle θ2, plane tilt angle ω2, and the aspartate-Cγ to GlcN-N+ distance d2 for the interaction pair GlcN–ASP. The angles θ1 and θ2 are defined as the angles between the vectors V1 and V2, while the angles ω1 and ω2 are defined as the angles between the vectors V3 and V4. The distance d1 is defined as the distance between the center of mass (COM) of TRP sidechain and the COM of GlcNAc. (JPG) [file ppat.1010750.s014.jpg]

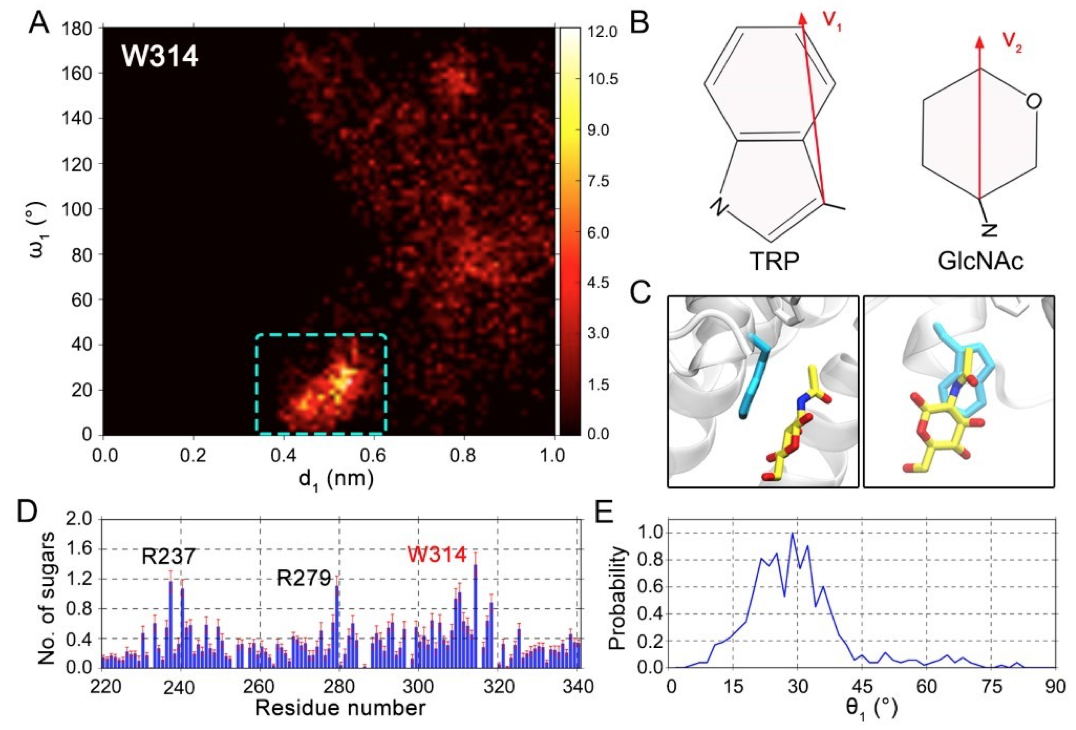

Supplement: S15 Fig — (A) 2D histogram of the distributions of the plane tilt angle ω1 and plane distance d1 for GlcNAc binding to W314. (B) Schematic illustration of the plane rotation angle θ1, which is defined as the angle between V1 and V2. (C) Representative snapshots of the basin highlighted by a dashed cyan box in (A). Residue W314 is shown in blue and GlcNAc in yellow, two orientations 90 degrees apart are provided. (D) Average number of GlcNAc monomers bound to PgaA residues. The residues with the three highest average numbers of bound ligand are labelled. (E) Distribution of the plane rotation angle θ1 for the conformational basin highlighted by the dashed cyan box in (A). (PNG) [file ppat.1010750.s015.png]

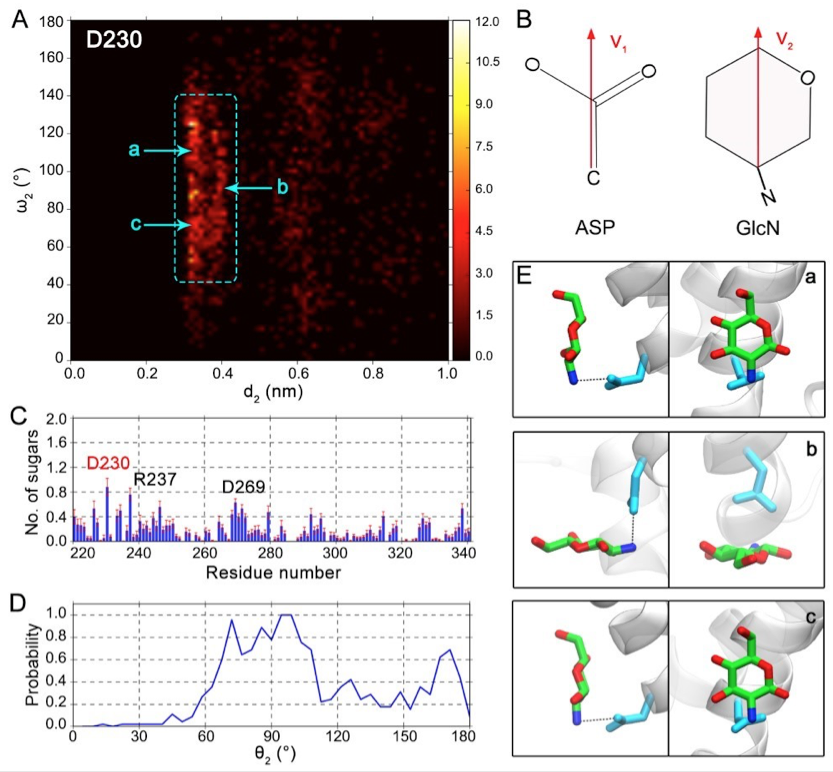

Supplement: S16 Fig — (A) 2D histogram of the distributions of plane tilt angle ω2 and distance d2 for GlcN binding to D230. (B) Schematic illustration of the plane rotation angle θ2, which is defined as the angle between V1 and V2. (C) Average number of GlcN monomers bound to PgaA residues. (D) Distribution of the plane rotation angle θ2 for the basin of conformations highlighted by a dashed cyan box in (A). (E) Representative snapshots of the basin of conformations highlighted by the dashed cyan box in (A). Residue D230 is shown in blue and GlcN in green, two orientations 90 degree apart are provided. (PNG) [file ppat.1010750.s016.png]

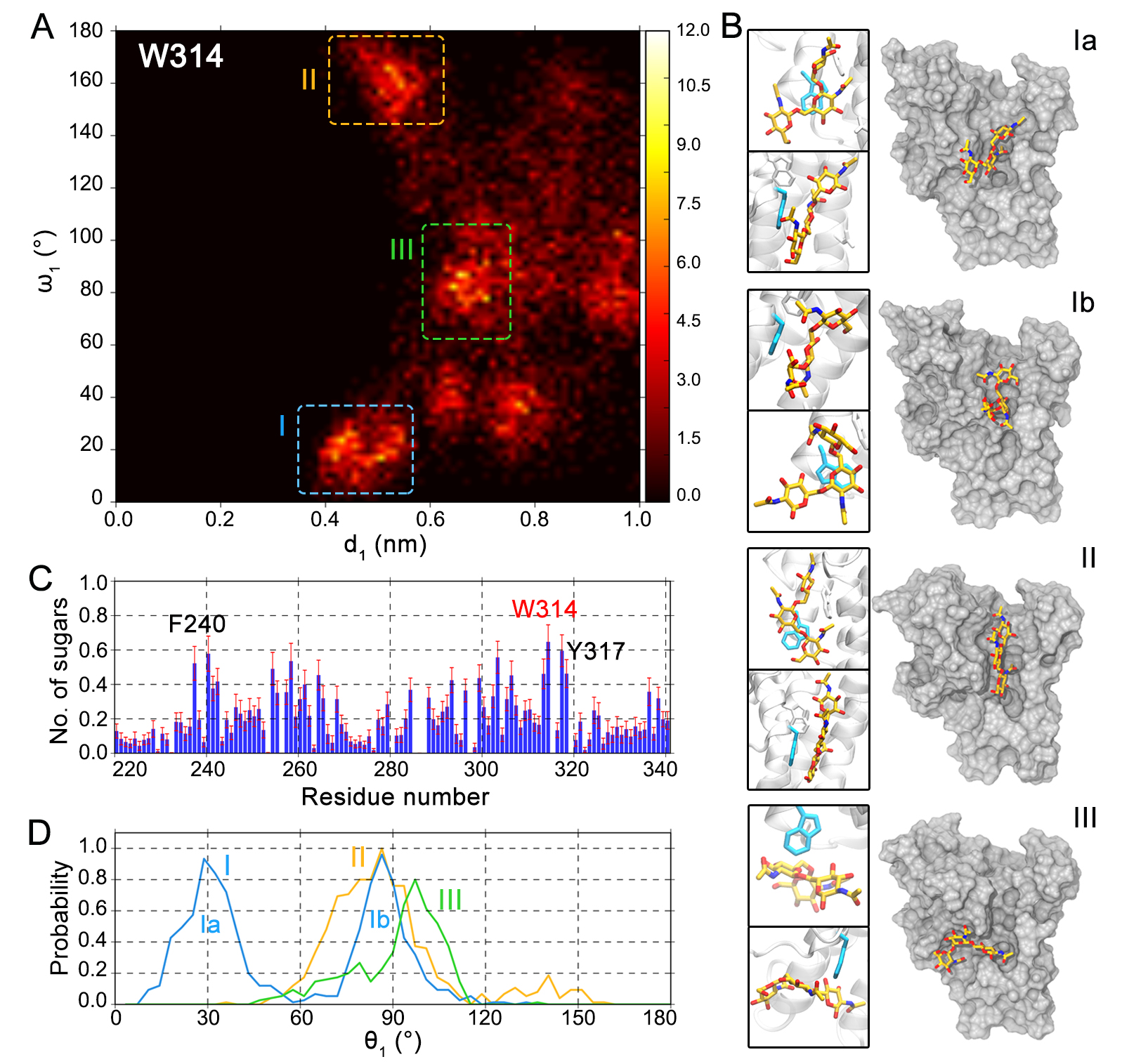

Supplement: S17 Fig — (A) 2D histogram of the distributions of plane tilt angle ω1 and plane distance d1 for (GlcNAc)3 binding to W314. The populations of conformational basins I, II, and III are 11 ± 4%, 5 ± 2%, and 10 ± 3%, respectively. (B) Representative snapshots of basins I, II and III. Residue W314 is shown in blue and (GlcNAc)3 in yellow, two orientations 90 degrees apart are provided as well as a view of the entire TPR module. (C) Average number of (GlcNAc)3 trimers bound to PgaA residues. (D) Distribution of the plane rotation angle θ1 for basins I, II and III. (JPG) [file ppat.1010750.s017.jpg]

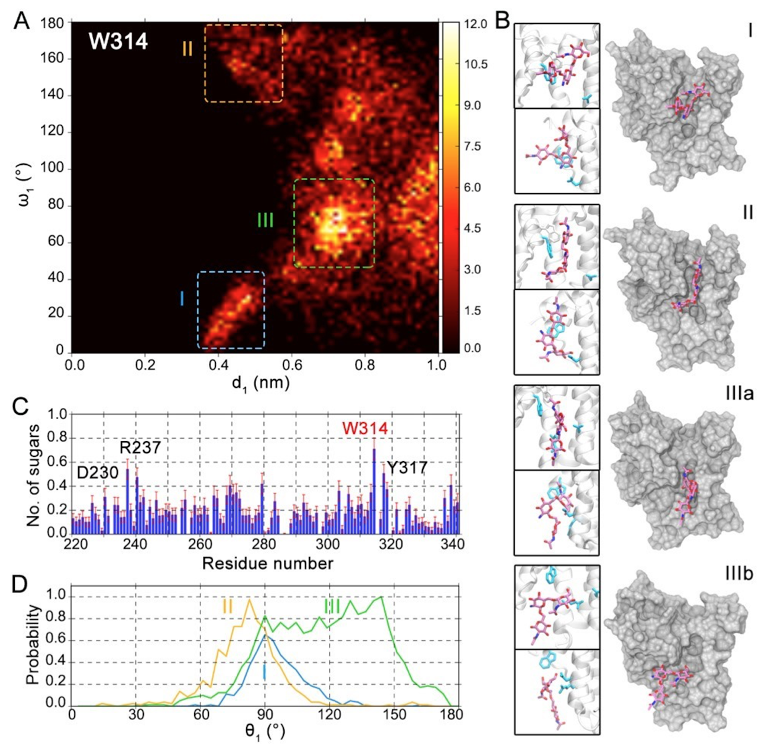

Supplement: S18 Fig — (A) 2D histogram of the distributions of plane tilt angle ω1 and plane distance d1 for GlcNAc-GlcN-GlcNAc binding to W314. Populations of basins I, II, and III are 3 ± 2%, 3 ± 2%, and 10 ± 3%, respectively. (B) Representative snapshots of conformational basins I, II and III. Residue W314 is shown in blue and GlcNAc-GlcN-GlcNAc in pink; two orientations 90 degrees apart are provided as well as a view of the entire TPR module. (C) Averaged number of GlcNAc-GlcN-GlcNAc trimers bound to PgaA residues. (D) Distribution of the plane rotation angle θ1 for conformational basins I, II and III. (PNG) [file ppat.1010750.s018.png]

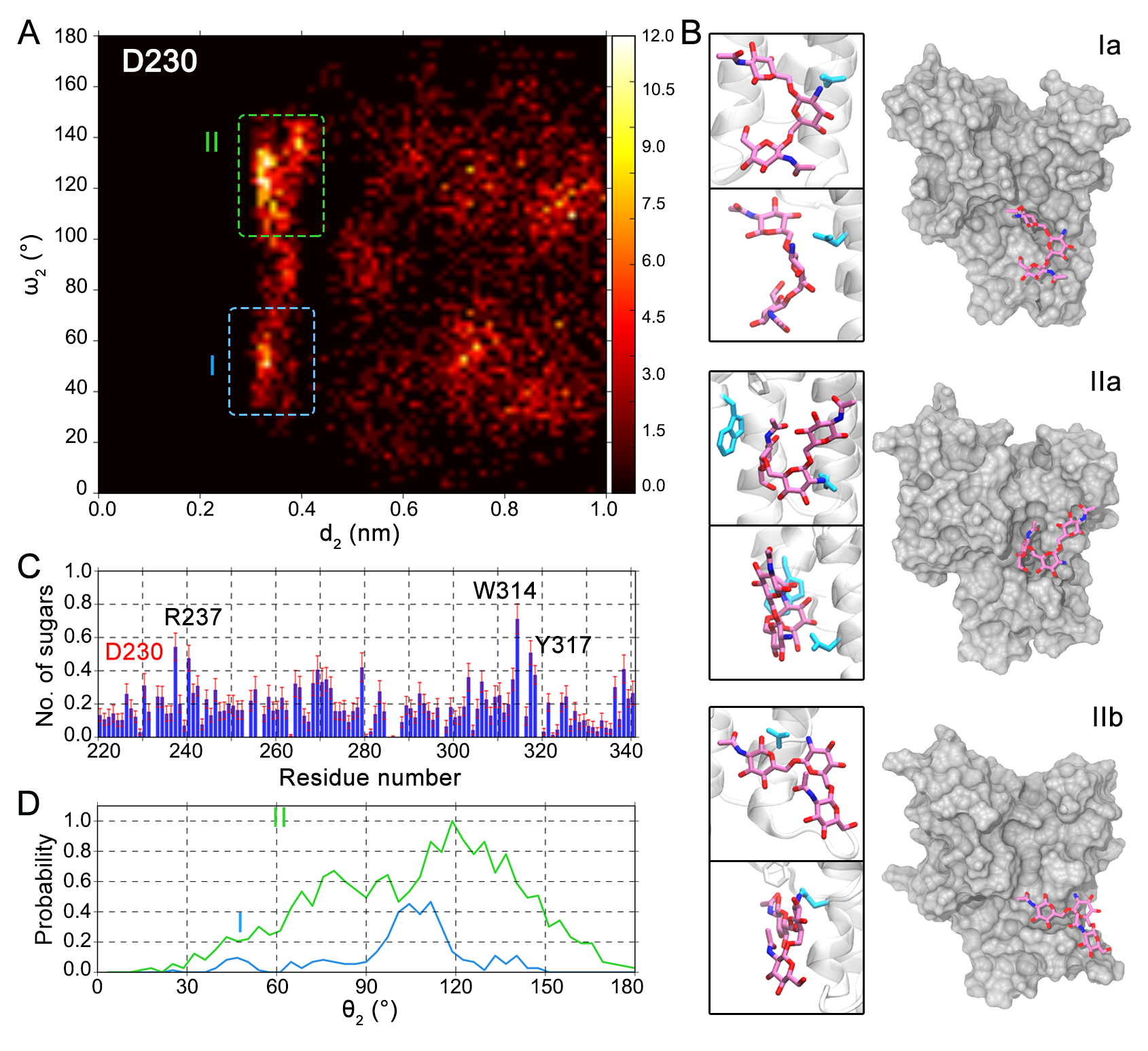

Supplement: S19 Fig — (A) 2D histogram of the distributions of plane tilt angle ω2 and distance d2 for GlcNAc-GlcN-GlcNAc binding to D230. Populations of conformational basins I, and II are 11 ± 4% and 5 ± 2%, respectively. (B) Representative snapshots of binding modes I and II. Residues W314 and D230 are shown in blue and GlcNAc-GlcN-GlcNAc in pink; two orientations 90 degree apart are provided as well as a view of the entire TPR module. (C) Average number of GlcNAc-GlcN-GlcNAc trimers bound to PgaA residues. (D) Distribution of the plane rotation angle θ2 for the conformation basins I and II defined in (A). (JPG) [file ppat.1010750.s019.jpg]

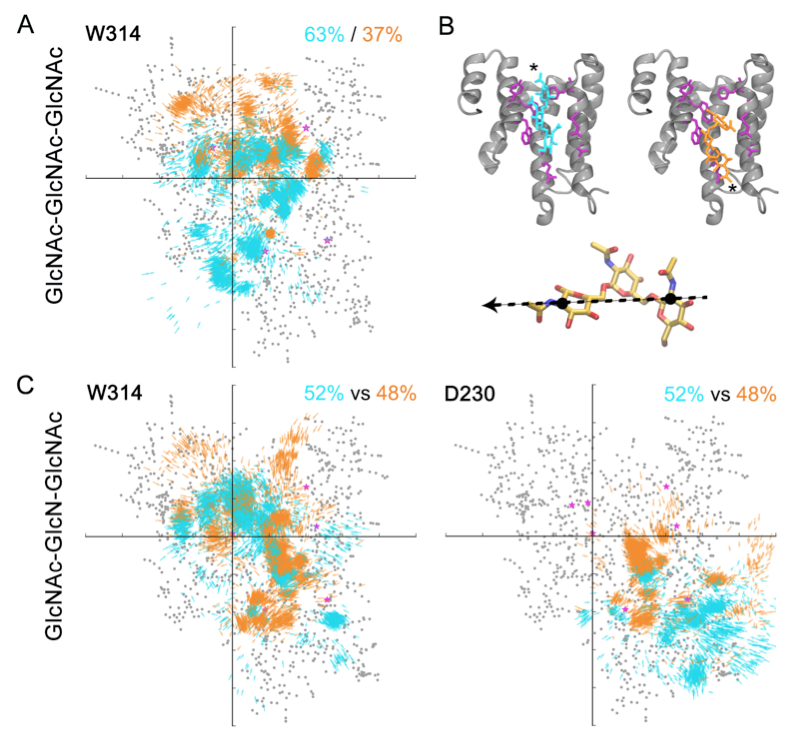

Supplement: S20 Fig — Projection of interactions of PgaA-220-340 with (A) (GlcNAc)3 or (C) GlcNAc-GlcN-GlcNAc on a 2D plane. All sugar conformations that involve interaction with either W314 or D230 are projected. The protein residues are projected as grey dots, with residues W314, W318, R279, R237, D230, F240 and Y317 highlighted as red stars. The coordinate origin is centered on W314. Sugar trimers are projected as orange and cyan arrows, corresponding to conformations with reducing ends pointing downward and upward, respectively. (B) Representative snapshots of sugar conformations with different orientations of reducing end (indicated by a black star), and the definition of arrows. The populations of conformations with reducing ends pointing downward (orange) or upward (cyan) are included at the top right corner of panels. The definition of the vector used for this analysis is shown by a dashed arrow. (PNG) [file ppat.1010750.s020.png]

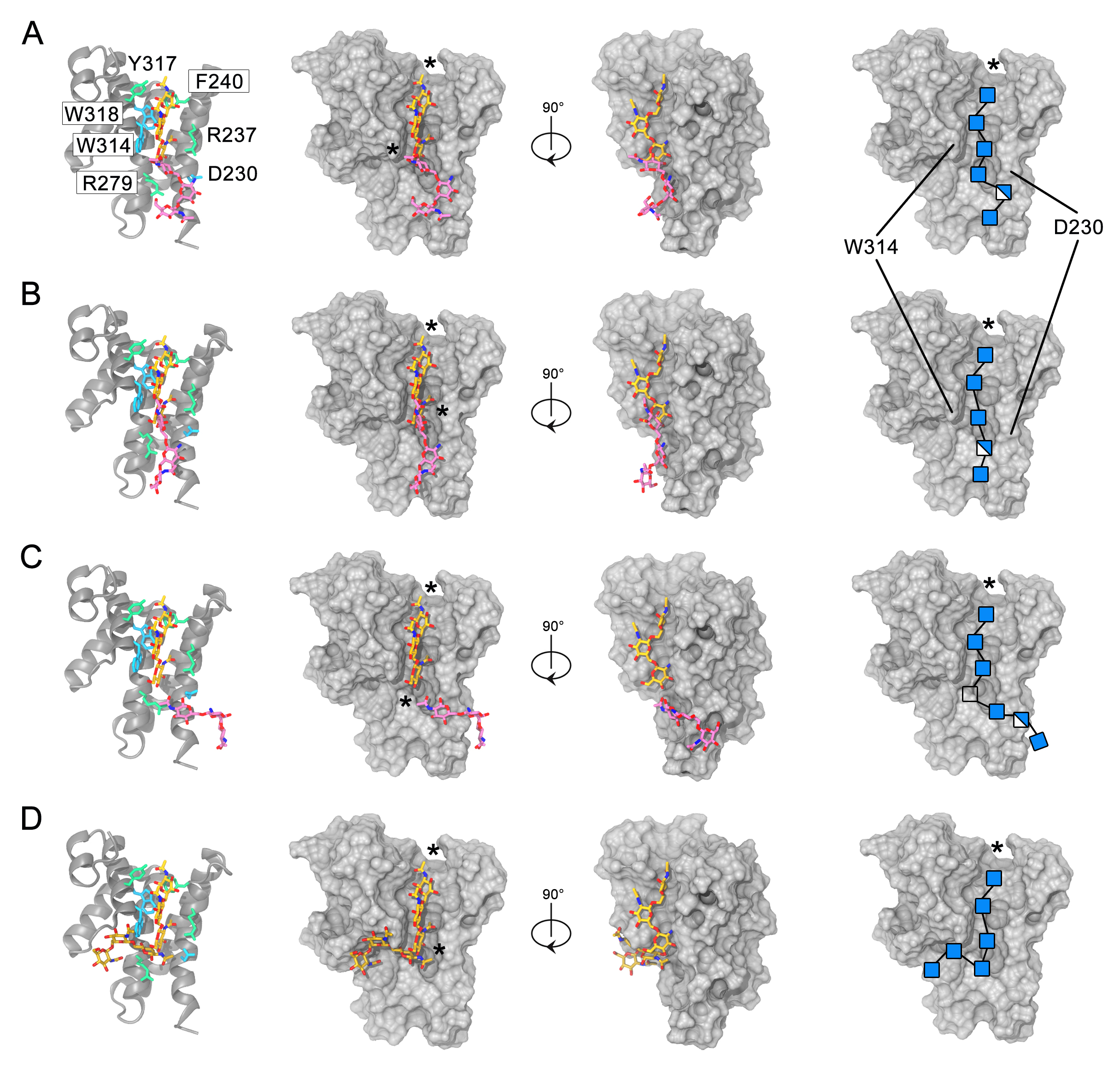

Supplement: S21 Fig — Potential pathways are labelled as A, B, C, and D. Residues that are frequently involved in contacts with dPNAG, including D230, R237, F240, R279, W314, Y317, and W318, are highlighted in the cartoon representation in the first column from left. Snapshots of sugar trimers adopted from either the same simulation or other simulations were imposed on the protein structure, as shown in the second and third columns. Schematic representations of potential dPNAG pathways are shown in the fourth column (right). The star indicates the reducing end of the trimer. (JPG) [file ppat.1010750.s021.jpg]

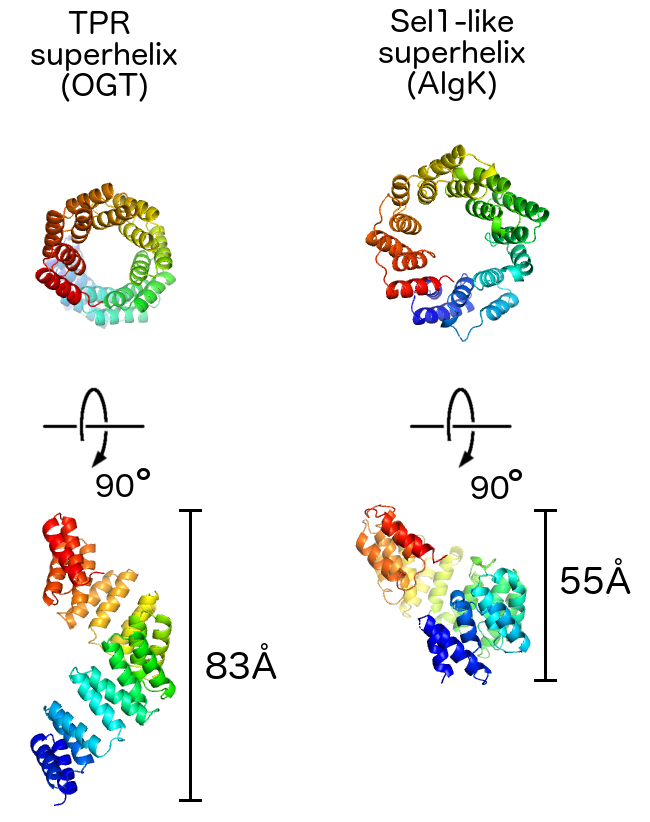

Supplement: S22 Fig — The left side shows OGT (PDB 1W3B, residues 77–383) [18] as an example for a TPR superhelix. The right side shows AlgK (PDB 3E4B, residues 71–388) [3] as an example for a Sel1-like superhelix. Both proteins contain 9 repeat motifs. At the top is a view along the superhelical axis, at the bottom is a side view. Both proteins are in rainbow colors with the N-terminus in blue and the C-terminus in red. (PNG) [file ppat.1010750.s022.png]

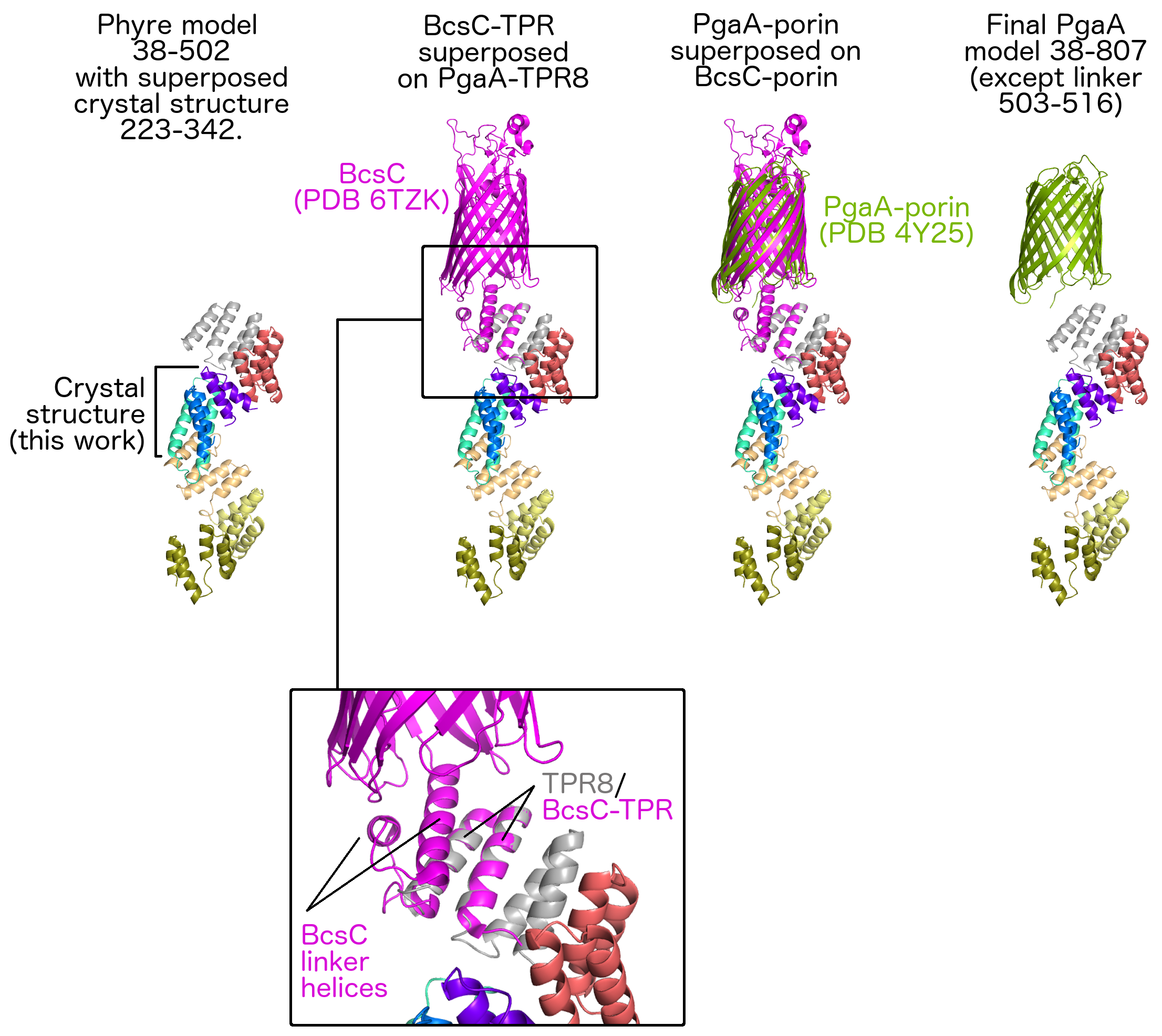

Supplement: S23 Fig — The Phyre2 model [21] of PgaA-38-502 is based on the crystal structure of OGT (PDB 1W3B) [18]. The orientation of the PgaA porin (PDB 4Y25) [6] towards the TPR domain is based on a superposition of a terminal TPR motif from a BcsC crystal structure (PDB 6TZK) [20] onto TPR8. (PNG) [file ppat.1010750.s023.png]

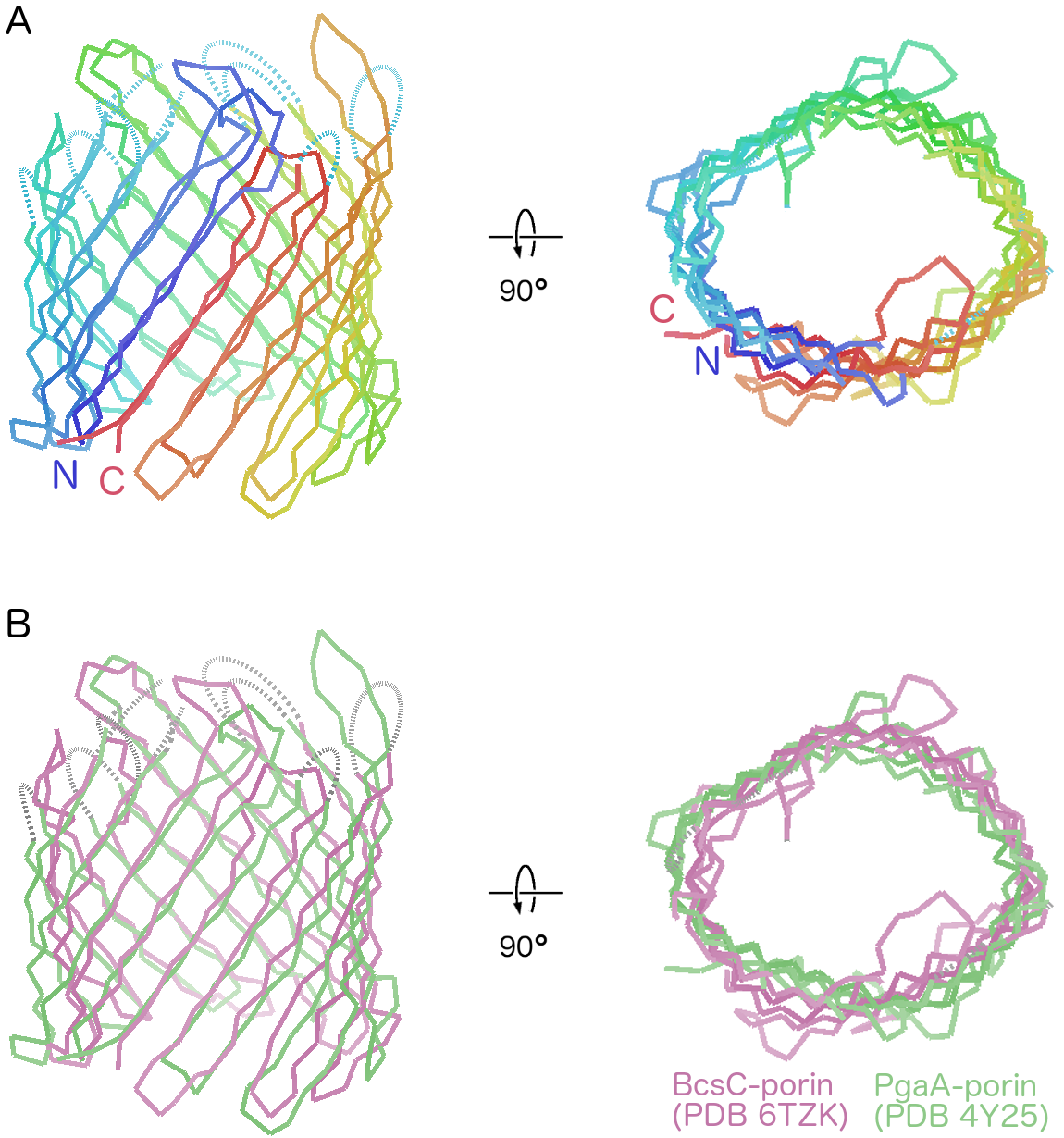

Supplement: S24 Fig — The sequence identity between both porins is 10.6%. Secondary-structure matching (SSM) alignment [58] is possible (rmsdCα = 2.9 Å) when most extracellular loops are ignored, excluded loops shown as dotted lines. (A) Rainbow color scheme from the N-terminal end (blue) to the C-terminal end (red) shows that all 16 beta-strands align in register (strand 1 of BcsC with strand 1 of PgaA, etc). (B) Coloring by chain reveals slight differences in the oval shape, explaining the elevated rmsd value. Alignments were performed in Coot [38]. (PNG) [file ppat.1010750.s024.png]

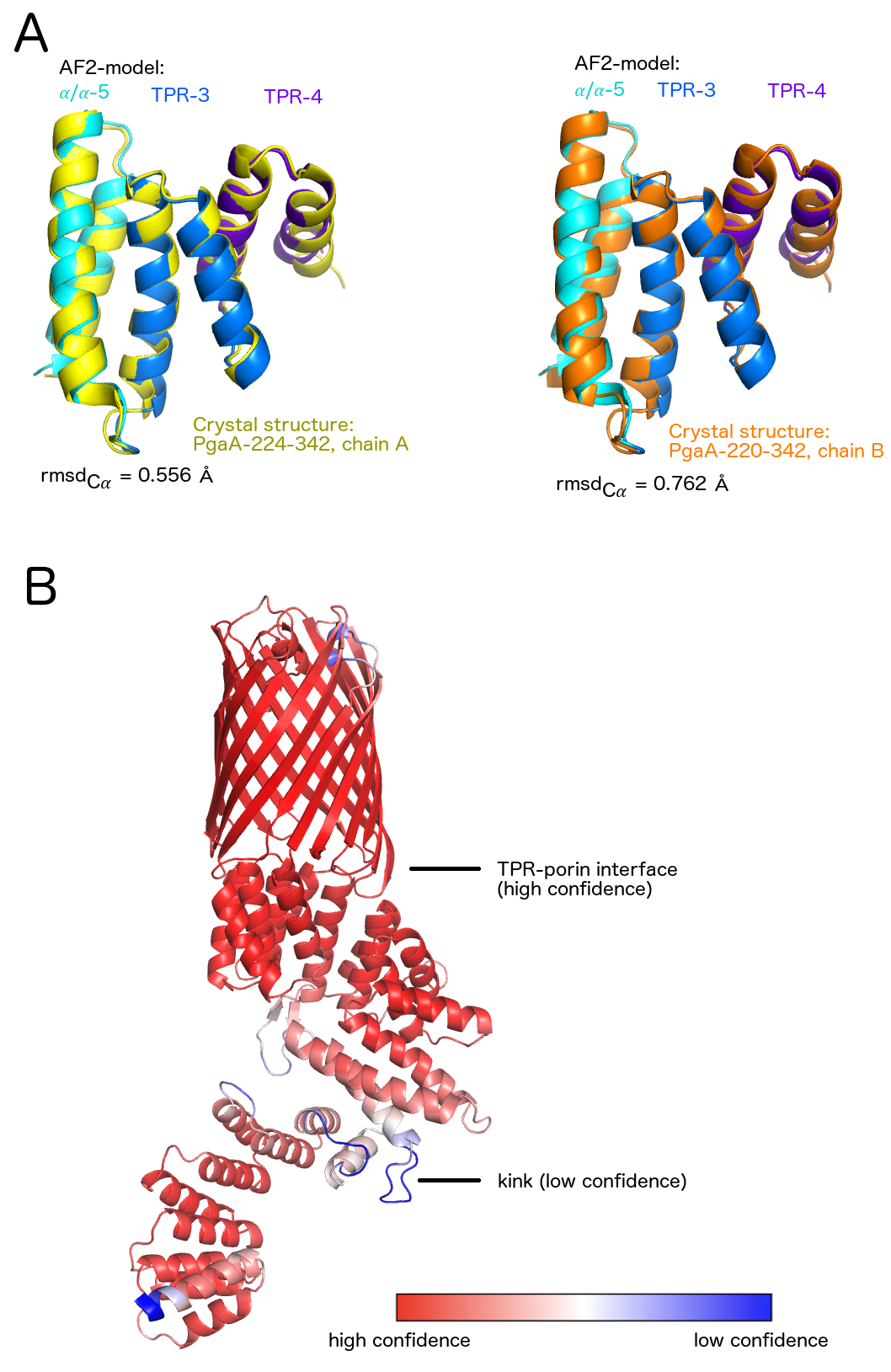

Supplement: S25 Fig — A) Comparison between crystal structure and AF2 model of PgaA-224-342. B) Confidence score of AF2 model of PgaA-32-807. The protein is colored by confidence level; solid red denotes high confidence (>90), solid blue denotes low confidence (<50). (PNG) [file ppat.1010750.s025.png]

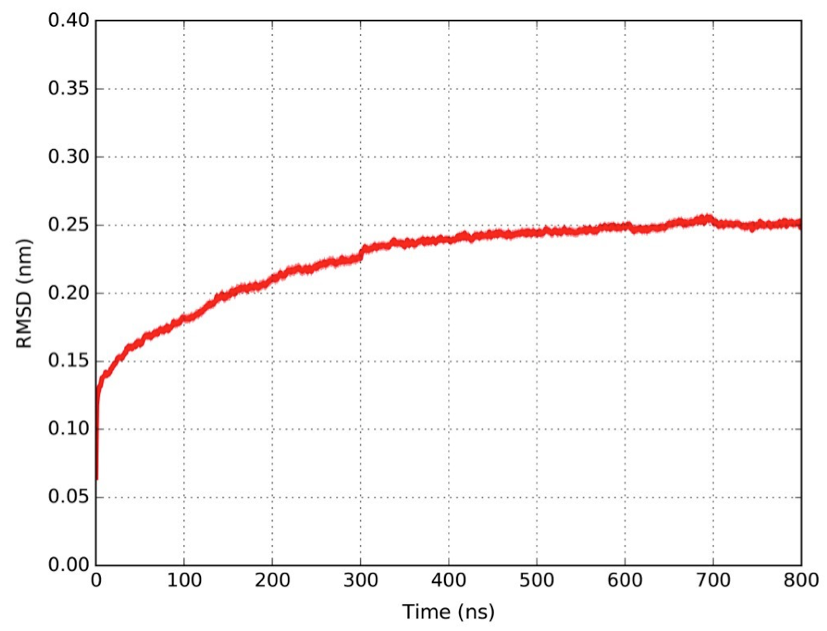

Supplement: S26 Fig — RMSD was computed for protein Cα atoms relative to the corresponding crystal structure PgaA-220-340. RMSD values in the time ranges of 0–300 ns and of 300–800 ns were averaged over all datasets and only datasets of sugar trimers, respectively. The RMSD value reached 0.25 nm after 800 ns. Standard deviation of mean was shown in red shadow. (PNG) [file ppat.1010750.s026.png]
