# Supplementary material for: The TPR domain of PgaA is a multifunctional scaffold that binds PNAG and modulates PgaB-dependent polymer processing
Source: PLoS Pathog. 2022 Aug 5;18(8):e1010750. doi: 10.1371/journal.ppat.1010750 (PMC9384988; doi:10.1371/journal.ppat.1010750)
Supplement: S1 Table — (DOCX) [file ppat.1010750.s027.docx]

**S1 Table.** Summary of simulated systems.

| Systems | Repeats | Time scale per repeat (ns) | Total time scale (μs) | Top 5 interacting residues |
| --- | --- | --- | --- | --- |
| PgaA + GlcNAc | 20 | 300 | 6 | R237, F240, R279, I310, W314 |
| PgaA + GlcN | 20 | 300 | 6 | D230, R237, E246, D269, E338 |
| PgaA + (GlcNAc)_3_ | 40 | 800 | 32 | R237, F240, R303, W314, Y317 |
| PgaA + (GlcNAc-GlcN-GlcNAc) | 40 | 800 | 32 | R237, F240, R279, W314, Y317 |
