# Supplementary material for: The TPR domain of PgaA is a multifunctional scaffold that binds PNAG and modulates PgaB-dependent polymer processing
Source: PLoS Pathog. 2022 Aug 5;18(8):e1010750. doi: 10.1371/journal.ppat.1010750 (PMC9384988; doi:10.1371/journal.ppat.1010750)
Supplement: S2 Table — (DOCX) [file ppat.1010750.s028.docx]

**S2 Table.** List of strains, plasmids, and primers used in this report.

| Strain, plasmid, or primer | Description or characteristics | Source or reference |
| --- | --- | --- |
| Strains  B834 | *E. coli* (DE3) strain for seleno-methionine incorporation | Novagen |
| TOP10 | *E. coli* cloning strain: F^-^ *mcr*A D(*mrr-hsd*RMS-*mcr*BC) f80*lac*ZDM15 D*lac*X74 *rec*A1 *ara*D139 D(*ara-leu*) 7697 *gal*U *gal*K *rps*L (Str^r^) *end*A1 *nup*G l- | Invitrogen |
| BL21 CodonPlus (DE3) | *E. coli* expression strain: F^-^ *omp*T *hsd*S(r_B_^-^ m_B_^-^) *dcm*^+^ Tet^r^ *gal* l(DE3) *end*A [*arg*U *pro*L Cam^r^]Tet^r^ *gal* l(DE3) *end*A [*arg*U *pro*L Cam^r^] | Stratagene |
| Plasmids | | |
| pET24a | Expression vector | Novagen |
| pET26b | Expression vector | Novagen |
| pET28a | Expression vector | Novagen |
| pETDL | Peri/OM expression vector, pET26b with mutated NdeI site, and the pET28a MCS subcloned into the NcoI and XhoI sites. | This study |
| pQLinkN | Coexpression vector | Addgene |
| pPGA372 | *pgaABCD* in pUC19 | [1] |
| pET28a-PgaA | PgaA-32-807 expression plasmid (cytoplasm) | This study |
| pET26b-PgaA | PgaA-33-807 expression plasmid (membrane, non-cleavable His6-tag, Q130R N194D mutations) | This study |
| pETDL-PgaA | PgaA-33-807 expression plasmid (membrane) | This study |
| pET28-PgaA-32-367 | PgaA-32-367 expression plasmid (cytoplasm) | This study |
| pET28a-PgaA-220-367 | PgaA-220-367 expression plasmid (cytoplasm) | This study |
| pET28a-PgaA-32-220 | PgaA-32-220 expression plasmid (cytoplasm) | This study |
| pET28a-PgaA-368-515 | PgaA-368-515 expression plasmid (cytoplasm) | This study |
| pET28a-PgaA-368-502 | PgaA-368-502 expression plasmid (cytoplasm) | This study |
| pET28a-PgaA-368-482 | PgaA-368-482 expression plasmid (cytoplasm) | This study |
| pET28a-PgaA-352-502 | PgaA-352-502 expression plasmid (cytoplasm) | This study |
| pET28a-PgaA-352-464 | PgaA-352-464 expression plasmid (cytoplasm) | This study |
| pET28a-PgaA-401-515 | PgaA-401-515 expression plasmid (cytoplasm) | This study |
| pET28a-PgaB-22-672 | PgaA-22-672 expression plasmid (cytoplasm) | [2] |
| pQLink-PgaA-32-807/PgaB | PgaA-32-807/PgaB co-expression plasmid | This study |
| pQLink-PgaA-221-807/PgaB | PgaA-221-807/PgaB co-expression plasmid | This study |
| pQLink-PgaA-Δ514/PgaB | PgaA-515-807/PgaB co-expression plasmid | This study |
| pQLink-PgaA-221-807/PgaB C21S | PgaA-221-807/PgaB C21S co-expression plasmid | This study |
| pQLink-PgaA-32-515/PgaB | PgaA-32-515/PgaB co-expression plasmid | This study |
| pQLink-PgaA-32-807/PgaB-D115A | PgaA-32-807/PgaB D115A co-expression plasmid | This study |
| Primers | | |
| PgaA 32 F | GGG**CATATG**AGTGCTGTTAATAAC | This study |
| PgaA His6 33 F | GGG**CCATGG**GCCACCACCACCACCACCATAATCTTGCAGAGC | This study |
| PgaA 807 R | GGG**AAGCTT**TTAAAATCTGAATGTCAT | This study |
| PgaA-PelB leader F | GGTGGTGGATCCATGAAATACCTGCTGCCGAC | This study |
| PgaA 807 R* | GGTGGT**GCGGCCGC**TTAAAATCTGAATGTCATATCGAATTCAACG | This study |
| PgaA 32 R | CATATGGCTGCCGCGC | This study |
| PgaA 221 F | GCCAATTTAACGCCAGATATTCGCG | This study |
| PgaA514 R | ATGTACATCAACCGCACGCTTTAATC | This study |
| PgaA 807 F | TAAGCTTAATTAGCTGAGCTTGGACTCC | This study |
| PgaA 368 F | GGTGGT**CATATG**TATCCGGGCGCGCTAAC | This study |
| PgaA 515 R | GGTGGT**AAGCTT**TCAATGTACATCAACCGCACGTTTTAATC | This study |
| PgaA 502 R | GGTGGT**AAGCTT**TCAATCTTGCGGTTCACGTTCGAC | This study |
| PgaA 352 F | GGTGGT**CATATG**GATGAAGAACTTGCGGATCTCTTTTAC | This study |
| PgaA 401 F | GGTGGT**CATATG**TTACAGGGGCATTCGTTTCTCTC | This study |
| PgaB 1 F | CC**GGATCC**ATGTTACGTAATGGAAATAAATATCTCCTGATGC | This study |
| PgaB 672 R | CCT**AAGCTT**TTAATCATTTTTCGGATACCAGGCTGTTG | This study |
| PgaB C21S F | ATGCTCACCGCGAGCATTAGCCAGTCAAGAAC | This study |
| PgaB C21S R | GTTCTTGACTGGCTAATGCTCGCGGTGAGCAT | This study |
| PgaA 368Stop F | GGAGAGTGAAAATTAACCGGGCGCGC | This study |
| PgaA 368Stop R | GCGCGCCCGGTTAATTTTCACTCTCC | This study |
| PgaA 220stop F | GCTGCCGCGATTGACGATTGAAATTTAACGCCAGATATTCG | This study |
| PgaA 220stop R | CGAATATCTGGCGTTAAATTTCAATCGTCAATCGCGGCAGC | This study |
| PgaA 464stop F | CAGAAGTGATCTAGCCACG | This study |
| PgaA 464stop R | CGTGGCTAGATCACTTCTG | This study |
| PgaA 482stop F | CATTAACGTTATAAGAATGGCAG | This study |
| PgaA 482stop R | CTGCCATTCTTATAACGTTAATG | This study |

Restriction enzyme sites are in **bold**

**References**

1. Wang X, Preston JF, Romeo T. The pgaABCD Locus of Escherichia coli Promotes the Synthesis of a Polysaccharide Adhesin Required for Biofilm Formation. J Bacteriol. 2004;186: 2724–2734. doi:10.1128/jb.186.9.2724-2734.2004.
2. Little DJ, Whitney JC, Robinson H, Yip P, Nitz M, Howell PL. Combining in situ proteolysis and mass spectrometry to crystallize Escherichia coli PgaB. Acta Crystallogr Sect F Struct Biology Cryst Commun. 2012;68: 842–5. doi:10.1107/s1744309112022075.
